# Multiscale analysis of slow-fast neuronal learning models with noise

**DOI:** 10.1186/2190-8567-2-13

**Published:** 2012-11-22

**Authors:** Mathieu Galtier, Gilles Wainrib

**Affiliations:** 1 NeuroMathComp Project Team, INRIA/ENS Paris, 23 avenue d’Italie, Paris, 75013, France; 2 School of Engineering and Science, Jacobs University Bremen gGmbH, College Ring 1, P.O. Box 750 561, Bremen, 28725, Germany; 3 Laboratoire Analyse Géométrie et Applications, Université Paris 13, 99 avenue Jean-Baptiste Clément, Villetaneuse, Paris, France

**Keywords:** slow-fast systems, stochastic differential equations, inhomogeneous Markov process, averaging, model reduction, recurrent networks, unsupervised learning, Hebbian learning, STDP

## Abstract

This paper deals with the application of temporal averaging methods to recurrent networks of noisy neurons undergoing a slow and unsupervised modification of their connectivity matrix called learning. Three time-scales arise for these models: (i) the fast neuronal dynamics, (ii) the intermediate external input to the system, and (iii) the slow learning mechanisms. Based on this time-scale separation, we apply an extension of the mathematical theory of stochastic averaging with periodic forcing in order to derive a reduced deterministic model for the connectivity dynamics. We focus on a class of models where the activity is linear to understand the specificity of several learning rules (Hebbian, trace or anti-symmetric learning). In a weakly connected regime, we study the equilibrium connectivity which gathers the entire ‘knowledge’ of the network about the inputs. We develop an asymptotic method to approximate this equilibrium. We show that the symmetric part of the connectivity post-learning encodes the correlation structure of the inputs, whereas the anti-symmetric part corresponds to the cross correlation between the inputs and their time derivative. Moreover, the time-scales ratio appears as an important parameter revealing temporal correlations.

## 1 Introduction

Complex systems are made of a large number of interacting elements leading to non-trivial behaviors. They arise in various areas of research such as biology, social sciences, physics or communication networks. In particular in neuroscience, the nervous system is composed of billions of interconnected neurons interacting with their environment. Two specific features of this class of complex systems are that (i) external inputs and (ii) internal sources of random fluctuations influence their dynamics. Their theoretical understanding is a great challenge and involves high-dimensional non-linear mathematical models integrating non-autonomous and stochastic perturbations.

Modeling these systems gives rise to many different scales both in space and in time. In particular, learning processes in the brain involve three time-scales: from neuronal activity (fast), external stimulation (intermediate) to synaptic plasticity (slow). Here, fast time-scale corresponds to a few milliseconds and slow time-scale to minutes/hour, and intermediate time-scale generally ranges between fast and slow scales, although some stimuli may be faster than neuronal activity time-scale (*e.g.*, submilliseconds auditory signals [1]). The separation of these time-scales is an important and useful property in their study. Indeed, multiscale methods appear particularly relevant to handle and simplify such complex systems. 

 First, stochastic averaging principle [2,3] is a powerful tool to analyze the impact of noise on slow-fast dynamical systems. This method relies on approximating the fast dynamics by its quasi-stationary measure and averaging the slow evolution with respect to this measure. In the asymptotic regime of perfect time-scale separation, this leads to a slow reduced system whose analysis enables a better understanding of the original stochastic model. 

 Second, periodic averaging theory [4], which has been originally developed for celestial mechanics, is particularly relevant to study the effect of fast deterministic and periodic perturbations (external input) on dynamical systems. This method also leads to a reduced model where the external perturbation is time-averaged. 

It seems appropriate to gather these two methods to address our case of a noisy and input-driven slow-fast dynamical system. This combined approach provides a novel way to understand the interactions between the three time-scales relevant in our models. More precisely, we will consider the following class of multiscale stochastic differential equations (SDEs), with $\epsilon_{1},\epsilon_{2}>0$ two small parameters

(1) $$\left\{ \begin{array}{c} dv^{\epsilon }=\frac{1}{\epsilon_{1}}[F(v^{\epsilon },w^{\epsilon },u(\frac{t}{\epsilon_{2}}))]\;dt+\frac{1}{\sqrt{\epsilon_{1}}}\Sigma \;dB(t),\\ dw^{\epsilon }=G(v^{\epsilon },w^{\epsilon })\;dt, \end{array}\right.$$

where $v^{\epsilon }\in R^{p}$ represents the fast activity of the individual elements, $w^{\epsilon }\in R^{q}$ represents the connectivity weights that vary slowly due to plasticity, and $u(t)\in R^{p}$ represents the value of the external input at time *t*. Random perturbations are included in the form of a diffusion term, and (B(t)) is a standard Brownian motion.

We are interested in the double limit $\epsilon_{1}\to 0$ and $\epsilon_{2}\to 0$ to describe the evolution of the slow variable **w** in the asymptotic regime where both the variable **v** and the external input are much faster than **w**. This asymptotic regime corresponds to the study of a neuronal network in which both the external input **u** and the neuronal activity **v** operate on a faster time-scale than the slow plasticity-driven evolution of synaptic weights **W**. To account for the possible difference of time-scales between **v** and the input, we introduce the time-scale ratio $\mu =\epsilon_{1}/\epsilon_{2}\in [0,\infty ]$. In the interesting case where μ∈(0,∞), one needs to understand the long-time behavior of the rescaled periodically forced SDE for any $w_{0}$ fixed

$$dv=F(v,w_{0},\mu t)\;dt+\Sigma (v,w_{0})\;dB(t).$$

Recently, in an important contribution [5], a precise understanding of the long-time behavior of such processes has been obtained using methods from partial differential equations. In particular, conditions ensuring the existence of a periodic family of probability measures to which the law of **v** converges as time grows have been identified, together with a sharp estimation of the speed of mixing. These results are at the heart of the extension of the classical stochastic averaging principle [2] to the case of periodically forced slow-fast SDEs [6]. As a result, we obtain a reduced equation describing the slow evolution of variable **w** in the form of an ordinary differential equation,

$$\frac{dw}{dt}=\bar{G}(w),$$

where $\bar{G}$ is constructed as an average of *G* with respect to a specific probability measure, as explained in Section 2.

This paper first introduces the appropriate mathematical framework and then focuses on applying these multiscale methods to learning neural networks.

 The individual elements of these networks are neurons or populations of neurons. A common assumption at the basis of mathematical neuroscience [7] is to model their behavior by a stochastic differential equation which is made of four different contributions: (i) an intrinsic dynamics term, (ii) a communication term, (iii) a term for the external input, and (iv) a stochastic term for the intrinsic variability. Assuming that their activity is represented by the fast variable $v\in R^{n}$, the first equation of system (1) is a generic representation of a neural network (function *F* corresponds to the first three terms contributing to the dynamics). In the literature, the level of non-linearity of the function *F* ranges from a linear (or almost-linear) system to spiking neuron dynamics [8], yet the structure of the system is universal. 

These neurons are interconnected through a connectivity matrix which represents the strength of the synapses connecting the real neurons together. The slow modification of the connectivity between the neurons is commonly thought to be the essence of learning. Unsupervised learning rules update the connectivity exclusively based on the value of the activity variable. Therefore, this mechanism is represented by the slow equation above, where $w\in R^{n\times n}$ is the connectivity matrix and *G* is the learning rule. Probably the most famous of these rules is the Hebbian learning rule introduced in [9]. It says that if both neurons A and B are active at the same time, then the synapses from A to B and B to A should be strengthened proportionally to the product of the activity of A and B. There are many different variations of this correlation-based principle which can be found in [10,11]. Another recent, unsupervised, biologically motivated learning rule is the spike-timing-dependent plasticity (STDP) reviewed in [12]. It is similar to Hebbian learning except that it focuses on causation instead of correlation and that it occurs on a faster time-scale. Both of these types of rule correspond to *G* being quadratic in **v**.

 Previous literature about dynamic learning networks is thick, yet we take a significantly different approach to understand the problem. An historical focus was the understanding of feedforward deterministic networks [13-15]. Another approach consisted in precomputing the connectivity of a recurrent network according to the principles underlying the Hebbian rule [16]. Actually, most of current research in the field is focused on STDP and is based on the precise times of the spikes, making them explicit in computations [17-20]. Our approach is different from the others regarding at least one of the following points: (i) we consider recurrent networks, (ii) we study the evolution of the coupled system activity/connectivity, and (iii) we consider bounded dynamical systems for the activity without asking them to be spiking. Besides, our approach is a rigorous mathematical analysis in a field where most results rely heavily on heuristic arguments and numerical simulations. To our knowledge, this is the first time such models expressed in a slow-fast SDE formalism are analyzed using temporal averaging principles. 

The purpose of this application is to understand what the network learns from the exposition to time-dependent inputs. In other words, we are interested in the evolution of the connectivity variable, which evolves on a slow time-scale, under the influence of the external input and some noise added on the fast variable. More precisely, we intend to explicitly compute the equilibrium connectivities of such systems. This final matrix corresponds to the knowledge the network has extracted from the inputs. Although the derivation of the results is mathematically tough for untrained readers, we have tried to extract widely understandable conclusions from our mathematical results and we believe this paper brings novel elements to the debate about the role and mechanisms of learning in large scale networks.

 Although the averaging method is a generic principle, we have made significant assumptions to keep the analysis of the averaged system mathematically tractable. In particular, we will assume that the activity evolves according to a linear stochastic differential equation. This is not very realistic when modeling individual neurons, but it seems more reasonable to model populations of neurons; see Chapter 11 of [7]. 

The paper is organized as follows. Section 2 is devoted to introducing the temporal averaging theory. Theorem 2.2 is the main result of this section. It provides the technical tool to tackle learning neural networks. Section 3 corresponds to application of the mathematical tools developed in the previous section onto the models of learning neural networks. A generic model is described and three different particular models of increasing complexity are analyzed. First, Hebbian learning, then trace-learning, and finally STDP learning are analyzed for linear activities. Finally, Section 4 is a discussion of the consequences of the previous results from the viewpoint of their biological interpretation.

## 2 Averaging principles: theory

In this section, we present multiscale theoretical results concerning stochastic averaging of periodically forced SDEs (Section 2.3). These results combine ideas from singular perturbations, classical periodic averaging and stochastic averaging principles. Therefore, we recall briefly, in Sections 2.1 and 2.2, several basic features of these principles, providing several examples that are closely related to the application developed in Section 3.

### 2.1 Periodic averaging principle

We present here an example of a slow-fast ordinary differential equation perturbed by a fast external periodic input. We have chosen this example since it readily illustrates many ideas that will be developed in the following sections. In particular, this example shows how the ratio between the time-scale separation of the system and the time-scale of the input appears as a new crucial parameter.

*Example 2.1* Consider the following linear time-inhomogeneous dynamical system with $\epsilon_{1},\epsilon_{2}>0$ two parameters:

$$\begin{array}{rcl} \frac{dv^{\epsilon }}{dt} & = & \frac{1}{\epsilon_{1}}\left(-v^{\epsilon }+\sin\left(\frac{t}{\epsilon_{2}}\right)\right),\\ \frac{dw^{\epsilon }}{dt} & = & -w^{\epsilon }+{v^{\epsilon }}^{2}. \end{array}$$

This system is particularly handy since one can solve analytically the first ordinary differential equation, that is,

$$v(t)=\frac{1}{1+\mu^{2}}\left(\sin\left(\frac{t}{\epsilon_{2}}\right)-\mu \cos\left(\frac{t}{\epsilon_{2}}\right)\right)+v_{0}e^{-\frac{t}{\epsilon_{1}}},$$

where we have introduced the *time-scales ratio*

$$\mu :=\frac{\epsilon_{1}}{\epsilon_{2}}.$$

In this system, one can distinguish various asymptotic regimes when $\epsilon_{1}$ and $\epsilon_{2}$ are small according to the asymptotic value of *μ*: 

• Regime 1: Slow input μ=0:

First, if $\epsilon_{1}\to 0$ and $\epsilon_{2}$ is fixed, then v(t) is close to $\sin(\frac{t}{\epsilon_{2}})$, and from *geometric singular perturbation theory*[21,22] one can approximate the slow variable $w^{\epsilon }$ by the solution of

$$\frac{dw}{dt}=-w+{\left(\sin\left(\frac{t}{\epsilon_{2}}\right)\right)}^{2}.$$

Now taking the limit $\epsilon_{2}\to 0$ and applying the classical *averaging principle*[4] for periodically driven differential equations, one can approximate $w^{\epsilon }$ by the solution of

$$\frac{dw}{dt}=-w+\frac{1}{2},$$

since $\frac{1}{2\pi }\int_{0}^{2\pi }\sin{\left(s\right)}^{2}\;ds=\frac{1}{2}$.

• Regime 2: Fast input μ=∞:

If $\epsilon_{2}\to 0$ and $\epsilon_{1}$ is fixed, then the classical averaging principle implies that $v^{\epsilon }$ is close to the solution of

$$\frac{dv}{dt}=-\frac{v}{\epsilon_{1}},$$

so that $w^{\epsilon }$ can be approximated by

$$\frac{dw}{dt}=-w+{\left(v_{0}e^{-t/\epsilon_{1}}\right)}^{2},$$

and when $\epsilon_{1}\to 0$, one does not recover the same asymptotic behavior as in Regime 1.

• Regime 3: Time-scales matching 0<μ<∞:

Now consider the intermediate case where $\epsilon_{1}$ is asymptotically proportional to $\epsilon_{2}$. In this case, $v^{\epsilon }$ can be approximated on the fast time-scale $t/\epsilon_{1}$ by the periodic solution ${\bar{v}}_{\mu }(t)=\frac{1}{1+\mu^{2}}(\sin(\mu t)-\mu \cos(\mu t))$ of $\frac{dv}{dt}=-v+\sin(\mu t)$. As a consequence, $w^{\epsilon }$ will be close to the solution of

$$\frac{dw}{dt}=-w+\frac{1}{2(1+\mu^{2})},$$

since $\frac{1}{2\pi }\int_{0}^{2\pi }{\bar{v}}_{\mu }{\left(t/\mu \right)}^{2}\;dt=\frac{1}{2(1+\mu^{2})}$.

Thus, we have seen in this example that 

1. the two limits $\epsilon_{1}\to 0$ and $\epsilon_{2}\to 0$ do not commute,

2. the ratio *μ* between the internal time-scale separation $\epsilon_{1}$ and the input time-scale $\epsilon_{2}$ is a key parameter in the study of slow-fast systems subject to a time-dependent perturbation.

### 2.2 Stochastic averaging principle

Time-scales separation is a key property to investigate the dynamical behavior of non-linear multiscale systems, with techniques ranging from averaging principles to geometric singular perturbation theory. This property appears to be also crucial to understanding the impact of noise. Instead of carrying a small noise analysis, a multiscale approach based on the *stochastic averaging principle*[2] can be a powerful tool to unravel subtle interplays between noise properties and non-linearities. More precisely, consider a system of SDEs in $R^{p+q}$:

$$\begin{array}{rcl} dv_{t}^{\epsilon } & = & \frac{1}{\epsilon }F\left(v_{t}^{\epsilon },w_{t}^{\epsilon }\right)\;dt+\frac{1}{\sqrt{\epsilon }}\Sigma \left(v_{t}^{\epsilon },w_{t}^{\epsilon }\right)\cdot dB(t),\\ dw_{t}^{\epsilon } & = & G\left(v_{t}^{\epsilon },w_{t}^{\epsilon }\right)\;dt, \end{array}$$

with initial conditions $v^{\epsilon }(0)=v_{0}$, $w^{\epsilon }(0)=w_{0}$, and where $w^{\epsilon }\in R^{q}$ is called the slow variable, $v^{\epsilon }\in R^{p}$ is the fast variable, with *F*, *G*, **Σ** smooth functions ensuring the existence and uniqueness for the solution $\left(v^{\epsilon },w^{\epsilon }\right)$, and B(t) a *p*-dimensional standard Brownian motion, defined on a filtered probability space (Ω,F,P). Time-scale separation in encoded in the small parameter *ϵ*, which denotes in this section a single positive real number.

In order to approximate the behavior of $\left(v^{\epsilon },w^{\epsilon }\right)$ for small *ϵ*, the idea is to average out the equation for the slow variable with respect to the stationary distribution of the fast one. More precisely, one first assumes that for each $w\in R^{q}$ fixed, the *frozen* fast SDE,

$$dv_{t}=F(v_{t},w)\;dt+\Sigma (v_{t},w)\cdot dB(t),$$

admits a unique invariant measure, denoted $\rho^{w}(dv)$. Then, one defines the averaged drift vector field $\bar{G}$

(2) $$\bar{G}(w):=\int_{R^{m}}G(v,w)\rho^{w}(dv)$$

and **w** the solution of $\frac{dw}{dt}=\bar{G}(w)$ with the initial condition $w(0)=y_{0}$. Under some dissipativity assumptions, the stochastic averaging principle [2] states: 

**Theorem 2.1***For any*δ>0*and*T>0,

(3) $$\lim_{\epsilon \to 0}P\left[\underset{t\in [0,T]}{\sup}{∥w_{t}^{\epsilon }-w_{t}∥}^{2}>\delta \right]=0.$$

As a consequence, analyzing the behavior of the deterministic solution **w** can help to understand useful features of the stochastic process $\left(v^{\epsilon },w^{\epsilon }\right)$.

*Example 2.2* In this example we consider a similar system as in Example 2.1, but with a noise term instead of the periodic perturbation. Namely, we consider $\left(v^{\epsilon },w^{\epsilon }\right)$ the solution of the system of SDEs,

$$\begin{array}{rcl} dv^{\epsilon } & = & -\frac{1}{\epsilon }v^{\epsilon }\;dt+\frac{\sigma }{\sqrt{\epsilon }}\;dB(t),\\ dw^{\epsilon } & = & \left(-w^{\epsilon }+{\left(v^{\epsilon }\right)}^{2}\right)\;dt, \end{array}$$

with ϵ>0 a small parameter and σ>0 a positive constant. From Theorem 2.1, the stochastic slow variable $w^{\epsilon }$ can be approximated in the sense of (3) by the deterministic solution *w* of

$$\frac{dw}{dt}=\int_{v\in R}\left(-w+v^{2}\right)\rho (dv),$$

where ρ(dv) is the stationary measure of the linear diffusion process,

dv=−vdt+σdB(t),

that is,

$$\rho (dv)=\frac{1}{\sigma \sqrt{\pi }}e^{-\frac{v^{2}}{\sigma^{2}}}.$$

Consequently, $w^{\epsilon }$ can be approximated in the limit ϵ→0 by the solution of

$$\frac{dw}{dt}=-w+\frac{\sigma^{2}}{2}.$$

Applying (3) leads to the following result: for any T>0 and δ>0,

$$\lim_{\epsilon \to 0}P\left[\underset{t\in [0,T]}{\sup}{\left|w_{t}^{\epsilon }-\left(y_{0}-\frac{\sigma^{2}}{2}\right)e^{-t}+\frac{\sigma^{2}}{2}\right|}^{2}>\delta \right]=0.$$

Interestingly, the asymptotic behavior of $w^{\epsilon }$ for small *ϵ* is characterized by a deterministic trajectory that depends on the strength *σ* of the noise applied to the system. Thus, the stochastic averaging principle appears particularly interesting when unraveling the impact of noise strength on slow-fast systems.

Many other results have been developed since, extending the set-up to the case where the slow variable has a diffusion component or to infinite-dimensional settings for instance, and also refining the convergence study, providing *homogenization* results concerning the limit of $\epsilon^{-1/2}(w^{\epsilon }-w)$ or establishing large deviation principles (see [23] for a recent monograph). However, fewer results are available in the case of non-homogeneous SDEs, that is, when the system is perturbed by an external time-dependent signal. This setting is of particular interest in the framework of stochastic learning models, and we present the main relevant mathematical results in the following section. 

### 2.3 Double averaging principle

Combining ideas of periodic and stochastic averaging introduced previously, we present here theoretical results concerning multiscale SDEs driven by an external time-periodic input. Consider $\left(v^{\epsilon },w^{\epsilon }\right)$ the solution of

(4) $$\begin{array}{rl} dv^{\epsilon } & =\frac{1}{\epsilon_{1}}\left[F\left(v^{\epsilon },w^{\epsilon },\frac{t}{\epsilon_{2}}\right)\right]\;dt+\frac{1}{\sqrt{\epsilon_{1}}}\Sigma \left(v^{\epsilon },w^{\epsilon }\right)\cdot dB(t),\\ dw^{\epsilon } & =G\left(v^{\epsilon },w^{\epsilon }\right)\;dt, \end{array}$$

with $t\to F(v,w,t)\in R^{p}$ a *τ*-periodic function and $\epsilon =(\epsilon_{1},\epsilon_{2})\in R_{+}^{2}$. Parameter $\epsilon_{1}$ represents the internal time-scale separation and $\epsilon_{2}$ the input time-scale. We consider the case where both $\epsilon_{1}$ and $\epsilon_{2}$ are small, that is, a strong time-scale separation between the fast variable $v^{\epsilon }\in R^{p}$ and the slow one $w^{\epsilon }\in R^{q}$, and a fast periodic modulation of the fast drift F(v,w,⋅).

We further denote z=(v,w).

**Definition 2.1** We define the asymptotic time-scale ratio

(5) $$\mu :=\lim_{|\epsilon |\to 0}\frac{\epsilon_{1}}{\epsilon_{2}}.$$

Accordingly, we denote $\lim_{|\epsilon |\to 0}^{\mu }$ the distinguished limit when $\epsilon_{1}\to 0$, $\epsilon_{2}\to 0$ with $\epsilon_{1}/\epsilon_{2}\to \mu$.

The following assumption is made to ensure existence and uniqueness of a strong solution to system (4). In the following, $\langle z_{1},z_{2}\rangle$ will denote the usual scalar product for vectors.

**Assumption 2.1** Existence and uniqueness of a strong solution

(i) The functions *F*, *G*, and **Σ** are locally Lipschitz continuous in the space variable **z**. More precisely, for any R>0, there exists a constant $\alpha_{R}$ such that

$$∥F(z)-F\left(z^{\prime }\right)∥\leq \alpha_{R}∥z-z^{\prime }∥\;\text{for any }z,z^{\prime }\in R^{p+q}\text{ with }∥z∥\leq R\text{ and }∥z^{\prime }∥\leq R.$$

(ii) There exists a constant R>0 such that

$$\underset{∥z∥>R,t>0}{\sup}\frac{\langle (F(z,t),G(z)),z\rangle }{{∥z∥}^{2}}<0.$$

To control the asymptotic behavior of the fast variable, one further assumes the following.

**Assumption 2.2** Asymptotic behavior of the fast process:

(i) The diffusion matrix **Σ** is bounded

$$\exists M_{\Sigma }>0\;\text{s.t. }\forall z,∥\Sigma (z)∥<M_{\Sigma }$$

and uniformly non-degenerate

$$\exists \eta_{0}>0\;\text{s.t. }\forall v,z\langle \Sigma (z)\cdot \Sigma {\left(z\right)}^{\prime }v,v\rangle \geq \eta_{0}{∥v∥}^{2}.$$

(ii) There exists $r_{0}<0$ such that for all t≥0 and for all $z,x\in R^{p+q}$,

$$\langle \nabla_{z}F(z,t)\cdot x,x\rangle \leq r_{0}{∥x∥}^{2}.$$

According to the value of $\mu \in \{0,R_{+}^{*},\infty \}$, the stochastic averaging principle is based on a description of the asymptotic behavior of various rescaled fast frozen processes. More precisely, under Assumptions 2.1 and 2.2, one can deduce that: 

• For any fixed $w_{0}\in R^{q}$ and $t_{0}>0$ fixed, the law of the rescaled time-homogeneous frozen process,

$$dv=F(v,w_{0},t_{0})\;dt+\Sigma (v,w_{0})\;dB(t),$$

converges exponentially fast to a unique invariant probability measure denoted by $\rho^{w_{0},t_{0}}(dv)$.

• For any fixed $w_{0}\in R^{q}$, there exists a $\frac{\tau }{\mu }$-periodic evolution system of measures $\nu_{\mu }^{w_{0}}(t,dv)$, different from $\rho^{w_{0},t}(dv)$ above, such that the law of the rescaled time-inhomogeneous frozen process,

(6) $$dv=F(v,w_{0},\mu t)\;dt+\Sigma (v,w_{0})\;dB(t),$$

converges exponentially fast towards $\nu_{\mu }^{w_{0}}(t,\cdot )$, uniformly with respect to $w_{0}$ (*cf.* the Appendix Theorem A.1).

• For any fixed $w_{0}\in R^{q}$, the law of the rescaled time-homogeneous frozen process,

$$dv=\bar{F}(v,w_{0})\;dt+\Sigma (v,w_{0})\;dB(t),$$

where $\bar{F}(v,w_{0}):=\tau^{-1}\int_{0}^{\tau }F(v,w_{0},t)\;dt$, converges exponentially fast towards a unique invariant probability measure denoted by ${\bar{\rho }}^{w_{0}}(dv)$.

 According to the value of *μ*, we introduce a vector field ${\bar{G}}_{\mu }$ which will play a role similar to $\bar{G}$ introduced in equation (2).

**Definition 2.2** We define ${\bar{G}}_{\mu }:R^{q}\to R^{q}$ as follows. In the time-scale matching case, that is, when 0<μ<∞, then

(7) $${\bar{G}}_{\mu }(w):={\left(\frac{\tau }{\mu }\right)}^{-1}\int_{0}^{\frac{\tau }{\mu }}\int_{v\in R^{p}}G(v,w)\nu_{\mu }^{w}(t,dv)\;dt.$$

*Notation* We may denote the periodic system of measures $\nu_{\mu }^{w}(t,dv)$ associated with (6) by $\nu_{\mu }^{w}[F,\Sigma ](t,dv)$ to emphasize its relationship with *F* and **Σ**. Accordingly, we may denote ${\bar{G}}_{\mu }(w)$ by ${\bar{G}}_{\mu }^{\left[F,\Sigma \right]}(w)$.

We are now able to present our main mathematical result. Extending Theorem 2.1, the following theorem describes the asymptotic behavior of the slow variable $w^{\epsilon }$ when ϵ→0 with $\epsilon_{1}/\epsilon_{2}\to \mu$. We refer to [6] for more details about the full mathematical proof of this result. 

**Theorem 2.2***Let*μ∈(0,∞). *If***w***is the solution of*

(8) $$\frac{dw}{dt}={\bar{G}}_{\mu }(w)\;\text{with }w(0)=w^{\epsilon }(0),$$

*then the following convergence result holds*, *for all*T>0*and*δ>0:

$$\lim_{|\epsilon |\to 0}^{\mu }P\left[\underset{t\in [0,T]}{\sup}{\left|w_{t}^{\epsilon }-w_{t}\right|}^{2}>\delta \right]=0.$$

Remark 2.1

1. The extremal cases μ=0 and μ=∞ are not covered in full rigor by Theorem 2.2. However, the study of the sequential limits $\epsilon_{1}\to 0$ followed by $\epsilon_{2}\to 0$ or $\epsilon_{2}\to 0$ followed by $\epsilon_{1}\to 0$ can be deduced from an appropriate combination of classical periodic and stochastic averaging theorems: 

• Slow input: If one considers the case where the limit $\epsilon_{1}\to 0$ is taken *first*, so that from Theorem 2.1 with fast variable $v^{\epsilon }$ and slow variables $w^{\epsilon }$ and *t* (with the trivial equation $\dot{t}=1$), $w^{\epsilon }$ is close in probability on finite time-intervals to the solution of the following inhomogeneous ordinary differential equation:

$$\frac{d\tilde{w}}{dt}=\int_{v\in R^{p}}G(v,\tilde{w})\rho^{\tilde{w},t/\epsilon_{2}}(dv):=\tilde{G}(\tilde{w},t/\epsilon_{2}).$$

Then taking the limit $\epsilon_{2}\to 0$, one can apply the deterministic averaging principle to the fast periodic vector field $\tilde{G}(w,t/\epsilon_{2})$, so that $\tilde{w}$ converges when $\epsilon_{2}\to 0$ to the solution of

$$\frac{dw}{dt}=\tau^{-1}\int_{0}^{\tau }\tilde{G}(v,w)\;dt={\bar{G}}_{0}(w),$$

where

$${\bar{G}}_{0}(w):=\tau^{-1}\int_{0}^{\tau }\int_{v\in R^{p}}G(v,w)\rho^{w,t}(dv)\;dt.$$

• Fast input: If the limit $\epsilon_{2}\to 0$ is taken first, one first has to perform a classical averaging of the periodic drift $F(v,w,t/\epsilon_{2})$ leading to the homogeneous system of SDEs (4), but with $\bar{F}(v,w)$ instead of $F(v,w,t/\epsilon_{2})$. Then, an application of Theorem 2.1 on this system gives an averaged vector field

$${\bar{G}}_{\infty }(w):=\int_{v\in R^{p}}G(v,w){\bar{\rho }}^{w}(dv).$$

2. To study the extremal cases μ=0 and μ=∞ in full generality, one would need to consider all the possible relationships between $\epsilon_{1}$ and $\epsilon_{2}$, not only the linear one as in the present article, but also of the type $\epsilon_{1}=\epsilon_{2}^{\alpha }$ for example. In this case, we believe that the regime α<1 converges to the same limit as taking the limit $\epsilon_{2}$ first and the regime α>1 corresponds to taking the limit $\epsilon_{1}$ first. The intermediate regime α=1 seems to be the only one for which the limit cannot be obtained by combining classical averaging principles. Therefore, the present article is focused on this case, in which the averaged system depends explicitly on the scaling parameter *μ*. Moreover, in terms of applications, this parameter can have a relatively easy interpretation in terms of the ratio of time-scales between intrinsic neuronal activity and typical stimulus time-scales in a given situation. Although the zeroth order limit (*i.e.*, the averaged system) seems to depend only on the position of *α* with respect to 1, it seems reasonable to expect that the fluctuations around the limit would depend on the precise value of *α*. This is a difficult question which may deserve further analysis.

The case 0<μ<∞ is already very rich in the sense that it combines simultaneously both the periodic and stochastic averaging principles in a new way that cannot be recovered by sequential applications of those principles. A particular role is played by the frozen periodically-forced SDE (6). The equivalent of the quasi-stationary measure $\rho^{w}$ of Theorem 2.1 is given by the asymptotically periodic behavior of equation (6), represented by the periodic family of measures $\nu_{\mu }^{w}(t,dv)$.

3. By a rescaling of the frozen process (6), one deduces the following *scaling* relationships:

$$\nu_{\mu }^{w}[F,\Sigma ](t,dv)=\nu_{1}^{w}\left[\frac{F}{\mu },\frac{\Sigma }{\sqrt{\mu }}\right](\mu t,dv)$$

and

$${\bar{G}}_{\mu }^{\left[F,\Sigma \right]}(w)={\bar{G}}_{1}^{\left[\frac{F}{\mu },\frac{\Sigma }{\sqrt{\mu }}\right]}(w).$$

Therefore, if one knows, in the case μ=1, the averaged vector field associated with the fast process generated by a drift *F* and a diffusion coefficient *σ*, denoted ${\bar{G}}_{1}[F,\Sigma ]$, it is possible to deduce ${\bar{G}}_{\mu }$ in the general case μ∈(0,∞) with a change F→μF and $\Sigma \to \sqrt{\mu }\Sigma$.

4. It seems reasonable to expect that the above result is still valid when considering ergodic, but not necessarily periodic, time dependency of the function F(v,w,⋅). In equation (7), instead of integrating $\nu_{\mu }^{w}(t,dv)$ over one period, one should integrate it with respect to an ergodic stationary measure. However, this extension requires non-trivial technical improvements of [5] which are beyond the scope of this paper. 

#### 2.3.1 Case of a fast linear SDE with periodic input

We present here an elementary case where one can compute explicitly the quasi-stationary time-periodic family of measures $\nu_{\mu }^{w}(t,x)$, when the equation for the fast variable is linear. Namely, we consider $v\in R^{p}$ the solution of

dv(t)=(−A⋅v(t)+u(μt))dt+Σ⋅dB(t),

with initial condition $v(0)=v_{0}\in R^{p}$, and where $A\in R^{p\times p}$ is a matrix whose eigenvalues have positive real parts and u(⋅) is a *τ*-periodic function.

We are interested in the large time behavior of the law of v(t), which is a time-inhomogeneous Ornstein-Uhlenbeck process. From [5] we know that its law converges to a *τ*-periodic family of probability measures ν(t,dv). Due to the linearity in the previous equation, ν(t,dv) is Gaussian with a time-dependent mean and a constant covariance matrix

$$\nu (t,dv)=N_{\bar{v}(t),Q}(dv),$$

where $\bar{v}$ is the $\frac{\tau }{\mu }$-periodic attractor of $\frac{d\bar{v}}{dt}=-A\cdot \bar{v}(t)+u(\mu t)$, *i.e.*,

$$\bar{v}(t)=\int_{-\infty }^{t}e^{-A(t-s)}u(\mu s)\;ds,$$

and **Q** is the unique solution of the Lyapunov equation

(9) $$A\cdot Q+Q\cdot A^{\prime }+\Sigma \cdot \Sigma^{\prime }=0.$$

Indeed, if one denotes $c(t)=v(t)-\bar{v}(t)$, then c(t) is a solution of the classical homogeneous Ornstein-Uhlenbeck equation

dc(t)=−Ac(t)dt+ΣdB(t),

whose stationary distribution is known to be a centered Gaussian measure with the covariance matrix **Q** solution of (9); see Chapter 3.2 of [24]. Notice that if **A** is self-adjoint with respect to ${\left(\Sigma \cdot \Sigma^{\prime }\right)}^{-1}$ (*i.e.*, $A\cdot (\Sigma \cdot \Sigma^{\prime })=(\Sigma \cdot \Sigma^{\prime })\cdot A^{\prime }$), then the solution is $Q=\frac{A^{-1}\cdot (\Sigma \cdot \Sigma^{\prime })}{2}=\frac{(\Sigma \cdot \Sigma^{\prime })\cdot {A^{\prime }}^{-1}}{2}$, which will be used in Appendix B.2.

Hence, in the linear case, the averaged vector field of equation (7) becomes

(10) $${\bar{G}}_{\mu }(w):={\left(\frac{\tau }{\mu }\right)}^{-1}\int_{0}^{\frac{\tau }{\mu }}\int_{v\in R^{p}}G\left(\bar{v}(t)+v,w\right)N_{0,Q}(dv)\;dt,$$

where $N_{x,Q}$ is the probability density function of the Gaussian law with mean $x\in R^{q}$ and covariance $Q\in R^{p\times p}$.

Therefore, due to the linearity of the fast SDE, the periodic system of measure *ν* is just a constant Gaussian distribution shifted by a periodic function of time v(t). In case *G* is quadratic in **v**, this remark implies that one can perform independently the integral over time and over $R^{p}$ in formula (10) (noting that the crossed term has a zero average). In this case, contributions from the periodic input and from noise appear in the averaged vector field in an additive way.

*Example 2.3* In this last example, we consider a combination between Example 2.1 and Example 2.2, namely we consider the following system of periodically forced SDEs:

$$\begin{array}{rcl} dv^{\epsilon } & = & \frac{1}{\epsilon_{1}}\left[-v^{\epsilon }+\sin\left(\frac{t}{\epsilon_{2}}\right)\right]\;dt+\frac{\sigma }{\sqrt{\epsilon_{1}}}\;dB(t),\\ dw^{\epsilon } & = & \left(-w^{\epsilon }+{\left(v^{\epsilon }\right)}^{2}\right)\;dt. \end{array}$$

As in Example 2.1 and as shown above, the behavior of this system when both $\epsilon_{1}$ and $\epsilon_{2}$ are small depends on the parameter *μ* defined in (5). More precisely, we have the following three regimes: 

• Regime 1: slow input:

$${\bar{G}}_{0}(w)=-w+\frac{\sigma^{2}}{2}+\frac{1}{2}.$$

• Regime 2: fast input:

$${\bar{G}}_{\infty }(w)=-w+\frac{\sigma^{2}}{2}.$$

• Regime 3: time-scale matching:

$${\bar{G}}_{\mu }(w)=-w+\frac{\sigma^{2}}{2}+\frac{1}{2(1+\mu^{2})}.$$

### 2.4 Truncation and asymptotic well-posedness

In some cases, Assumptions 2.1-2.2 may not be satisfied on the entire phase space $R^{p}\times R^{q}$, but only on a subset. Such situations will appear in Section 3 when considering learning models. We introduce here a more refined set of assumptions ensuring that Theorem 2.2 still applies.

Let us start with an example, namely the following bi-dimensional system with white noise input:

(11) $$\left\{ \begin{array}{c} dv^{\epsilon }=\frac{1}{\epsilon }(-lv^{\epsilon }+w^{\epsilon }v^{\epsilon })\;dt+\frac{\sigma }{\sqrt{\epsilon }}\;dB(t),\\ dw^{\epsilon }=(-\kappa w^{\epsilon }+{\left(v^{\epsilon }\right)}^{2})\;dt, \end{array}\right.$$

with ϵ>0, σ>0, l>0, μ>0.

For the fast drift −(l−w)v to be non-explosive, it is necessary to have w<l−α with α>0 for all time. The concern about this system comes from the fact that the slow variable *w* may reach *l* due to the fluctuations captured in the term $v^{2}$, for instance, if *κ* is not large enough. Such a system may have exponentially growing trajectories. However, we claim that for small enough *ϵ*, $w^{\epsilon }$ will remain close to its averaged limit *w* for a very long time, and if this limit remains below l−α, then $w^{\epsilon }$ can be considered as well-posed in the asymptotic limit ϵ→0. To make this argument more rigorous, we suggest the following definition.

**Definition 2.3** A stochastic differential equation with a given initial condition is asymptotically well posed in probability if for the given initial condition, 

1. a unique solution exists until a random time $\tau_{\epsilon }$

2. for all T>0,

$$\lim_{\epsilon \to 0}P[\tau_{\epsilon }\geq T]=1.$$

We give in the following proposition sufficient conditions for system (4) to be asymptotically well posed in probability and to satisfy conclusions of Theorem 2.2.

Let us introduce the following set of additional assumptions.

**Assumption 2.3** Moment conditions:

(i) There exists p>2 such that

$$\text{for any }T>0,\;\underset{\epsilon }{\sup}E\left[\underset{0\leq t\leq T}{\sup}{∥v_{t}^{\epsilon }∥}^{p}+{∥w_{t}^{\epsilon }∥}^{p}\right]<\infty .$$

(ii) For any T>0 and any bounded subset *K* of $R^{q}$,

$$\underset{\epsilon_{1}>0,\epsilon 2>0,w\in K}{\sup}E\left[\underset{0\leq t\leq T}{\sup}{∥G\left(v_{t}^{\epsilon },w\right)∥}^{2}\right]<\infty .$$

*Remark 2.2* This last set of assumptions will be satisfied in all the applications of Section 3 since we consider linear models with additive noise for the equation of **v**, implying this variable to be Gaussian and the function *G* only involves quadratic moments of **v**; therefore, the moment conditions (i) and (ii) will be satisfied without any difficulty. Moreover, if one considers non-linear models for the variable **v**, then the Gaussian property may be lost; however, adding sigmoidal non-linearity has, in general, the effect of bounding the dynamics, thus making these moment assumptions reasonable to check in most models of interest.

**Property 2.3***If there exists a subset* ℰ *of*$R^{q}$*such that*

1. *The functionsF*, *G*, **Σ***satisfy Assumptions *2.1-2.3 *restricted on*$R^{p}\times E$.

2. ℰ *is invariant under the flow of*${\bar{G}}_{\mu }$, *as defined in* (7).

*Then for any initial condition*$w_{0}\in E$, *system* (4) *is asymptotically well posed in probability and*$w^{\epsilon }$*satisfies the conclusion of Theorem *2.2.

*Proof* See Appendix A.2. □

Here, we show that it applies to system (11). First, with $E_{\alpha }=\{w\in R,w<l-\alpha \}$, for some α∈]0,l[, it is possible to show that Assumptions 2.1-2.2 are satisfied on $R^{p}\times E_{\alpha }$. Then, as a special case of (10), we obtain the following averaged system:

$$\frac{dw}{dt}=-\kappa w+\frac{\sigma^{2}}{2(l-w)}:=\bar{G}(w).$$

It remains to check that the solution of this system satisfies

∃α>0,such that w(0)<l−α⇒∀t>0,w(t)<l−α,

that is, the subset $E_{\alpha }$ is invariant under the flow of $\bar{G}$.

This property is satisfied as soon as

$$\eta :=\frac{2\sigma^{2}}{\kappa l^{2}}<1.$$

Indeed, one can show that $\bar{G}(w)=0$ admits two solutions iff η<1,

$$w_{\pm }=\frac{l}{2}(1\pm \sqrt{1-\eta })\in (0,l),$$

and that $w_{-}$ is stable whereas $w_{+}$ is unstable. Thus, if w(0)<l−α with $\alpha =l-w_{+}>0$, then w(t)<l−α for all t>0. In fact, the invariance property is true for all $\alpha \in \;]l-w_{-},l-w_{+}[$.

## 3 Averaging learning neural networks

In this section, we apply the temporal averaging methods derived in Section 2 on models of unsupervised learning neural networks. First, we design a generic learning model and show that one can define formally an averaged system with equation (7). However, going beyond the mere definition of the averaged system seems very difficult and we only manage to get explicit results for simple systems where the fast activity dynamics is linear. In the three last subsections, we push the analysis for three examples of increasing complexity.

In the following, we always consider that the initial connectivity is 0. This is an arbitrary choice but without consequences, because we focus on the regime where there is a single globally stable equilibrium point (see Section 3.2.3).

### 3.1 A generic learning neural network

We now introduce a large class of stochastic neuronal networks with learning models. They are defined as coupled systems describing the simultaneous evolution of the activity of n∈N neurons and the connectivity between them. We define $v\in R^{n}$, the *activity field* of the network, and $W\in R^{n\times n}$, the *connectivity matrix*.

Each neuron variable $v_{i}$ is assumed to follow the SDE

$$dv_{i}=\left(f_{i}(v_{i})+u_{i}\right)\;dt+\Sigma \cdot dB_{i}(t),$$

where the function $f_{i}$ characterizes the intrinsic non-linear dynamical behavior of neuron *i* and $u_{i}$ is the input received by neuron *i*. The stochastic term $\Sigma \cdot dB_{i}(t)$ is added to account for internal sources of noise. In terms of notations, ${\left(B(t)\right)}_{t\geq 0}$ is a standard *n*-dimensional Brownian motion, **Σ** is an n×n matrix, possibly function of **v** or other variables, and $\Sigma \cdot dB_{i}(t)$ denotes the *i*th component of the vector Σ⋅dB(t).

The input $u_{i}$ to neuron *i* has mainly two components: the external input $u_{i}^{\mathrm{ext}}$ and the input coming from other neurons in the network $u_{i}^{\mathrm{syn}}$. The latter is *a priori* a complex combination of post-synaptic potentials coming from many other neurons. The coefficient $W_{ij}$ of the connectivity matrix accounts for the strength of a synapse j→i. Note that neurons can be connected to themselves, *i.e.*, $W_{ii}$ is not necessarily null. Thus, we can write

$$u_{i}^{\mathrm{syn}}:=S\left(\sum_{j=1}^{n}W_{ij}H(v_{i},v_{j})\right),$$

where S:R→R and ℋ is a function taking the history of $v_{i}$ and $v_{j}$ and returning a real for each time *t* (to take convolutions into account). In practical cases, they are often taken to be sigmoidal functions. We abusively redefine S and ℋ as vector valued operators corresponding to the element-wise application of their real counterparts. We also define the function $F:R^{n}\to R^{n}$ such that $F{\left(v\right)}_{i}=f_{i}(v_{i})$. Together with a slow generic learning rule, this leads to defining a *stochastic learning model* as the following system of SDEs.

**Definition 3.1**

$$\left\{ \begin{array}{c} dv^{\epsilon }=\frac{1}{\epsilon }[F(v^{\epsilon })+S(W^{\epsilon }\cdot H(v^{\epsilon }))+u^{\mathrm{ext}}(t)]\;dt+\frac{1}{\sqrt{\epsilon }}\Sigma (v^{\epsilon },W^{\epsilon })\cdot dB(t),\\ dW^{\epsilon }=G(W^{\epsilon },v^{\epsilon })\;dt. \end{array}\right.$$

Before applying the general theory of Section 2, let us make several comments about this generic model of neural network with learning. This model is a non-autonomous, stochastic, non-linear slow-fast system.

In order to apply Theorem 2.2, one needs Assumptions 2.1, 2.2, and 2.3 to be satisfied, restricting the space of possible functions S, ℋ, ℱ, **Σ**, and *G*. In particular, Assumption 2.2(ii) seems rather restrictive since it excludes systems with multiple equilibria and suggests that the general theory is more suited to deal with rate-based networks. However, one should keep in mind that these assumptions are only sufficient, and that the double averaging principle may work as well in systems which do not satisfy readily those assumptions.

As we will show in Section 3.3, a particular form of history-dependence can be taken into account, to a certain extent. Indeed, for instance, if the function ℱ is actually a functional of the past trajectory of variable $v^{\epsilon }$ which can be expressed as the solution of an additional SDE, then it may be possible to include a certain form of history-dependence. However, purely time-delayed systems do not enter the scope of this theory, although it might be possible to derive an analogous averaging method in this framework.

The noise term can be purely additive or set by a particular function Σ(v,W) as long as it satisfies Assumption 2.2(i), meaning that it must be uniformly non-degenerate.

In the following subsection, we apply the averaging theory to various combinations of neuronal network models, embodied by choices of functions S, ℋ, ℱ, **Σ**, and various learning rules, embodied by a choice of the function *G*. We will also analyze the obtained averaged system, describing the slow dynamics of the connectivity matrix in the limit of perfect time-scale separation and, in particular, study the convergence of this averaged system to an equilibrium point.

### 3.2 Symmetric Hebbian learning

One of the simplest, yet non-trivial, stochastic learning models is obtained when considering 

• A linear model for neuronal activity, namely $f_{i}(v_{i})=-lv_{i}$ with *l* a positive constant.

• A linear model for the synaptic transmission, namely $S(v_{i})=v_{i}$ and $H(v_{i},v_{j})=v_{j}$.

• A constant diffusion matrix **Σ** (additive noise) proportional to the identity Σ=σId (spatially uncorrelated noise).

• A Hebbian learning rule with linear decay, namely $G_{ij}(W,v)=-\kappa W_{ij}+v_{i}v_{j}$. Actually, it corresponds to the tensor product: ${\left\{v⊗v\right\}}_{ij}=v_{i}v_{j}$.

 This model can be written as follows:

(12) $$\left\{ \begin{array}{c} dv^{\epsilon }=\frac{1}{\epsilon_{1}}(-L\cdot v^{\epsilon }+W^{\epsilon }\cdot v^{\epsilon }+u(\frac{t}{\epsilon_{2}}))\;dt+\frac{\sigma }{\sqrt{\epsilon_{1}}}\;dB(t),\\ \frac{dW^{\epsilon }}{dt}=G(v^{\epsilon },W^{\epsilon })=-\kappa W^{\epsilon }+v^{\epsilon }⊗v^{\epsilon }, \end{array}\right.$$

where neurons are assumed to have the same decay constant: $L=lI_{d}$; **u** is a periodic continuous input (it replaces $u^{\mathrm{ext}}$ in the previous section); $\sigma ,\epsilon_{1},\epsilon_{2},\kappa \in R_{+}$ with $\epsilon_{1},\epsilon_{2}\ll 1$ and B(t) is *n*-dimensional Brownian noise.

The first question that arises is about the well-posedness of the system: What is the definition interval of the solutions of system (12)? Do they explode in finite time? At first sight, it seems there may be a runaway of the solution if the largest real part among the eigenvalues of **W** grows bigger than *l*. In fact, it turns out this scenario can be avoided if the following assumption linking the parameters of the system is satisfied.

**Assumption 3.1** There exists p∈]0,1[ such that

$$\left(\frac{\sigma^{2}l}{2p(1-p)}+\frac{u_{m}^{2}}{p{\left(1-p\right)}^{2}}\right)<\kappa l^{3},$$

where $u_{m}=\sup_{t\in R_{+}}{∥u(t)∥}_{2}$.

It corresponds to making sure the external (*i.e.*, $u_{m}$) or internal (*i.e.*, *σ*) excitations are not too large compared to the decay mechanism (represented by *κ* and *l*). Note that if p∈]0,1[, $u_{m}$ and *d* are fixed, it is sufficient to increase *κ* or *l* for this assumption to be satisfied.

Under this assumption, the space

$$E_{p}=\left\{W\in R^{n\times n}:W\text{ is symmetric},W\geq 0\text{ and }W<pL\right\}$$

is invariant by the flow of the averaged system $\bar{G}$, where W≥0 means **W** is semi-definite positive and W<pL means pL−W is definite positive. Therefore, the averaged system is defined and bounded on $R_{+}$. The slow/fast system being asymptotically close to the averaged system, it is therefore asymptotically well-defined in probability. This is summarized in the following theorem.

**Theorem 3.1***If Assumption *3.1 *is verified for*p∈]0,1[, *then system* (12) *is asymptotically well posed in probability and the connectivity matrix*$W^{\epsilon }$, *the solution of system* (12), *converges to***W***in the sense that for all*δ,T>0,

$$\lim_{\epsilon \to 0}^{\mu }P\left[\underset{t\in [0,T]}{\sup}{\left|W_{t}^{\epsilon }-W_{t}\right|}^{2}>\delta \right]=0,$$

*where***W***is the deterministic solution of*

(13) $$\frac{dW_{ij}}{dt}=\bar{G}{\left(W\right)}_{ij}=\underset{\mathrm{decay}}{\underset{︸}{-\kappa W_{ij}}}+\frac{\mu }{\tau }\underset{\mathrm{correlation}}{\underset{︸}{\int_{0}^{\frac{\tau }{\mu }}{\bar{v}}_{i}(s){\bar{v}}_{j}(s)\;ds}}+\underset{\mathrm{noise}}{\underset{︸}{\frac{\sigma^{2}}{2}{\left(L-W\right)}_{ij}^{-1}}},$$

*where*$\bar{v}(t)$*is the*$\frac{\tau }{\mu }$-*periodic attractor of*$\frac{d\bar{v}}{dt}=(W-L)\cdot \bar{v}+u(\mu t)$, *where*$W\in R^{n\times n}$*is supposed to be fixed*.

*Proof* See Theorem B.1 in Appendix B.2. □

In the following, we focus on the averaged system described by (13). Its right-hand side is made of three terms: a linear and homogeneous decay, a correlation term, and a noise term. The last two terms are made explicit in the following.

#### 3.2.1 Noise term

As seen in Section 2, in the linear case, the noise term **Q** is the unique solution of the Lyapunov equation (9) with A=W−L and Σ=σId. Because the noise is spatially uncorrelated and identical for each neuron and also because the connectivity is symmetric, observe that $Q=\frac{\sigma^{2}}{2}{\left(L-W\right)}^{-1}$ is the unique solution of the system.

In more complicated cases, the computation of this term appears to be much more difficult as we will see in Section 3.4.

#### 3.2.2 Correlation term

This term corresponds to the auto-correlation of neuronal activity. It is only implicitly defined; thus, this section is devoted to finding an explicit form depending only on the parameters *l*, *μ*, *τ*, the connectivity **W**, and the inputs **u**. Actually, one can perform an expansion of this term with respect to a small parameter corresponding to a *weakly connected expansion*. Most terms vanish if the connectivity **W** is small compared to the strength of the intrinsic decaying dynamics of neurons *l*.

The auto-correlation term of a $\frac{\tau }{\mu }$-periodic function can be rewritten as

$${\left\{\bar{v}\cdot {\bar{v}}^{\prime }\right\}}_{ij}=\int_{0}^{\frac{\tau }{\mu }}{\bar{v}}_{i}(s){\bar{v}}_{j}(s)\;ds.$$

With this notation, it is simple to think of **v** as a ‘semi-continuous matrix’ of $R^{n\times [0,\frac{\tau }{\mu }[}$. Hence, the operator ‘⋅’ can be though of as a matrix multiplication. Similarly, the transpose operator turns a matrix $\bar{v}\in R^{n\times [0,\frac{\tau }{\mu }[}$ into a matrix ${\bar{v}}^{\prime }\in R^{[0,\frac{\tau }{\mu }[\times n}$. See Appendix B.1 for details about the notations.

 It is common knowledge, see [17] for instance, that this term gathers information about the correlation of the inputs. Indeed, if we assume that the input is sufficiently slow, then $\bar{v}$ has enough time to converge to u(t) for all t∈[0,+∞[. Therefore, in the first order $\bar{v}(t)≃{\left(W-L\right)}^{-1}\cdot u(t)$. This leads to $\bar{v}\cdot {\bar{v}}^{\prime }≃{\left(W-L\right)}^{-1}\cdot u\cdot u^{\prime }\cdot {\left(W^{\prime }-L\right)}^{-1}$. In the weakly connected regime, one can assume that W−L≃−L leading to $\bar{v}\cdot {\bar{v}}^{\prime }≃\frac{1}{l^{2}}u\cdot u^{\prime }$ which is the auto-correlation of the inputs.

Actually, without the assumption of a slow input, lagged correlations of the input appear in the averaged system. Before giving the expression of these temporal correlations, we need to introduce some notations. First, define the convolution filter $g_{l/\mu }:t\mapsto \frac{l}{\mu }e^{-\frac{l}{\mu }t}H(t)$, where *H* is the Heaviside function. This family of functions is displayed for different values of $\frac{l}{\mu }$ in Figure 4(a). Note that $g_{l/\mu }\to \delta_{0}$ when $\frac{l}{\mu }\to +\infty$, where $\delta_{0}$ is the Dirac distribution centered at the origin. In this asymptotic regime, the convolution filter and its iterates $g_{l/\mu }*\cdots *g_{l/\mu }$ are equal to the identity.

**Fig. 1:**
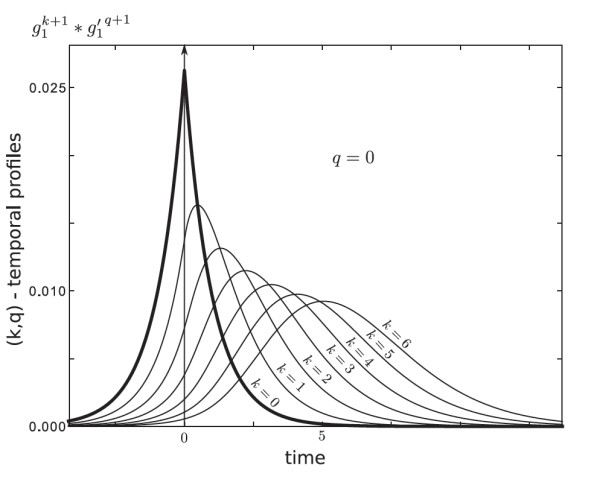
This shows the (k,q)-temporal profiles with $\frac{l}{\mu }=1$, *i.e.*, the functions $g_{1}^{\left(k+1\right)}*{g_{1}^{\prime }}^{\left(q+1\right)}$ for q=0 and *k* ranging from 0 to 6. For k=q=0, the temporal profile is even and this also occurs to be true for any k=q. When k>q, the function reaches its maximum for strictly positive values that grow with the difference k−q. Besides, the temporal profiles are flattened when k+q increases.

We also define the filtered correlation of the inputs $C^{k,p}\in R^{n\times n}$ by

$$C^{k,q}\overset{\mathrm{def}}{=}\frac{1}{u_{m}^{2}\tau }\left(u*g_{l/\mu }^{\left(k+1\right)}\right)\cdot {\left(u*g_{l/\mu }^{\left(q+1\right)}\right)}^{\prime },$$

where $g_{l/\mu }^{\left(k+1\right)}=g_{l/\mu }*\cdots *g_{l/\mu }$ is the *k*th convolution of $g_{l/\mu }$ with itself and $u_{m}=\sup_{t\in R_{+}}{∥u(t)∥}_{2}$. This is the correlation matrix of the inputs filtered by two different functions. It is easy to show that this is similar to computing the cross-correlation of the inputs with the inputs filtered by another function,

(14) $$\begin{array}{rcl} C^{k,q} & = & \frac{1}{u_{m}^{2}\tau }\left(u*\left(g_{l/\mu }^{\left(k+1\right)}*{g_{l/\mu }^{\left(q+1\right)}}^{\prime }\right)\right)\cdot u^{\prime }\\ & = & \frac{1}{u_{m}^{2}\tau }u\cdot {\left(u*\left(g_{l/\mu }^{\left(k+1\right)}*{g_{l/\mu }^{\left(q+1\right)}}^{\prime }\right)\right)}^{\prime }, \end{array}$$

which motivates the definition of the (k,p)-temporal profile $g_{l/\mu }^{\left(k+1\right)}*{g_{l/\mu }^{\prime }}^{\left(q+1\right)}$, where ${\left(g_{l/\mu }^{\prime }\right)}^{\left(k\right)}(t)={\left(g_{l/\mu }^{\left(k\right)}\right)}^{\prime }(t)=g_{l/\mu }^{\left(k\right)}(-t)$. This notation is deliberately similar to that of the transpose operator we use in the proofs. These functions are shown in Figure 1. We have not found a way to make them explicit; therefore, the following remarks are simply based on numerical illustrations. When k=q, the temporal profiles are centered. The larger the difference k−q, the larger the center of the bell. The larger the sum k+q, the larger the standard deviation. This motivates the idea that $C^{k,p}$ can be thought of as the k−q lagged correlation of the inputs. One can also say that $C^{10,10}$ is more blurred than $C^{0,0}$ in the sense that the inputs are temporally integrated over a ‘wider’ window in the first case. 

Observe that $g_{l/\mu }^{\left(k+1\right)}(t)=\frac{l^{k+1}}{\mu^{k+1}k!}t^{k}e^{-\frac{l}{\mu }t}H(t)$. Therefore, ${∥g_{l/\mu }^{\left(k+1\right)}∥}_{1}=\frac{\Gamma (k+1)}{k!}=1$. Thanks to Young’s inequality for convolutions, which says that ${∥u*g_{l/\mu }^{\left(k\right)}∥}_{2}\leq {∥u∥}_{2}{∥g_{l/\mu }^{\left(k\right)}∥}_{1}$, it can be proved that ${∥C^{k,q}∥}_{2}\leq 1$.

 We intend to express the correlation term as an infinite converging sum involving these filtered correlations. In this perspective, we use a result we have proved in [25] to write the solution of a general class of non-autonomous linear systems (*e.g.*, $\frac{d\bar{v}}{dt}=(W-L)\cdot \bar{v}+u(t)$) as an infinite sum, in the case μ=1.

**Lemma 3.2***If*$\bar{v}$*is the solution*, *with zero as initial condition*, *of*$\frac{d\bar{v}}{dt}=(W-L)\cdot \bar{v}+u(t)$*it can be written by the sum below which converges if***W***is in*$E_{p}$*for*p∈]0,1[.

$$\bar{v}=\sum_{k=0}^{+\infty }\frac{W^{k}}{l^{k+1}}\cdot u*g_{l}^{\left(k+1\right)},$$

*where*$g_{l}:t\mapsto le^{-lt}H(t)$.

*Proof* See Lemma B.2 in Appendix B.2. □

This is a decomposition of the solution of a linear differential system on the basis of operators where the spatial and temporal parts are decoupled. This important step in a detailed study of the averaged equation cannot be achieved easily in models with non-linear activity. Everything is now set up to introduce the explicit expansion of the correlation we are using in what follows. Indeed, we use the previous result to rewrite the correlation term as follows.

**Property 3.3***The correlation term can be written*

$$\frac{\mu }{\tau }\bar{v}\cdot {\bar{v}}^{\prime }=\frac{u_{m}^{2}}{l^{2}}\sum_{k,q=0}^{+\infty }\frac{W^{k}}{l^{k}}\cdot C^{k,q}\cdot \frac{{W^{\prime }}^{q}}{l^{q}}.$$

*Proof* See Theorem B.3 in Appendix B.2. □

 This infinite sum of convolved filters is reminiscent of a property of Hawkes processes that have a linear input-output gain [26]. 

The speed of inputs characterized by *μ* only appears in the temporal profiles $g_{l/\mu }^{\left(k\right)}*{g_{l/\mu }^{\prime }}^{\left(q\right)}$. In particular, if the inputs are much slower than neuronal activity time-scale, *i.e.*, μ=0, then $g_{+\infty }=\delta_{0}$ and $u*g_{+\infty }=u$. Therefore, $C^{k,q}=C^{0,0}$ and the sums in the formula of Property 3.3 are separable, leading to $\bar{v}\cdot {\bar{v}}^{\prime }={\left(L-W\right)}^{-1}\cdot u\cdot u^{\prime }\cdot {\left(L-W^{\prime }\right)}^{-1}$, which corresponds to the heuristic result previously explained.

Therefore, the averaged equation can be explicitly rewritten

(15) $$\frac{dW}{dt}=\bar{G}(W)=-\kappa W+\frac{u_{m}^{2}}{l^{2}}\sum_{k,q=0}^{+\infty }\frac{W^{k}}{l^{k}}\cdot C^{k,q}\cdot \frac{{W^{\prime }}^{q}}{l^{q}}+\frac{\sigma^{2}}{2}{\left(L-W\right)}^{-1}.$$

**Fig. 2:**
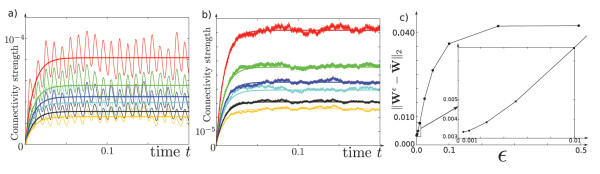
The first two figures, (**a**) and (**b**), represent the evolution of the connectivity for original stochastic system (12), superimposed with averaged system (13), for two different values of *ϵ*: respectively ϵ=0.01 and ϵ=0.001, where we have chosen $\epsilon =\epsilon_{1}=\epsilon_{2}$. Each color corresponds to the weight of an edge in a network made of n=3 neurons. As expected, it seems that the smaller *ϵ*, the better the approximation. This can be seen in the picture (**c**) where we have plotted the precision on the *y*-axis and *ϵ* on the *x*-axis. The parameters used here are l=12, μ=1, κ=100, σ=0.05. The inputs have a random (but frozen) spatial structure and evolve according to a sinusoidal function.

In Figure 2, we illustrate this result by comparing, for different $\epsilon =\epsilon_{1}=\epsilon_{2}$ (*i.e.*, we choose μ=1 in this example), the stochastic system and its averaged version. The above decomposition has been used as the basis for numerical computation of trajectories of the averaged system. 

#### 3.2.3 Global stability of the equilibrium point

Now that we have found an explicit formulation for the averaged system, it is natural to study its dynamics. Actually, we prove in the following that if the connectivity **W** is kept smaller than $\frac{l}{3}$, *i.e.*, Assumption 3.1 is verified with $p\leq \frac{1}{3}$, then the dynamics is trivial: the system converges to a single equilibrium point. Indeed, under the previous assumption, the system can be written $\bar{G}(W)=-\kappa W+F(W)$, where *F* is a contraction operator on $E_{\frac{1}{3}}$. Therefore, one can prove the uniqueness of the fixed point with the Banach fixed point argument and exhibit an energy function for the system.

**Theorem 3.4***If Assumption *3.1 *is verified for*$p\leq \frac{1}{3}$, *then there is a unique equilibrium point in the invariant subset*$E_{p}$*which is globally*, *asymptotically stable*.

*Proof* See Theorem B.4 in Appendix B.2. □

The fact that the equilibrium point is unique means that the ‘knowledge’ of the network about its environment (corresponding by hypothesis to the connectivity) eventually is unique. For a given input and any initial condition, the network can only converge to the same ‘knowledge’ or ‘understanding’ of this input.

#### 3.2.4 Explicit expansion of the equilibrium point

When the network is weakly connected, the high-order terms in expansion (15) may be neglected. In this section, we follow this idea and find an explicit expansion for the equilibrium connectivity where the strength of the connectivity is the small parameter enabling the expansion. The weaker the connectivity, the more terms can be neglected in the expansion.

In fact, it is not natural to speak about a weakly connected learning network since the connectivity is a variable. However, we are able to identify a *weak connectivity index* which controls the strength of the connectivity. We say the connectivity is weak when it is negligible compared to the intrinsic leak term, *i.e.*, $\frac{⦀W⦀}{l}$ is small. We show in the Appendix that this weak connectivity index depends only on the parameters of the network and can be written

$$\tilde{p}=\frac{u_{m}^{2}}{\kappa l^{3}}+\frac{\sigma^{2}}{2\kappa l^{2}}.$$

In the asymptotic regime $\tilde{p}\to 0$, we have $\frac{W}{\tilde{p}l}=O(1)$. This index is the ‘small’ parameter needed to perform the expansion. We also define $\lambda =\frac{\sigma^{2}l}{2u_{m}^{2}}$, which has information about the way $\tilde{p}$ is converging to zero. In fact, it is the ratio of the two terms of $\tilde{p}$.

With these, we can prove that the equilibrium connectivity $W^{*}$ has the following asymptotic expansion in $\tilde{p}$.

**Theorem 3.5**

$$\begin{array}{rcl} W^{*} & = & \frac{\tilde{p}l}{1+\lambda }\left(\lambda +C^{0,0}\right)+\frac{{\tilde{p}}^{2}l}{{\left(1+\lambda \right)}^{2}}(\lambda^{2}+\lambda \left(C^{0,0}+C^{1,0}+C^{0,1}\right)\\ & & +C^{0,0}\cdot C^{1,0}+C^{0,1}\cdot C^{0,0})+O\left({\tilde{p}}^{3}\right). \end{array}$$

*Proof* See Theorem B.5 in Appendix B.2. □

**Fig. 3:**
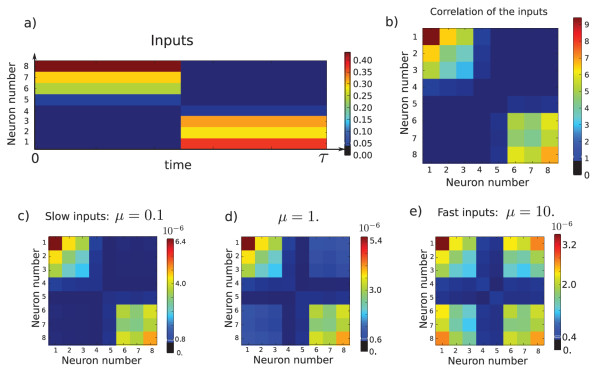
(**a**) shows the temporal evolution of the input to a n=8 neurons network. It is made of two spatially random patterns that are shown alternatively. (**b**) shows the correlation matrix of the inputs. The off-diagonal terms are null because the two patterns are spatially orthogonal. (**c**), (**d**), and (**e**) represent the first order of Theorem 3.5 expansion for different *μ*. Actually, this approximation is quite good since the percentage of error between the averaged system and the first order, computed by $\mathrm{error}=\frac{{∥W-\mathrm{order}\;1∥}_{1}}{{∥W∥}_{1}}$, have an order of magnitude of 10^−4^% for the three figures. These figures make it possible to observe the role of *μ*. If *μ* is small, *i.e.*, the inputs are slow, then the transient can be neglected and the learned connectivity is roughly the correlation of the inputs; see (a). If *μ* increases, *i.e.*, the inputs are faster, then the connectivity starts to encode a link between the two patterns that were flashed circularly and elicited responses that did not fade away when the other pattern appeared. The temporal structure of the inputs is also learned when *μ* is large. The parameters used in this figure are ϵ=0.001, l=12, κ=100, σ=0.02.

At the first order, the final connectivity is $C^{0,0}$, the filtered correlation of the inputs convolved with a bell-shaped centered temporal profile. In the case of Figure 3, this is quite a good approximation of the final connectivity. 

Not only the spatial correlation is encoded in the weights, but there is also some information about the temporal correlation, *i.e.*, two successive but orthogonal events occurring in the inputs will be wired in the connectivity although they do not appear in the spatial correlations; see Figure 3 for an example.

### 3.3 Trace learning: band-pass filter effect

In this section, we study an improvement of the learning model by adding a certain form of history dependence in the system and explain the way it changes the results of the previous section. Given that Theorem 2.2 only applies to an instantaneous process, we will only be able to treat the history-dependent systems which can be reformulated as instantaneous processes. Actually, this class of systems contains models which are biologically more relevant than the previous model and which will exhibit interesting additional functional behaviors. In particular, this covers the following features: 

• Trace learning.

 It is likely that a biological learning rule will integrate the activity over a short time. As Földiàk suggested in [27], it makes sense to consider the learning equation as being

$$\frac{dW^{\epsilon }}{dt}=-\kappa W^{\epsilon }+\left(v^{\epsilon }*g_{1}\right)⊗\left(v^{\epsilon }*g_{1}\right),$$

where ∗ is the convolution and $g_{1}:t\in R\mapsto \beta_{1}e^{-\beta_{1}t}H(t)$. Rolls and Deco numerically show [15] that the temporal convolution, leading to a spatio-temporal learning, makes it possible to perform invariant object recognition. Besides, trace learning appears to be the symmetric part of the biological STDP rule that we detail in Section 3.4.

• Damped oscillatory neurons.

Many neurons have an oscillatory behavior. Although we cannot take this into account in a linear model, we can model a neuron by a damped oscillator, which also introduces a new important time-scale in the system. Adding adaptation to neuronal dynamics is an elementary way to implement this idea. This corresponds to modeling a single neuron without inputs by the equivalent formulations

$$\left\{ \begin{array}{c} \frac{dv^{\epsilon }}{dt}=-lz^{\epsilon },\\ \frac{dz^{\epsilon }}{dt}=\beta_{2}(v^{\epsilon }-z^{\epsilon }) \end{array}\Leftrightarrow \;\{\begin{array}{c} \frac{dv^{\epsilon }}{dt}=-lv^{\epsilon }*g_{2},\\ \;\text{where }g_{2}(t)=\beta_{2}e^{-\beta_{2}t}H(t). \end{array}\right.$$

• Dynamic synapses.

The electro-chemical process of synaptic communication is very complicated and non-linear. Yet, one of the features of synaptic communication we can take into account in a linear model is the shape of the post-synaptic potentials. In this section, we consider that each synapse is a linear filter whose finite impulse response (*i.e.*, the post-synaptic potential) has the shape $g_{3}(t)=\beta_{3}e^{-\beta_{3}t}H(t)$. This is a common assumption which, for instance, is at the basis of traditional rate based models; see Chapter 11 of [7]. 

For mathematical tractability, we assume in the following that $\beta =\beta_{1}=\beta_{2}=\beta_{3}\in R_{+}$ such that $g_{\beta }=g_{1}=g_{2}=g_{3}$, *i.e.*, the time-scales of the neurons, those of the synapses and those of the learning windows are the same. Actually, there is a large variety of temporal scales of neurons, synapses, and learning windows, which makes this assumption not absurd. Besides, in many brain areas, examples of these time constants are in the same range (≃10 ms). Yet, investigating the impact of breaking this assumption would be necessary to model better biological networks. This leads to the following system:

(16) $$\left\{ \begin{array}{c} dv^{\epsilon }=\frac{1}{\epsilon_{1}}((W^{\epsilon }-L)\cdot v^{\epsilon }*g_{\beta }+u(\frac{t}{\epsilon_{2}}))\;dt+\frac{\sigma }{\sqrt{\epsilon_{1}}}\;dB(t),\\ \frac{dW^{\epsilon }}{dt}=-\kappa W^{\epsilon }+(v^{\epsilon }*g_{\beta })⊗(v^{\epsilon }*g_{\beta }), \end{array}\right.$$

where the notations are the same as in Section 3.2. The behavior of a single neuron will be oscillatory damped if $\Delta =\sqrt{1-4\frac{l}{\beta }}$ is a pure imaginary number, *i.e.*, 4l>β. This is the regime on which we focus. Actually, the Hebbian linear case of Section 3.2 corresponds to β=+∞ in this delayed system.

To comply with the hypotheses of Theorem 2.2 (*i.e.*, no dependence of the history of the process), we can add a variable **z** to the system which takes care of integrating the variable **v** over an exponential window. It leads to the equivalent system (in the limit $\sigma_{z}\to 0$)

$$\left\{ \begin{array}{c} d\left(\begin{array}{c} v^{\epsilon }\\ z^{\epsilon } \end{array}\right)=\frac{1}{\epsilon_{1}}\left[\left(\begin{array}{cc} 0 & W-L\\ \beta & -\beta \end{array}\right)\left(\begin{array}{c} v^{\epsilon }\\ z^{\epsilon } \end{array}\right)+\left(\begin{array}{c} u(\frac{t}{\epsilon_{2}})\\ 0 \end{array}\right)\right]\;dt+\left(\begin{array}{c} \frac{\sigma }{\sqrt{\epsilon_{1}}}\;dB(t)\\ \frac{\sigma_{z}}{\sqrt{\epsilon_{1}}}\;dB(t) \end{array}\right),\\ \frac{dW^{\epsilon }}{dt}=-\kappa W^{\epsilon }+z^{\epsilon }⊗z^{\epsilon }. \end{array}\right.$$

This trick makes it possible to deal with some history-based processes where the dependence on the past is exponential.

It turns out most of the results of Section 3.2 remain true for system (16) as detailed in the following. The existence of the solution on $R_{+}$ is proved in Theorem B.6. The computations show that in the averaged system, the noise term remains identical, whereas the correlation term is to be replaced by $\frac{\mu }{\tau }(\bar{v}*g_{\beta })\cdot {\left(\bar{v}*g_{\beta }\right)}^{\prime }$. Besides, Lemma 3.2 can be extended to our delayed system by changing only the temporal filters; see Lemma 34. Together with Lemma C.3, this proves the result of Theorem B.8.

$$\frac{\mu }{\tau }(\bar{v}*g_{\beta })\cdot {\left(\bar{v}*g_{\beta }\right)}^{\prime }=\frac{u_{m}^{2}{∥v∥}_{1}^{2}}{l^{2}}\sum_{k,q=0}^{+\infty }\frac{W^{k}}{{\left(l/{∥v∥}_{1}\right)}^{k}}\cdot {\tilde{C}}^{k,q}\cdot \frac{{W^{\prime }}^{q}}{{\left(l/{∥v∥}_{1}\right)}^{q}},$$

where

$${\tilde{C}}^{k,q}=\frac{1}{u_{m}^{2}\tau {∥v∥}_{1}^{k+q+2}}\left(u*v^{\left(k+1\right)}\right)\cdot {\left(u*v^{\left(q+1\right)}\right)}^{\prime },$$

where $v:t\to \frac{l}{\mu \Delta }(e^{-\frac{\beta }{2\mu }(1-\Delta )t}-e^{-\frac{\beta }{2\mu }(1+\Delta )t})H(t)$. Observe that applying Young’s inequality to convolutions leads to ${∥{\tilde{C}}^{k,q}∥}_{2}\leq 1$. Actually, Lemma C.3 shows that $v^{\left(k\right)}=v_{k}:t\mapsto \frac{\sqrt{\pi \beta }}{k!}e^{-\frac{\beta }{2}t}{\left(\frac{t}{\left|\Delta \right|}\right)}^{k+\frac{1}{2}}J_{k+\frac{1}{2}}(\frac{\beta |\Delta |}{2}t)H(t)$, where $J_{n}(z)$ is the Bessel function of the first kind. The value of the L1 norm of *v* is computed in Appendix C.3. It leads to ${∥v∥}_{1}=\coth(\frac{\pi }{2\Delta })$ if Δ is a pure imaginary number and ${∥v∥}_{1}=1$ else.

Therefore, the averaged system can be rewritten

$$\frac{dW}{dt}=\bar{G}(W)=-\kappa W+\frac{u_{m}^{2}{∥v∥}_{1}^{2}}{l^{2}}\sum_{k,q=0}^{+\infty }\frac{W^{k}}{{\left(l/{∥v∥}_{1}\right)}^{k}}\cdot {\tilde{C}}^{k,q}\cdot \frac{{W^{\prime }}^{q}}{{\left(l/{∥v∥}_{1}\right)}^{q}}+\frac{\sigma^{2}}{2}{\left(L-W\right)}^{-1}.$$

As before, the existence and uniqueness of a globally attractive equilibrium point is guaranteed if Assumption 3.1 is verified for $p\leq \frac{1}{2{∥v∥}_{1}^{3}+1}$; see Theorem B.9.

Besides, the weakly connected expansion of the equilibrium point we did in Section 3.2.4 can be derived in this case (see Theorem B.10). At the first order, this leads to the equilibrium connectivity

$$W^{*}=\frac{\tilde{p}l}{1+\lambda }\left(\lambda +{∥v∥}_{1}^{2}{\tilde{C}}^{0,0}\right)+O\left({\tilde{p}}^{2}{∥v∥}_{1}\right).$$

**Fig. 4:**
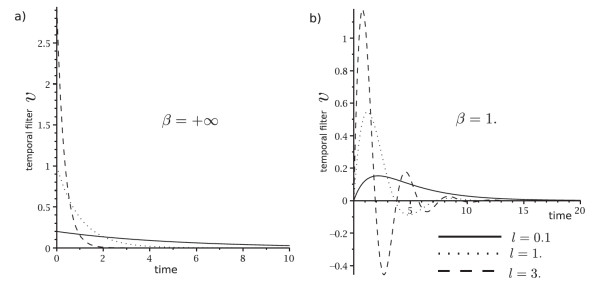
These represent the temporal filter v:t↦v(t) for different parameters. (**a**) When β=+∞, we are in the Hebbian linear case of Appendix B.2. The temporal filters are just decaying exponentials which averaged the signal over a past window. (**b**) When the dynamics of the neurons and synapse are oscillatory damped, some oscillations appear in the temporal filters. The number of oscillations depends on Δ. If Δ is real, then there are no oscillations as in the previous case. However, when Δ becomes a pure imaginary number, it creates a few oscillations which are even more numerous if |Δ| increases.

The second order is given in Theorem B.10. The main difference with the Hebbian linear case is the shape of the temporal filters. Actually, the temporal filters have an oscillatory damped behavior if Δ is purely imaginary. These two cases are compared in Figure 4. 

**Fig. 5:**
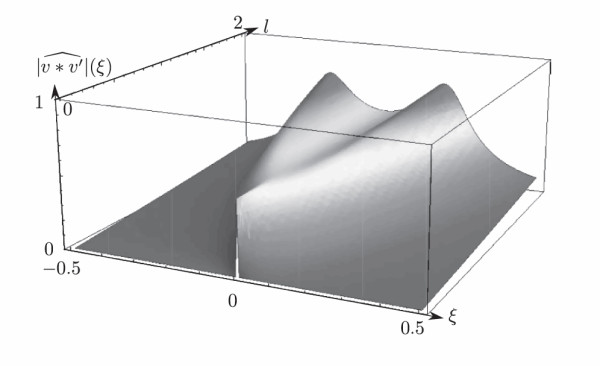
This is the spectral profile $\left|\hat{v*v^{\prime }}|(\xi \right)$ for β=1 and l∈]0,2], where $\hat{v*v^{\prime }}$ denotes the Fourier transform of $v*v^{\prime }$. When 4l<β, the filter reaches its maximum for the null frequency, but if *l* increases beyond $\frac{\beta }{4}$, the filter becomes a band-pass filter with long tails in $\frac{1}{\xi^{2}}$.

These oscillatory damped filters have the effect of amplifying a particular frequency of the input signal. As shown in Figure 5, if Δ is a pure imaginary number, then $D^{0,0}$ is the cross-correlation of the band-pass filtered inputs with themselves. This band-pass filter effect can also be observed in the higher-order terms of the weakly connected expansion. This suggests that the biophysical oscillatory behavior of neurons and synapses leads to selecting the corresponding frequency of the inputs and performing the same computation as for the Hebbian linear case of the previous section: computing the correlation of the (filtered) inputs. 

### 3.4 Asymmetric ‘STDP’ learning with correlated noise

**Fig. 6:**
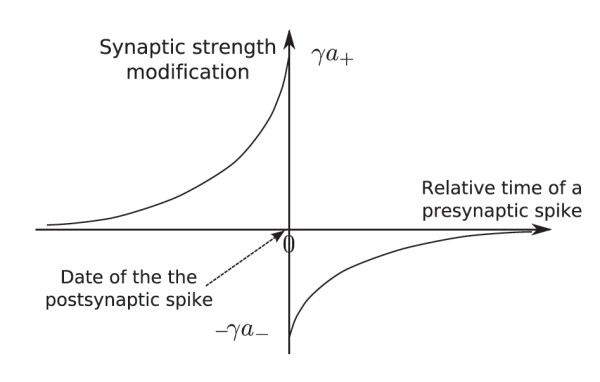
This figure represents the synapse strength modification when the post-synaptic neuron emits a spike. The *y*-axis corresponds to an additive or multiplicative update of the connectivity. For instance, in [28], this is $\frac{\Delta W_{ij}}{W_{ij}}$ for the negative part of the curve. However, we assume an additive update in this paper. The *x*-axis is the time at which a pre-synaptic spike reaches the synapse, relatively to the time of post-synaptic time chosen to be 0.

Here, we extend the results to temporally asymmetric learning rules and spatially correlated noise. We consider a learning rule that is similar to the spike-timing-dependent plasticity (STDP) which is closer to biological experiments than the previous Hebbian rules. It has been observed that the strength of the connection between two neurons depends mainly on the difference between the time of the spikes emitted by each neuron as shown in Figure 6; see [12]. 

Assuming that the decay time of the positive and negative parts of Figure 6 are equal, we approximate this function by $t\mapsto a_{+}g_{\gamma }(-t)-a_{-}g_{\gamma }(t)$, where $g_{\gamma }(t)=\gamma e^{-\gamma t}H(t)$. Actually, this corresponds to ${\dot{W^{\epsilon }}}_{ij}=-\kappa W_{ij}^{\epsilon }+a_{+}v_{i}(v_{j}^{\epsilon }*g_{\gamma })-a_{-}(v_{i}^{\epsilon }*g_{\gamma })v_{j}^{\epsilon }$. If the neuron has a spiking behavior, then the term $a_{+}v_{i}^{\epsilon }(t)(v_{j}^{\epsilon }*g_{\gamma })(t)$ is significant when the post-synaptic neuron *i* is spiking at time *t*, and then it counts the number of previous spikes from the pre-synaptic neuron *j* that might have caused the post-synaptic spike. This calculus is weighted by an exponentially decaying function. This accounts for the left part of Figure 6. The last term $-a_{-}(v_{i}^{\epsilon }*g_{\gamma })v_{j}^{\epsilon }$ takes the opposite perspective. It is significant when the pre-synaptic neuron *j* is spiking and counts the number of previous spikes from the post-synaptic neuron *i* that are not likely to have been caused by the pre-synaptic neuron. The computation is also weighted by the mirrored function of an exponentially decaying function. This accounts for the right part of Figure 6. This leads to the coupled system

(17) $$\left\{ \begin{array}{c} dv^{\epsilon }=\frac{1}{\epsilon_{1}}(f(v^{\epsilon })+W\cdot v^{\epsilon }+u(\frac{t}{\epsilon_{2}}))\;dt+\frac{1}{\sqrt{\epsilon_{1}}}\Sigma \cdot dB(t),\\ \frac{dW^{\epsilon }}{dt}=G(v^{\epsilon },W^{\epsilon })=-\kappa W^{\epsilon }+a_{+}v^{\epsilon }⊗(v^{\epsilon }*g_{\gamma })-a_{-}(v^{\epsilon }*g_{\gamma })⊗v^{\epsilon }, \end{array}\right.$$

where the non-linear intrinsic dynamics of the neurons is represented by *f*. Indeed, the term ${\left\{a_{+}v^{\epsilon }(t)⊗(v^{\epsilon }*g_{\gamma })(t)\right\}}_{ij}=a_{+}v_{i}^{\epsilon }(t){\left(v^{\epsilon }*g_{\gamma }\right)}_{j}(t)$ is negligible when the neuron is quiet and maximal at the top of the spikes emitted by neuron *i*. Therefore, it records the value of the pre-synaptic membrane potential weighted by the function $g_{\gamma }$ when the post-synaptic neuron spikes. This accounts for the positive part of Figure 6. Similarly, the negative part corresponds to $-a_{-}(v^{\epsilon }*g_{\gamma })⊗v^{\epsilon }$.

Actually, this formulation is valid for any non-linear activity with correlated noise. However, studying the role of STDP in spiking networks is beyond the scope of this paper since we are only able to have explicit results for models with linear activity. Therefore, we will assume that the activity is linear while keeping the learning rule as it was derived in the spiking case, *i.e.*, we assume f(v)=−lv=−L⋅v in the system above.

We also use the trick of adding additional variables to get rid of the history-dependency. This reads

$$\left\{ \begin{array}{c} d\left(\begin{array}{c} v^{\epsilon }\\ z^{\epsilon } \end{array}\right)=\frac{1}{\epsilon_{1}}\left[\left(\begin{array}{cc} W-L & 0\\ \gamma & -\gamma \end{array}\right)\left(\begin{array}{c} v^{\epsilon }\\ z^{\epsilon } \end{array}\right)+\left(\begin{array}{c} u(\frac{t}{\epsilon_{2}})\\ 0 \end{array}\right)\right]\;dt+\left(\begin{array}{c} \frac{\sigma }{\sqrt{\epsilon_{1}}}\;dB(t)\\ \frac{\sigma_{z}}{\sqrt{\epsilon_{1}}}\;dB(t) \end{array}\right),\\ \frac{dW^{\epsilon }}{dt}=-\kappa W^{\epsilon }+a_{+}v^{\epsilon }⊗z^{\epsilon }-a_{-}z^{\epsilon }⊗v^{\epsilon }. \end{array}\right.$$

In this framework, the method exposed in Section 3.2 holds with small changes. First, the well-posedness assumption becomes

**Assumption 3.2** There exists p∈]0,1[ such that

$$\frac{\left|a_{+}|+|a_{-}\right|}{p(1-p)}\left(\frac{s^{2}\gamma }{2(1+\gamma /l-p)}+\frac{u_{m}^{2}}{\left(1-p\right)}\right)<\kappa l^{3},$$

where $s^{2}$ is the maximal eigenvalue of $\Sigma \cdot \Sigma^{\prime }$.

Under this assumption, the system is asymptotically well posed in probability (Theorem B.11). And we show the averaged system is

(18) $$\frac{dW}{dt}=\bar{G}(W)=-\kappa W+\frac{u_{m}^{2}(|a_{+}|+|a_{-}|)}{l^{2}}\sum_{k,q=0}^{+\infty }\frac{W^{k}}{l^{k}}\cdot D^{k,q}\cdot \frac{{W^{\prime }}^{q}}{l^{q}}+Q,$$

where we have used Theorem B.12 to expand the correlation term. The noise term **Q** is equal to $Q_{11}\cdot {\left(L+\gamma -W^{\prime }\right)}^{-1}$, where $Q_{11}$ is the unique solution of the Lyapunov equation $(W-L)\cdot Q_{11}+Q_{11}\cdot (W^{\prime }-L)+\Sigma \cdot \Sigma^{\prime }=0$. Lemma D.1 gives a solution for this equation which leads to $Q=\gamma \sum_{k=0}^{+\infty }W^{k}\cdot \Sigma \cdot \Sigma^{\prime }\cdot {\left(2L-W^{\prime }\right)}^{-(k+1)}\cdot {\left(L+\gamma -W^{\prime }\right)}^{-1}$. In equation (18), the correlation matrices $D^{k,q}$ are given by

$$D^{k,q}=\frac{1}{u_{m}^{2}\tau (|a_{+}|+|a_{-}|)}\left(u*g_{l/\mu }^{\left(k+1\right)}*\left(a_{+}g_{\gamma }^{\prime }-a_{-}g_{\gamma }\right)\right)\cdot {\left(u*g_{l/\mu }^{\left(q+1\right)}\right)}^{\prime }.$$

According to Theorem B.13, the system is also globally asymptotically convergent to a single equilibrium, which we study in the following.

We perform a weakly connected expansion of the equilibrium connectivity of system (18). As shown in Theorem B.14, the first order of the expansion is

$$W^{*}=\frac{\tilde{p}l}{1+\lambda }\left(\lambda (\alpha_{+}-\alpha_{-})\frac{\Sigma \cdot \Sigma^{\prime }}{d}+D^{0,0}\right)+O\left({\tilde{p}}^{2}\right).$$

Writing $D^{0,0}=\frac{1}{u_{m}^{2}\tau (|a_{+}|+|a_{-}|)}(S+A)$, where **S** is symmetric and **A** is skew-symmetric, leads to

$$\begin{array}{rcl} S & = & \frac{a_{+}-a_{-}}{2}u*g_{l/\mu }*\left(g_{\gamma }^{\prime }+g_{\gamma }\right)\cdot {\left(u*g_{l/\mu }\right)}^{\prime },\\ A & = & \frac{a_{+}+a_{-}}{2}u*g_{l/\mu }*\left(g_{\gamma }^{\prime }-g_{\gamma }\right)\cdot {\left(u*g_{l/\mu }\right)}^{\prime }. \end{array}$$

According to Lemma C.1, the symmetric part is very similar to the trace learning case in Section 3.3. Applying Lemma C.2 leads to

(19) $$\begin{array}{rl} S & =(a_{+}-a_{-})(u*g_{l/\mu }*g_{\gamma })\cdot {\left(u*g_{l/\mu }*g_{\gamma }\right)}^{\prime },\\ A & =\frac{a_{+}+a_{-}}{\gamma }\left(\frac{du}{dt}*g_{l/\mu }*g_{\gamma }\right)\cdot {\left(u*g_{l/\mu }*g_{\gamma }\right)}^{\prime }. \end{array}$$

Therefore, the STDP learning rule simply adds an antisymmetric part to the final connectivity keeping the symmetric part as the Hebbian case. Besides, the antisymmetric part corresponds to computing the cross-correlation of the inputs with its derivative. For high-order terms, this remains true although the temporal profiles are different from the first order. These results are in line with previous works underlying the similarity between STDP learning and differential Hebbian learning, where $G(v)∼\dot{v}⊗v$; see [29]. 

**Fig. 7:**
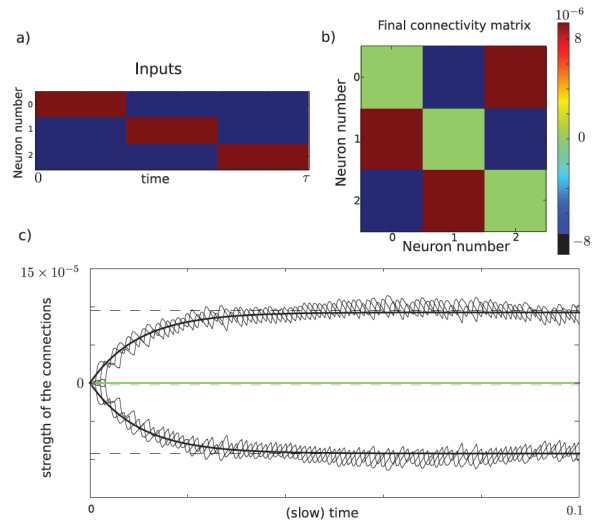
Antisymmetric STDP learning for a network of n=3 neurons. (**a**) Temporal evolution of the inputs to the network. The three neurons are successively and periodically excited. The *red color* corresponds to an excitation of 1 and the *blue* to no excitation. (**b**) Equilibrium connectivity. The matrix is antisymmetric and shows that neurons excite one of their neighbors and are inhibited by the other. (**c**) Temporal evolution of the connectivity strength. The colors correspond to those of (b). The connectivity of system (17) corresponds to the *plain thin oscillatory curves*. The connectivity of the averaged system (18) (with k,q<4) corresponds to the *plain thick lines*. Note that each curve corresponds to the superposition of three connections which remain equal through learning. The *dashed curves* correspond to the antisymmetric part in (19). The parameters chosen for this simulation were l=10, κ=100, γ=3, $a_{+}=a_{-}=1$, τ=3, σ=0.001, μ=1, ϵ=0.001. The system was simulated on the fast time-scale during T=10,000 time steps of size dt=0.01.

Figure 7 shows an example of purely antisymmetric STDP learning, *i.e.*, $a_{+}=a_{-}$. The final connectivity matrix is therefore antisymmetric as shown in Figure 7(b) and the noise has no impact on learning. It shows the network finally approximates the connectivity given in (19). 

## 4 Discussion

We have applied temporal averaging methods on slow/fast systems modeling the learning mechanisms occurring in linear stochastic neural networks. When we make sure the connectivity remains small, the dynamics of the averaged system appears to be simple: the connectivity always converges to a unique equilibrium point. Then, we performed a weakly connected expansion of this final connectivity whose terms are combinations of the noise covariance and the lagged correlations of the inputs: the first-order term is simply the sum of the noise covariance and the correlation of the inputs. 

• As opposed to the former input/ouput vision of the neurons, we have considered the membrane potential **v** to be the solution of a dynamical system. The consequence of this modeling choice is that not only the spatial correlations, but also the temporal correlations are learned. Due to the fact we take the transients into account, the activity never converges but it lives between the representation of the inputs. Therefore, it links concepts together.

The parameter *μ* is the ratio of the time-scales between the inputs and the activity variable. If μ=0, the inputs are infinitely slow and the activity variable has enough time to converge towards its equilibrium point. When *μ* grows, the dynamics becomes more and more transient, it has no time to converge. Therefore, if the inputs are extremely slow, the network only learns the spatial correlation of the inputs. If the inputs are fast, it also learns the temporal correlations. This is illustrated in Figure 3.

This suggests that learning associations between concepts, for instance, learning words in two different languages, may be optimized by presenting two words to be associated circularly with a certain frequency. Indeed, increasing the frequency (with a fixed duration of exposition to each word) amounts to increasing *μ*. Therefore, the network learns better the temporal correlations of the inputs and thus strengthens the link between these two concepts.

• According to the model of resonator neuron [30], Section 3.3 suggests that neurons and synapses with a preferred frequency of oscillation will preferably extract the correlation of the inputs filtered by a band pass filter centered on the intrinsic frequency of the neurons.

Actually, it has been observed that the auditory cortex is tonotopically organized, *i.e.*, the neurons are arranged by frequency [31]. It is traditionally thought that this is achieved thanks to a particular connectivity between the neurons. We exhibit here another mechanism to select this frequency which is solely based on the parameters of the neurons: a network with a lot of different neurons whose intrinsic frequencies are uniformly spread is likely to perform a Fourier-like operation: decomposing the signal by frequency. 

In particular, this emphasizes the fact that the network does not treat space and time similarly. Roughly speaking, associating several pictures and associating several sounds are therefore two different tasks which involve different mechanisms.

• In this paper, the original hierarchy of the network has been neglected: the network is made of neurons which receive external inputs. A natural way to include a hierarchical structure (with layers for instance), without changing the setup of the paper, is therefore to remove the external input to some neurons. However, according to Theorem 3.5 (and its extensions Theorems B.10 and B.14), one can see that these neurons will be disconnected from the others at the first order (if the noise is spatially uncorrelated). Linear activities imply that the high level neurons disconnect from others, which is a problem. In fact, one can observe that the second-order term in Theorem 3.5 is not null if the noise matrix **Σ** is not diagonal. In fact, this is the noise between neurons which will recruit the high level neurons to build connections from and to them.

It is likely that a significant part of noise in the brain is locally induced, *e.g.*, local perturbations due to blood vessels or local chemical signals. In a way, the neurons close to each other share their noise and it seems reasonable to choose the matrix **Σ** so that it reflects the biological proximity between neurons. In fact, **Σ** specifies the original structure of the network and makes it possible for close-by neurons to recruit each other.

Another idea to address hierarchy in networks would be to replace the synaptic decay term −κW by another homeostatic term [32] which would enforce the emergence of a strong hierarchical structure. 

• It is also interesting to observe that most of the noise contribution to the equilibrium connectivity for STDP learning (see Theorem B.14) vanishes if the learning is purely skew-symmetric, *i.e.*, $a_{+}=a_{-}$. In fact, it is only the symmetric part of learning, *i.e.*, the Hebbian mechanism, that writes the noise in the connectivity.

• We have shown that there is a natural analogous STDP learning for spiking neurons in our case of linear neurons. This asymmetric rule converges to a final connectivity which can be decomposed into symmetric and skew-symmetric parts. The first one is similar to the symmetric Hebbian learning case, emphasizing that the STDP is nothing more than an asymmetric Hebbian-like learning rule. The skew-symmetric part of the final connectivity is the cross-correlation between the inputs and their derivatives.

This has an interesting signification when looking at the spontaneous activity of the network post-learning. In fact, if we assume that the inputs are generated by an autonomous system $\frac{du}{dt}=\zeta (u)$, then according to the bottom equation in formula (19), the spontaneous activity is governed by

$$dv=\left(\zeta (u)\cdot u^{\prime }\cdot v-lv\right)\;dt+\Sigma \cdot dB(t).$$

In a way, the noise terms generate random patterns which tend to be forgotten by the network due to the leak term −lv. The only drift is due to $\zeta (u)\cdot u^{\prime }\cdot v≃E_{\langle v,u\rangle }(\zeta (u))$ which is the expectation of the vector field defining the dynamics of inputs with a measure being the scalar product between the activity variable and the inputs. In other words, if the activity is close to the inputs at a given time $t^{*}\in R_{+}$, *i.e.*, $\langle v,u(t^{*})\rangle$ is large, then the activity will evolve in the same direction as what this input would have done. The network has modeled the temporal structure of the inputs. The spontaneous activity predicts and replays the inputs the network has learned.

There are still numerous challenges to carry on in this direction.

First, it seems natural to look for an application of these mathematical methods to more realistic models. The two main limitations of the class of models we study in Section 3 are (i) the activity variable is governed by a linear equation and (ii) all the neurons are assumed to be identical. The mathematical analysis in this paper was made possible by the assumption that the neural network has a linear dynamics, which does not reflect the intrinsic non-linear behavior of the neurons. However, the cornerstone of the application of temporal averaging methods to a learning neural network, namely Property 3.3, is similar to the behavior of Poisson processes [26] which has useful applications for learning neural networks [19,20]. This suggests that the dynamics studied in this paper might be quite similar to some non-linear network models. Studying more rigorously the extension of the present theory to non-linear and heterogeneous models is the next step toward a better modeling of biologically plausible neural networks. 

Second, we have shown that the equilibrium connectivity was made of a symmetric and antisymmetric term. In terms of statistical analysis of data sets, the symmetric part corresponds to classical correlation matrices. However, the antisymmetric part suggests a way to improve the purely correlation-based approach used in many statistical analyses (*e.g.*, PCA) toward a causality-oriented framework which might be better suited to deal with dynamical data.

## Appendix A: Stochastic and periodic averaging

### A.1 Long-time behavior of inhomogeneous Markov processes

In order to construct the averaged vector field ${\bar{G}}_{\mu }(w)$ in the time-scale matching case (0<μ<∞), one needs to understand properly the long-time behavior of the rescaled inhomogeneous frozen process

(20) $$dv=F(v,w_{0},\mu t)\;dt+\Sigma (v,w_{0})\;dB(t).$$

Under regularity and dissipativity conditions, [5] proves the following general result about the asymptotic behavior of the solution of

$$\begin{array}{rcl} dX_{t} & = & b(X_{t},t)\;dt+\sigma (X_{t},t)\;dB(t),\;t>s,\\ X_{s} & = & x, \end{array}$$

where t→b(x,t) and t→σ(x,t) are *τ*-periodic.

The first point of the following theorem gives the definition of *evolution systems of measures*, which generalizes the notion of invariant measures in the case of inhomogeneous Markov processes. The exponential estimate of 2. in the following theorem is a key point to prove the averaging principle of Theorem 2.2.

**Theorem A.1** ([5]) 

1. *There exists a uniqueτ*-*periodic family of probability measures*{μ(s,⋅),s∈R}*such that for all functionsϕcontinuous and bounded*,

$$\int_{x\in R^{p}}E\left[\phi (X_{t})\right]\mu (s,dx)=\int_{x\in R^{p}}\phi (x)\mu (t,dx).$$

*Such a family is called evolution systems of measures*.

2. *Furthermore*, *under stronger dissipativity condition*, *the convergence of the law ofXtoμis exponentially fast*. *More precisely*, *for any*r∈(1,+∞), *there exist*M>0*and*ω<0*such that for allϕin the space ofp*-*integrable functions with respect to*μ(t,⋅), $L^{r}(R^{p},\mu (t,\cdot ))$,

$$\begin{array}{rcl} & & \int_{x\in R^{p}}{∥E\left[\phi \left(X_{t}^{s,x}\right)\right]-\int_{x^{\prime }\in R^{p}}\phi \left(x^{\prime }\right)\mu \left(t,dx^{\prime }\right)∥}^{r}\mu (s,dx)\\ & & \;\leq Me^{\omega (t-s)}\int_{x\in R^{p}}{∥\phi (x)∥}^{r}\mu (t,dx). \end{array}$$

### A.2 Proof of Property 2.3

**Property A.2***If there exists a smooth subset* ℰ *of*$R^{q}$*such that*

1. *The functionsF*, *G*, **Σ***satisfy Assumptions *2.1-2.3 *restricted on*$R^{p}\times E$.

2. ℰ *is invariant under the flow of*${\bar{G}}_{\mu }$, *as defined in* (7).

*Then for any initial condition*$w_{0}\in E$, *system* (4) *is asymptotically well posed in probability and*$w^{\epsilon }$*satisfies the conclusion of Theorem *2.2.

*Proof* The idea of the proof is to truncate the original system, replacing *G* by a smooth truncation which coincides with *G* on ℰ and which is close to 0 outside ℰ. More precisely, for β>0, we introduce $\psi_{\beta }:R^{q}\to R^{q}$ a regular function (locally Lipschitz) such that $\psi_{\beta }(w)=0$ if w∉E or w∈∂E and $\lim_{\beta \to 0}\psi_{\beta }(w)=1$ if w∈E−∂E. We define

$${\tilde{G}}_{\beta }(v,w)=G(v,w)\psi_{\beta }(w).$$

Then, we introduce $\left({\tilde{v}}^{\epsilon ,\beta },{\tilde{w}}^{\epsilon ,\beta }\right)$ the solution of the auxiliary system

$$\begin{array}{rcl} d{\tilde{v}}^{\epsilon ,\beta } & = & \frac{1}{\epsilon_{1}}\left[F\left({\tilde{v}}^{\epsilon ,\beta },{\tilde{w}}^{\epsilon ,\beta },\frac{t}{\epsilon_{2}}\right)\right]\;dt+\frac{1}{\sqrt{\epsilon_{1}}}\Sigma \left({\tilde{v}}^{\epsilon ,\beta },{\tilde{w}}^{\epsilon ,\beta }\right)\;dB(t),\\ d{\tilde{w}}^{\epsilon ,\beta } & = & {\tilde{G}}_{\beta }\left({\tilde{v}}^{\epsilon ,\beta },{\tilde{w}}^{\epsilon ,\beta }\right)\;dt \end{array}$$

with the same initial condition as $\left(v^{\epsilon },w^{\epsilon }\right)$.

Let T,δ,η>0 be three positive reals. Let us introduce a few more notations. We will need to consider a subset of ℰ defined by

$$E^{\beta }:=\left\{w\in E;∥\psi_{\beta }(w)-1∥\leq {\left(\eta \right)}^{1/2}\delta \right\}.$$

We also introduce the following stopping times:

$$\begin{array}{rcl} \tau_{\epsilon } & := & \inf\left\{t\geq 0;w_{t}^{\epsilon }\in \partial E\right\},\;\tau_{\epsilon }^{\beta }:=\inf\left\{t\geq 0;w_{t}^{\epsilon }\in \partial E^{\beta }\right\},\\ {\tilde{\tau }}_{\epsilon } & := & \inf\left\{t\geq 0;{\tilde{w}}_{t}^{\epsilon ,\beta }\in \partial E\right\},\;{\tilde{\tau }}_{\epsilon }^{\beta }:=\inf\left\{t\geq 0;{\tilde{w}}_{t}^{\epsilon ,\beta }\in \partial E^{\beta }\right\}. \end{array}$$

Finally, we define $T_{\epsilon }:=\min(T,\tau_{\epsilon },{\tilde{\tau }}_{\epsilon })$ and $T_{\epsilon }^{\beta }:=\min(T,\tau_{\epsilon }^{\beta },{\tilde{\tau }}_{\epsilon }^{\beta })$.

Let us remark at this point that in order to prove that $P[\tau_{\epsilon }\geq T]\to 1$ (which is our aim), it is sufficient to work on the bounded stopping time $\min(T,\tau_{\epsilon })$, since $P[\tau_{\epsilon }\geq T]=P[\min(T,\tau_{\epsilon })\geq T]$. In other words, the realizations of $w^{\epsilon }$ which stay longer than *T* inside ℰ are not problematic. Therefore, we introduce $\hat{\tau_{\epsilon }}:=\min(T,\tau_{\epsilon })$.

Our first claim is that on finite time intervals [0,T], ${\tilde{w}}^{\epsilon ,\beta }$ is a good approximation of $w^{\epsilon }$ inside ℰ as long as one chooses *β* sufficiently small. To prove our claim, we proceed in two steps, first working inside $E^{\beta }$ and then in $E-E^{\beta }$: 

1. For any β>0, one controls the difference between $w^{\epsilon }$ and ${\tilde{w}}^{\epsilon ,\beta }$ on $E^{\beta }$ since one controls the difference between the drifts. By an application of Lemma A.3 below (we need here the moment Assumption 2.3(i)), there exists a constant *C* (which may depend on T,β,…) such that

(21) $$E\left[\underset{0\leq t\leq T_{\epsilon }^{\beta }}{\sup}{∥w_{t}^{\epsilon }-{\tilde{w}}_{t}^{\epsilon ,\beta }∥}^{2}\right]\leq C\eta \delta^{2}.$$

We conclude by an application of the Markov inequality, implying

(22) $$P\left[\underset{0\leq t\leq T_{\epsilon }^{\beta }}{\sup}∥w_{t}^{\epsilon }-{\tilde{w}}_{t}^{\epsilon ,\beta }∥>\delta \right]\leq \frac{1}{\delta^{2}}E\left[\underset{0\leq t\leq T_{\epsilon }^{\beta }}{\sup}{∥w_{t}^{\epsilon }-{\tilde{w}}_{t}^{\epsilon ,\beta }∥}^{2}\right]\leq C\eta .$$

2. One needs now to control the situation outside $E^{\beta }$, that is, on $E-E^{\beta }$. The idea is that while one does not control the difference between *G* and ${\tilde{G}}_{\beta }$ anymore, one can still choose *β* sufficiently small such that $E^{\beta }$ becomes arbitrary close to ℰ, hence implying that ${\hat{\tau }}_{\epsilon }$ and $T_{\epsilon }^{\beta }$ are arbitrary close with high probability, namely

(23) $$\forall \theta ,\lambda >0,\exists \beta >0,\;P\left[\tau_{\epsilon }-T_{\epsilon }^{\beta }>\lambda \right]<\theta .$$

With $\theta ={\left(\delta \eta \right)}^{2}$ and λ=δη, one obtains that for sufficiently small *β*,

(24) $$P\left[\hat{\tau_{\epsilon }}-T_{\epsilon }^{\beta }>\eta \delta \right]<{\left(\eta \delta \right)}^{2}.$$

Let us denote $S:=\sup_{T_{\epsilon }^{\beta }\leq t\leq \hat{\tau_{\epsilon }}}∥w_{t}^{\epsilon }-{\tilde{w}}_{t}^{\epsilon ,\beta }∥$. Then, one can split the calculus of E[S] according to the event $A=\{\hat{\tau_{\epsilon }}-T_{\epsilon }^{\beta }>\eta \delta \}$:

$$\begin{array}{rcl} E[S] & = & E[SI_{A}]+E[SI_{A^{c}}]\\ & \leq & {\left(2K_{G}TP[A]\right)}^{1/2}+{\left(2K_{G}E\left[{\left({\hat{\tau }}_{\epsilon }-T_{\epsilon }^{\beta }\right)}^{2}I_{A^{c}}\right]\right)}^{1/2}\\ & \leq & C_{2}\eta \delta , \end{array}$$

where we have used the Cauchy-Schwarz inequality and the moment Assumption 2.3(ii) (yielding the constant $K_{G}$) in the second line.

So, we deduce by the Markov inequality that $\sup_{T_{\epsilon }^{\beta }\leq t\leq {\hat{\tau }}_{\epsilon }}∥w_{t}^{\epsilon }-{\tilde{w}}_{t}^{\epsilon ,\beta }∥$ is arbitrary small in probability.

 From the combination of 1. and 2., we deduce that one can choose *β* small enough such that

(25) $$P\left[\underset{0\leq t\leq T\wedge \tau_{\epsilon }}{\sup}∥w_{t}^{\epsilon }-{\tilde{w}}_{t}^{\epsilon ,\beta }∥>\delta \right]\leq (C_{1}+C_{2})\eta .$$

We can now proceed to the application of Theorem 2.2 to the truncated system. As $\left({\tilde{v}}^{\epsilon ,\beta_{0}},{\tilde{w}}^{\epsilon ,\beta_{0}}\right)$ remains in $R^{p}\times E$, one can extend smoothly *F* and **Σ** outside ℰ so that (F,Σ) satisfies Assumptions 2.1-2.2. Therefore, one can apply Theorem 2.2 to the auxiliary system: for all δ,T>0,

$$\lim_{\epsilon \to 0}^{\mu }P\left[\underset{t\in [0,T]}{\sup}∥{\tilde{w}}_{t}^{\epsilon ,\beta_{0}}-w_{t}∥>\delta \right]=0,$$

where **w** is defined by (8). As a consequence, there exists $\epsilon_{0}$ such that for all *ϵ* with $∥\epsilon ∥<\epsilon_{0}$,

$$P\left[\underset{t\in [0,T]}{\sup}∥{\tilde{w}}_{t}^{\epsilon ,\beta_{0}}-w_{t}∥>\delta \right]<\eta .$$

Then, as $\left|{\hat{w}}_{t}^{\epsilon }-w_{t}|\leq |{\hat{w}}_{t}^{\epsilon }-{\tilde{w}}_{t}^{\epsilon ,\beta_{0}}|+|{\tilde{w}}_{t}^{\epsilon ,\beta_{0}}-w_{t}\right|$, one deduces that for all *ϵ* with $∥\epsilon ∥<\epsilon_{0}$,

$$P\left[\underset{t\in [0,T]}{\sup}∥{\hat{w}}_{t}^{\epsilon }-w_{t}∥>\delta \right]<(C_{1}+C_{2}+1)\eta ,$$

that is to say,

$$\lim_{\epsilon \to 0}^{\mu }P\left[\underset{t\in [0,T]}{\sup}∥{\hat{w}}_{t}^{\epsilon }-w_{t}∥>\delta \right]=0.$$

We know by assumption 2. of the statement of Property 2.3, for all t≥0, $w_{t}\in E$, so we conclude the proof by observing that for all T>0,

$$\lim_{\epsilon \to 0}P[\tau_{\epsilon }\geq T]=1.$$

 □

In the following lemma, we show that the solutions of two SDEs, whose drifts are close on a subset of the state space, remain close on a finite time interval. The difficulty here lies in the fact that we deal with only *locally Lipschitz* coefficients.

**Lemma A.3***Supposexandyare solutions*, *with identical initial conditions in*$H\subset R^{n}$, *of the following stochastic differential equations in*$R^{n}$:

(26) $$dx_{t}=a(x_{t},t)\;dt+b(x_{t},t)\;dB(t),$$

(27) $$dy_{t}=h(y_{t})a(y_{t},t)\;dt+b(y_{t},t)\;dB(t).$$

*Let*T>0*be a fixed time*. *We define*

$$\tau_{H}=\min\left(\inf\{t\geq 0;x_{t}\in \partial H\},\inf\{t\geq 0;y_{t}\in \partial H\}\right).$$

*We make the following assumptions*: 

1. *Approximation assumption*:

$$\underset{y\in H}{\sup}∥h(y)-1∥\leq \xi ;$$

2. *Local Lipschitz assumption*: *for all*$a,b\in R^{n}$*with*max(∥a∥,∥b∥)≤R, *there exists a constant*$C_{R}$*such that*

$${∥a(x,t)-a(y,t)∥}^{2}\leq C_{R}{∥x-y∥}^{2};$$

3. *Boundedness assumption*: *there exists*p>2*and*A>0*such that*

$$E\left[\underset{0\leq t\leq T}{\sup}{∥x_{t}∥}^{p}\right]\leq A\;\text{and}\;E\left[\underset{0\leq t\leq T}{\sup}{∥y_{t}∥}^{p}\right]\leq A,$$

*and if*∥x∥≤R, *then there exists*$K_{R}$*such that*$∥a(x)∥\leq K_{R}$.

*Under the above assumptions*, *there exists a constantC* (*depending on the quantities defined above*, *but not onξ*) *such that*

(28) $$E\left[\underset{0\leq t\leq \min(T,\tau_{H})}{\sup}{∥x_{t}-y_{t}∥}^{2}\right]\leq C\xi^{2}.$$

*Proof* Although the Lipschitz constant is not bounded on ℋ, we can use the boundedness assumption to show that the probability of reaching a level *R* before time *T* will be very small for large *R*, and then use the classical strategy inside $\left\{∥x_{t}∥\leq R\right\}$ where everything works as if the coefficients were globally Lipschitz. A similar strategy is used in [33] to prove a strong convergence theorem for the Euler scheme without the global Lipschitz assumption. We adapt here the ideas of their proof to our setting. 

Therefore, we introduce the following stopping times:

$$\begin{array}{rcl} \theta_{R} & := & \inf\left\{t\geq 0;∥x_{t}∥\geq R\right\},\\ \theta_{R}^{\beta } & := & \inf\left\{t\geq 0;∥y_{t}∥\geq R\right\}\;\text{and}\;\rho :=\min\left(\theta_{R},\theta_{R}^{\beta },\tau_{H}\right). \end{array}$$

We also denote $e(t):=x_{t}-y_{t}$.

Splitting the following expectation according to the value of *ρ*, and applying the Young inequality,

$$ab\leq \frac{d}{r}a^{r}+\frac{1}{qd^{q/r}}b^{q}\;\text{for }r^{-1}+q^{-1}=1\text{ and any }a,b,d>0,$$

we obtain, for any d>0,

$$\begin{array}{rcl} & & E\left[\underset{0\leq t\leq \min(T,\tau_{H})}{\sup}{∥e(t)∥}^{2}\right]\\ & & \;\leq E\left[\underset{0\leq t\leq \min(T,\tau_{H})}{\sup}{∥e\left(\min(t,\rho )\right)∥}^{2}\right]\\ & & \;+\frac{2d}{p}E\left[\underset{0\leq t\leq T}{\sup}s{∥e(t)∥}^{p}\right]+\frac{1-2/p}{d^{2/(p-2)}}P\left[\theta_{R}\leq T\text{ or }\theta_{R}^{\beta }\leq T\right]. \end{array}$$

Then we use the boundedness assumption and the Markov inequality to deduce that

$$P\left[\theta_{R}\leq T\text{ or }\theta_{R}^{\beta }\leq T\right]\leq \frac{2A}{R^{p}}\text{ and }E\left[\underset{0\leq t\leq T}{\sup}{∥e(t)∥}^{p}\right]\leq 2^{p}A.$$

Now, we can focus on the supremum of the error before time *ρ*. We first apply the Cauchy-Schwarz inequality

$$\begin{array}{rcl} {∥e\left(\min(t,\rho )\right)∥}^{2} & = & ∥\int_{0}^{\min(t,\rho )}\left(a(x_{s},s)-h(y_{s})a(y_{s},s)\right)\;ds\\ & & +\int_{0}^{\min(t,\rho )}\left(b(x_{s},s)-b(y_{s},s)\right)\;dB(s){∥}^{2}\\ & \leq & 2[T\int_{0}^{\min(t,\rho )}{∥a(x_{s},s)-h(y_{s})a(y_{s},s)∥}^{2}\;ds\\ & & +{∥\int_{0}^{\min(t,\rho )}\left(b(x_{s},s)-b(y_{s},s)\right)\;dB(s)∥}^{2}]. \end{array}$$

Then, we use the local Lipschitz and the boundedness assumptions, together with the Doob inequality (the first inequality) to deal with the stochastic integral: for any u>0,

$$\begin{array}{rcl} & & E\left[\underset{0\leq t\leq u}{\sup}{∥e\left(\min(t,\rho )\right)∥}^{2}\right]\\ & & \;\leq 2E[T\int_{0}^{\min(u,\rho )}{∥a(x_{s},s)-h(y_{s})a(y_{s},s)∥}^{2}\;ds\\ & & \;+4\int_{0}^{\min(u,\rho )}{∥\sigma (x_{s},s)-\sigma (y_{s},s)∥}^{2}\;ds]\\ & & \;\leq 2E\left[TC_{R}\int_{0}^{\min(u,\rho )}{∥x_{s}-y_{s}∥}^{2}\;ds+T^{2}K_{R}^{2}\xi^{2}+4C_{R}\int_{0}^{\min(u,\rho )}{∥x_{s}-y_{s}∥}^{2}\;ds\right]\\ & & \;\leq 2C_{R}(T+4)E\left[\int_{0}^{\min(u,\rho )}\underset{0\leq r\leq s}{\sup}\left\{{∥x_{\min(r,\rho )}-y_{\min(r,\rho )}∥}^{2}\right\}\;ds\right]+2T^{2}K_{R}^{2}\xi^{2}. \end{array}$$

We then apply the Gronwall lemma

(29) $$E\left[\underset{0\leq t\leq T}{\sup}{∥e\left(\min(t,\rho )\right)∥}^{2}\right]\leq 2T^{2}K_{R}^{2}\xi^{2}e^{2C_{R}(T+4)}.$$

Finally, we choose *d* small enough such that

$$\frac{2^{p+1}dA}{p}\leq \xi^{2},$$

and *R* large enough such that

$$\frac{2A(p-2)}{R^{p}pd^{2/(p-2)}}\leq \xi^{2}$$

yielding

$$E\left[\underset{0\leq t\leq \min(T,\tau_{H})}{\sup}{∥x_{t}-y_{t}∥}^{2}\right]\leq \left(2+2T^{2}K_{R}^{2}e^{2C_{R}(T+4)}\right)\xi^{2}.$$

 □

## Appendix B: Proofs of Section 3

### B.1 Notations and definitions

Throughout the paper, lower-case normal letters are constants, lower-case bold letters are vectors or vector-valued functions, and upper-case bold letters are matrices. 

• $l,\kappa ,\tau ,\epsilon_{1},\epsilon_{2},\mu ,\sigma^{2},\beta ,\gamma ,a_{\pm }\in R_{+}$ are parameters of the network. We also define $\Delta =\sqrt{1-4\frac{l}{\beta }}$ for Section 3.3 and $\Sigma \in R^{n\times n}$, a fixed noise matrix, for Section 3.4. We write $s^{2}=⦀\Sigma \cdot \Sigma^{\prime }⦀$.

• n∈N is the number of neurons in the network.

• $v\in C^{1}(R_{+},R^{n})$ is the field of membrane potential in the network.

• $u\in C^{1}(R_{+},R^{n})$ is the field of inputs to the network. We write

$$u_{m}=\underset{t\in R_{+}}{\sup}{∥u(t)∥}_{2}.$$

• $v⊗u\in C^{1}(R_{+},R^{n\times n})$ is the tensor product between **u** and **v**, which simply means ${\left\{u⊗v\right\}}_{ij}(t)=u_{i}(t)v_{j}(t)$.

• $W\in C^{1}(R_{+},R^{n\times n})$ is the connectivity of the network. Throughout the paper, we assume W(0)=0.

• 〈x,y〉 is the scalar product between two vectors $x,y\in R^{n}$.

• ${∥u(t)∥}_{p}$ for p=1,2 is the $L^{p}$ norm of $u(t)\in R^{n}$, *i.e.*, ${∥u(t)∥}_{p}={\left(\sum_{i=1}^{n}{\left|u_{i}(t)\right|}^{p}\right)}^{\frac{1}{p}}$. And similarly for the connectivity matrices of $R^{n\times n}$ with a double sum.

• $⦀W⦀=\sup_{x\in C^{n},∥x∥=1}|\langle x,W\cdot x\rangle |=\max_{i\in \{1,\ldots ,n\}}\{|\lambda_{i}|:\lambda_{i}\text{ is an eigenvalue of }W\}$.

• $J^{\prime }$ is the transpose of the matrix $J\in R^{n\times n}$.

• $x\cdot y^{\prime }\in R^{n\times n}$ is the cross-correlation matrix of two compactly supported and differentiable functions from ℝ to $R^{n}$, *i.e.*,

$${\left\{x\cdot y^{\prime }\right\}}_{ij}=\int_{-\infty }^{+\infty }x_{i}(t)y_{j}(t)\;dt.$$

• *H* is the Heaviside function, *i.e.*,

$$H(t)=\{\begin{array}{cc} 0 & \text{if }t\leq 0,\\ 1 & \text{if }t>0. \end{array}$$

• The real functions

(30) $$\begin{array}{rl} g_{\gamma }:t\mapsto & \gamma e^{-\gamma t}H(t),\\ v:t\mapsto & \frac{l}{\mu \Delta }\left(e^{-\frac{\beta }{2\mu }(1-\Delta )t}-e^{-\frac{\beta }{2\mu }(1+\Delta )t}\right)H(t),\\ w:t\mapsto & \frac{l}{2\mu \Delta }\left((1+\Delta )e^{-\frac{\beta }{2\mu }(1-\Delta )t}-(1-\Delta )e^{-\frac{\beta }{2\mu }(1+\Delta )t}\right)H(t) \end{array}$$

are integrable on ℝ.

#### B.1.1 Notations for the Appendix

The computations involve a lot of convolutions and, for readability of the Appendix, we introduce some new notations. Indeed, we rewrite the time-convolution between **u** and *g*, an integrable function on ℝ,

u∗g=u⋅G.

This suggests one should think of **v** as a semi-continuous matrix of $R^{n\times R}$ and of $G_{\gamma }$ as a continuous matrix of $R^{R\times R}$, such that $u_{it}=u_{i}(t)$ and $G_{st}=g(t-s)$. Indeed, in this framework the convolution with *g* is nothing but the continuous matrix multiplication between **v** and a continuous Toeplitz matrix generated row by row by *g*. Hence, the operator ‘⋅’ can be though of as a matrix multiplication.

Therefore, it is natural to define ${\left(u*g\right)}^{\prime }={\left(u\cdot G\right)}^{\prime }=G^{\prime }\cdot u^{\prime }$, where $G^{\prime }\in R^{R\times R}$ is the transpose of G, *i.e.*, the continuous Toeplitz matrix generated row by row by g(−⋅):t↦g(−t) and $u^{\prime }\in R^{R\times n}$. Thus, for *g* and *h*, two integrable functions on ℝ, we can rewrite

$$(x*g)\cdot {\left(y*h\right)}^{\prime }=x\cdot G\cdot H^{\prime }\cdot y^{\prime },$$

where G and ℋ are their associated continuous matrices. More generally, the bold curved letters G, V, W represent these continuous Toeplitz matrices which are well defined through their action as convolution operators with *g*, *v*, and *w*. The previous formulation naturally expresses the symmetry of relation (14).

### B.2 Hebbian learning with linear activity

In this part, we consider system (12).

#### B.2.1 Application of temporal averaging theory

**Theorem B.1***If Assumption *3.1 *is verified for*p∈]0,1[, *then system* (12) *is asymptotically well posed in probability and the connectivity matrix*$W^{\epsilon }$, *the solution of system* (12), *converges to***W**, *in the sense that for all*δ,T>0,

$$\lim_{\epsilon \to 0}^{\mu }P\left[\underset{t\in [0,T]}{\sup}{\left|W_{t}^{\epsilon }-W_{t}\right|}^{2}>\delta \right]=0,$$

*where***W***is the deterministic solution of*

$$\frac{dW_{ij}}{dt}=\bar{G}{\left(W\right)}_{ij}=\underset{\mathrm{decay}}{\underset{︸}{-\kappa W_{ij}}}+\frac{\mu }{\tau }\underset{\mathrm{correlation}}{\underset{︸}{\int_{0}^{\frac{\tau }{\mu }}{\bar{v}}_{i}(s){\bar{v}}_{j}(s)\;ds}}+\underset{\mathrm{noise}}{\underset{︸}{\frac{\sigma^{2}}{2}{\left(L-W\right)}_{ij}^{-1}}},$$

*where*$\bar{v}(t)$*is the*$\frac{\tau }{\mu }$-*periodic attractor of*$\frac{d\bar{v}}{dt}=(W-L)\cdot \bar{v}+u(\mu t)$, *where*$W\in R^{n\times n}$*is supposed to be fixed*.

*Proof* We are going to apply Property 2.3. For p∈]0,1[, consider the space

$$E_{p}=\left\{W\in R^{n\times n}:W\text{ is symmetric},W\geq 0\text{ and }⦀W⦀<lp\right\}.$$

First, since L−W is strictly positive for **W** in $E_{p}$, Assumptions 2.1-2.2 are satisfied on $R^{n}\times E_{p}$. Then, we only need to compute the averaged vector field $\bar{G}$ and show that $E_{p}$ is invariant under the flow of $\bar{G}$.

1. Computation of the averaged vector field $\bar{G}$:

The fast variable is linear, the averaged vector field is given by (10). This reads

$$\bar{G}(W)={\left(\frac{\tau }{\mu }\right)}^{-1}\int_{0}^{\frac{\tau }{\mu }}\int_{x\in R^{n}}G\left(\bar{v}(t)+x,W\right)N_{0,Q}(dx)\;dt,$$

where $N_{v,Q}$ is the probability density function of the Gaussian law with mean **v** and covariance **Q**. And **Q** is the unique solution of (9), with Σ=σId. This leads to $Q=\frac{\sigma^{2}}{2}{\left(L-W\right)}^{-1}$.

Therefore,

$$\begin{array}{rcl} \bar{G}(W) & = & -\kappa W+\frac{\mu }{\tau }\int_{0}^{\frac{\tau }{\mu }}\left(\int_{x\in R^{n}}\left(\bar{v}(t)+x\right)⊗\left(\bar{v}(t)+x\right)N_{0,Q}(dx)\right)\;dt\\ & = & -\kappa W+\frac{\mu }{\tau }\int_{0}^{\frac{\tau }{\mu }}\bar{v}(t)⊗\bar{v}(t)\;dt+\frac{\mu }{\tau }\int_{0}^{\frac{\tau }{\mu }}\left(\bar{v}(t)⊗\underset{Expectation\;of\;N(0,Q)=0}{\underset{︸}{\int_{x\in R^{n}}xN_{0,Q}(dx)}}\right)\;dt\\ & & +\frac{\mu }{\tau }\int_{0}^{\frac{\tau }{\mu }}\left(\underset{Expectation\;of\;N(0,Q)=0}{\underset{︸}{\int_{x\in R^{n}}xN_{0,Q}(dx)}}\;⊗\;\bar{v}(t)\right)\;dt\\ & & +\frac{\mu }{\tau }\int_{0}^{\frac{\tau }{\mu }}\left(\underset{Covariance\;of\;N(0,Q)=Q}{\underset{︸}{\int_{x\in R^{n}}x⊗xN_{0,Q}(dx)}}\right)\;dt\\ & = & -\kappa W+\frac{\mu }{\tau }\int_{0}^{\frac{\tau }{\mu }}\bar{v}(t)⊗\bar{v}(t)\;dt+\frac{\sigma^{2}}{2}{\left(L-W\right)}^{-1}. \end{array}$$

The integral term in the equation above is the correlation matrix of the $\frac{\tau }{\mu }$-periodic function $\bar{\bar{v}}$. To rewrite this term, we define $\bar{v}\in R^{n\times [0,\frac{\tau }{\mu }[}$ such that $\bar{v}(i,t)=\bar{v}{\left(t\right)}_{i}$. $\bar{v}$ can be seen as a matrix gathering the history of $\bar{v}$, *i.e.*, each column of $\bar{v}$ corresponds to the vector $\bar{v}(t)$ for a given $t\in [0,\frac{\tau }{\mu }[$. It turns out

$$\int_{0}^{\frac{\tau }{\mu }}\bar{v}(t)⊗\bar{v}(t)\;dt=\bar{v}\cdot {\bar{v}}^{\prime }.$$

Therefore,

$$\bar{G}(W)=-\kappa W+\frac{\mu }{\tau }\bar{v}\cdot {\bar{v}}^{\prime }+\frac{\sigma^{2}}{2}{\left(L-W\right)}^{-1}.$$

According to the results in Section 2, the solutions of a differential system with such a right-hand side are close to that of the initial system (12). Hence, we focus exclusively on it and try to unveil the properties of its solutions which will be retrospectively extended to those of the initial system (12).

2. Invariance of $E_{p}$ under the flow of (13):

Here we assume that $W(0)\in E_{p}$ and we want to prove that the trajectory of **W** is in $E_{p}$, too. 

(a) Symmetry:

It is clear that each term in $\bar{G}$ is symmetric. Their sum is therefore symmetric and so is W(t).

(b) Inequality W≥0:

The correlation term $\bar{v}\cdot {\bar{v}}^{\prime }$ is a Gramian matrix and is therefore positive. Because L−W is assumed to be positive, therefore, its inverse is also positive. Therefore, if $e_{i}$ is an eigenvector of W≥0 associated with a null eigenvalue, then $e_{i}^{\prime }\cdot \bar{G}(W)\cdot e_{i}\geq 0$. Thus, the trajectories of (13) remain positive.

(c) Inequality ⦀W⦀<lp:

The argument here is that of the inward pointing subspace. We intend to find a condition under which the flow $\bar{G}$ is pointing inward the space {W:⦀W⦀<lp}. Roughly speaking, this will be done by defining a real valued function *g* strictly negative on the subspace and positive outside and then showing that its gradient (or differential) on the border goes in the opposite direction of the flow, *i.e.*, $d_{W}g(\bar{G}(W))<0$ for $W\in g^{-1}(0)$.

For all $x\in C^{n}$ such that ∥x∥=1, define a family of positive numbers $\left(\alpha_{x}\right)$ whose supremum is written $\alpha^{*}$ and a family of functions $\left(g^{x}\right)$ such that

$$g^{x}:\begin{array}{l} R^{n\times n}\to R,\\ J\mapsto {∥J\cdot x∥}^{2}-\alpha_{x}^{2}. \end{array}$$

Observe that the differential of $g^{x}$ at **W** applied to **J** is $dg_{W}^{x}(J)=\frac{1}{2}\langle W\cdot x,J\cdot x\rangle$. For $W\in {g^{x}}^{-1}(0)$, *i.e.*, $∥W\cdot x∥=\alpha_{x}$, compute

$$\begin{array}{rcl} 2dg_{W}^{x}\left(\bar{G}(W)\right) & = & -\kappa \underset{=\alpha_{x}^{2}}{\underset{︸}{\langle W\cdot x,W\cdot x\rangle }}+\frac{\mu }{\tau }\underset{=A}{\underset{︸}{\langle W\cdot x,\bar{v}\cdot {\bar{v}}^{\prime }\cdot x\rangle }}\\ & & +\underset{=B}{\underset{︸}{\langle W\cdot x,\frac{\sigma^{2}}{2}{\left(L-W\right)}^{-1}\cdot x\rangle }}. \end{array}$$

Therefore, for $\alpha^{*}<l$

$$\begin{array}{rcl} \frac{2dg_{W}^{x}(\bar{G}(W))}{\alpha_{x}} & \leq & -\kappa \alpha_{x}+\frac{u_{m}^{2}}{{\left(l-\alpha^{*}\right)}^{2}}+\frac{\sigma^{2}}{2(l-\alpha^{*})}\\ & = & \frac{1}{{\left(l-\alpha^{*}\right)}^{2}}P\left(\alpha^{*}\right)+\kappa \left(\alpha^{*}-\alpha_{x}\right), \end{array}$$

where

(31) $$P(\alpha )=-\kappa \alpha^{3}+2\kappa l\alpha^{2}-\left(\kappa l^{2}+\frac{\sigma^{2}}{2}\right)\alpha +\left(u_{m}^{2}+\frac{l\sigma^{2}}{2}\right).$$

Now write $\alpha^{*}=pl$ with p∈]0,1[. Equation (31) becomes

$$P(p)=-\kappa l^{3}p{\left(1-p\right)}^{2}+\frac{l\sigma^{2}}{2}(1-p)+u_{m}^{2}.$$

When there exists *p* such that P(p)<0 (which corresponds to Assumption 3.1), then their exists a ball of radius *pl* on which the dynamics is pointing inward. It means any matrix **W** whose maximal eigenvalue is $\alpha^{*}=pl$ will see this eigenvalue (and those which are sufficiently close to it, *i.e.*, for which $\alpha^{*}-\alpha_{x}>0$ is sufficiently small) decreasing along the trajectories of the system. Therefore, the space $E_{p}$ is invariant by the flow of the system iff Assumption 3.1 is satisfied.

• Upper bound of *A*:

Applying Cauchy-Schwarz leads to

$$\begin{array}{rcl} \left|A\right| & \leq & ∥W\cdot x∥∥\bar{v}\cdot {\bar{v}}^{\prime }\cdot x∥\leq \alpha_{x}\int_{0}^{\frac{\tau }{\mu }}∥\bar{v}(s)⊗\bar{v}(s)\cdot x∥\;ds\\ & \leq & \alpha_{x}\int_{0}^{\frac{\tau }{\mu }}\left|\langle \bar{v}(s),x\rangle \right|∥\bar{v}(s)∥\;ds\leq \alpha_{x}\int_{0}^{\frac{\tau }{\mu }}{∥\bar{v}(s)∥}^{2}\;ds. \end{array}$$

However, for t≥0

$$\begin{array}{rcl} ∥\bar{v}(t)∥ & \leq & \int_{-\infty }^{t}∥e^{\left(W-L)(t-s\right)}\cdot u(\mu s)∥\;ds\leq u_{m}\int_{-\infty }^{t}e^{\left(\alpha^{*}-l)(t-s\right)}\;ds\\ & \leq & u_{m}e^{(\alpha^{*}-l)t}{\left[\frac{e^{-(\alpha^{*}-l)s}}{l-\alpha^{*}}\right]}_{-\infty }^{t}=\frac{u_{m}}{l-\alpha^{*}}. \end{array}$$

Therefore, $A\leq \frac{\alpha_{x}\tau u_{m}^{2}}{\mu {\left(l-\alpha \right)}^{2}}$.

• Upper bound of *B*:

Observe that for **J** a positive definite matrix whose eigenvalues are the $\lambda_{i}$, then the spectrum of $J^{-1}$ is $\left\{\frac{1}{\lambda_{i}}\right\}$. Therefore, $⦀J^{-1}⦀=\frac{1}{\min(\lambda_{i})}$. Therefore, if J=L−W, then $⦀J^{-1}⦀\leq \frac{1}{l-\alpha^{*}}$.

Using the previous observation and Cauchy-Schwarz leads to

$$|B|\leq \alpha_{x}\frac{\sigma^{2}}{2}⦀{\left(L-W\right)}^{-1}⦀\leq \frac{\alpha_{x}\sigma^{2}}{2(l-\alpha^{*})}.$$

The trajectories of system (13) with the initial condition in $E_{p}$ are defined on $R_{+}$ and remain bounded. Indeed, if $W(0)\in E_{p}$, the connectivity will stay in $E_{p}$, in particular 0<L−W≤L along the trajectories, more precisely L−W is a strictly positive constant since p∈]0,1[. Because $\bar{v}$ is also bounded by $\frac{u_{m}}{l(1-p)}$, $\bar{v}\cdot {\bar{v}}^{\prime }+\frac{\sigma^{2}}{2}{\left(L-W\right)}^{-1}$ is bounded. The right-hand side of system (13) is the sum a bounded term and a linear term multiplied by a negative constant; therefore, the system remains bounded and it cannot explode in finite time: it is defined on $R_{+}$. □

#### B.2.2 An expansion for the correlation term

We first state a useful lemma.

**Lemma B.2***If*$\bar{v}$*is the solution*, *with zero as initial condition*, *of*$\frac{d\bar{v}}{dt}=(W-L)\cdot \bar{v}+u(t)$, *it can be written by the sum below which converges if***W***is in*$E_{p}$*for*p∈]0,1[.

$$\bar{v}=\sum_{k=0}^{+\infty }\frac{W^{k}}{l^{k+1}}\cdot u*g_{l}^{\left(k+1\right)},$$

*where*$g_{l}:t\mapsto le^{-lt}H(t)$.

*Proof* It can be proven as a trivial rewriting of the variation of parameters formula for linear systems. A more general approach, which extends to delayed systems, was developed by Galtier and Touboul [25]; see the first example for the proof of this lemma. □ 

This is useful to find the next result.

**Property B.3***The correlation term can be written*

$$\frac{\mu }{\tau }\bar{v}\cdot {\bar{v}}^{\prime }=\frac{u_{m}^{2}}{l^{2}}\sum_{k,q=0}^{+\infty }\frac{W^{k}}{l^{k}}\cdot C^{k,q}\cdot \frac{{W^{\prime }}^{q}}{l^{q}}.$$

*Proof* We can use Lemma 3.2 with μ≠1 and compute the cross product $\bar{v}\cdot {\bar{v}}^{\prime }$.

Therefore, consider u(μ⋅):t↦u(μt) instead of **u**. A change of variable shows that $\left(u(\mu \cdot )*g_{l}^{\left(k\right)})(t)=\frac{1}{\mu }(u*g_{l}^{\left(k\right)}(\frac{\cdot }{\mu }))(\mu t\right)$. Therefore,

$$\begin{array}{rcl} \frac{\mu }{\tau }{\left\{\bar{v}\cdot {\bar{v}}^{\prime }\right\}}_{ij} & = & \frac{\mu }{\tau }\int_{0}^{\frac{\tau }{\mu }}{\bar{v}}_{i}(t){\bar{v}}_{j}(t)\;dt=\frac{1}{\tau }\int_{0}^{\tau }{\bar{v}}_{i}\left(\frac{s}{\mu }\right){\bar{v}}_{j}\left(\frac{s}{\mu }\right)\;ds\\ & = & \frac{1}{\tau }\int_{0}^{\tau }{\left(\sum_{k=0}^{+\infty }\frac{W^{k}}{l^{k+1}}\cdot \left(u(\mu \cdot )*g_{l}^{\left(k+1\right)}\right)\left(\frac{s}{\mu }\right)\right)}_{i}\\ & & \times {\left(\sum_{q=0}^{+\infty }\frac{W^{q}}{l^{q+1}}\cdot \left(u(\mu \cdot )*g_{l}^{\left(q+1\right)}\right)\left(\frac{s}{\mu }\right)\right)}_{j}\;ds\\ & = & \frac{1}{\tau }\int_{0}^{\tau }{\left(\sum_{k=0}^{+\infty }\frac{W^{k}}{l^{k+1}}\cdot \left(u*\frac{g_{l}^{\left(k+1\right)}(\cdot /\mu )}{\mu }\right)(s)\right)}_{i}\\ & & \times {\left(\sum_{q=0}^{+\infty }\frac{W^{q}}{l^{q+1}}\cdot \left(u*\frac{g_{l}^{\left(q+1\right)}(\cdot /\mu )}{\mu }\right)(s)\right)}_{j}\;ds\\ & = & {\left\{\frac{u_{m}^{2}}{l^{2}}\sum_{k,q=0}^{+\infty }\frac{W^{k}}{l^{k}}\cdot C^{k,q}\cdot \frac{{W^{\prime }}^{q}}{l^{q}}\right\}}_{ij}. \end{array}$$

 □

#### B.2.3 Global stability of the single equilibrium point

**Theorem B.4***If Assumption *3.1 *is verified for*$p\leq \frac{1}{3}$, *then there is a unique equilibrium point in the invariant subset*$E_{p}$*which is globally*, *asymptotically stable*.

*Proof* For this proof, define $F(W)=\frac{u_{m}^{2}}{l^{2}}\sum_{k,q=0}^{+\infty }\frac{W^{k}}{l^{k}}\cdot C^{k,q}\cdot \frac{{W^{\prime }}^{q}}{l^{q}}+\frac{\sigma^{2}}{2}{\left(L-W\right)}^{-1}$.

First, we compute the differential of *F* and show it is a bounded operator. Second, we show it implies the existence and uniqueness of an equilibrium point under some condition. Then, we find an energy for the system which says the fixed point is a global attractor. Finally, we show the stability condition is the same as Assumption 3.1 for $p\leq \frac{1}{3}$. 

1. We compute the differential of each term in *F*: The differential of *F* at **W** is the sum of these two terms.

• Formally write the second term $\bar{v}\cdot {\bar{v}}^{\prime }(W)=\sum_{k,q=0}^{+\infty }\frac{W^{k}}{l^{k}}\cdot C^{k,q}\cdot \frac{{W^{\prime }}^{q}}{l^{q}}$. To find its differential, compute $\bar{v}\cdot {\bar{v}}^{\prime }(W+J)-\bar{v}\cdot {\bar{v}}^{\prime }(W)$ and keep the terms at the first order in **J**. Before computing the whole sum, observe that

$$\begin{array}{rcl} & & {\left(W+J\right)}^{k}\cdot C^{k,q}\cdot {{\left(W+J\right)}^{\prime }}^{q}-W^{k}\cdot C^{k,q}\cdot {W^{\prime }}^{q}\\ & & \;=\sum_{m=0}^{k-1}W^{m}\cdot J\cdot W^{k-1-m}\cdot C^{k,q}\cdot {W^{\prime }}^{q}\\ & & \;+\sum_{m=0}^{q-1}W^{k}\cdot C^{k,q}\cdot {W^{\prime }}^{m}\cdot J^{\prime }\cdot {W^{\prime }}^{q-1-m}+O\left({∥J∥}^{2}\right). \end{array}$$

This leads to

$$\begin{array}{rcl} d\bar{v}\cdot {\bar{v}}_{W}^{\prime }(J) & = & \frac{1}{l}\sum_{k,q=0}^{+\infty }(\sum_{m=0}^{k-1}\frac{W^{m}}{l^{m}}\cdot J\cdot \frac{W^{k-1-m}}{l^{k-1-m}}\cdot C^{k,q}\cdot \frac{{W^{\prime }}^{q}}{l^{q}}\\ & & +\sum_{l=0}^{q-1}\frac{W^{k}}{l^{k}}\cdot C^{k,q}\cdot \frac{{W^{\prime }}^{m}}{l^{m}}\cdot J^{\prime }\cdot \frac{{W^{\prime }}^{q-1-m}}{l^{q-1-m}}). \end{array}$$

• Write $Q:W\mapsto {\left(L-W\right)}^{-1}$. We can write (L−W)⋅Q(W)=Id and use the chain rule to compute the differential of *Q* at **W**, which gives $-J\cdot Q(W)+(L-W)\cdot dQ_{W}(J)=0$. Therefore,

$$dQ_{W}(J)={\left(L-W\right)}^{-1}\cdot J\cdot {\left(L-W\right)}^{-1}.$$

2. We want to compute the norm of ${∥dF_{W}(J)∥}_{2}$ for ${∥J∥}_{2}=1$. First, observe that for three square matrices **A**, **B**, and **C**,

$$\begin{array}{rcl} {∥A\cdot B\cdot C∥}_{2}^{2} & = & \sum_{i,j=1}^{n}B_{ij}^{2}{∥A\cdot (e_{i}⊗e_{j})\cdot C∥}_{2}^{2}\leq \sum_{i,j=1}^{n}B_{ij}^{2}{∥A\cdot e_{i}∥}_{2}^{2}{∥C\cdot e_{j}∥}_{2}^{2}\\ & \leq & \sum_{i,j=1}^{n}B_{ij}^{2}⦀A{⦀}^{2}⦀C{⦀}^{2}, \end{array}$$

for $e_{i}$ the vectors of the canonical basis of $R^{n}$. This leads to ${∥A\cdot B\cdot C∥}_{2}\leq {∥B∥}_{2}⦀A⦀⦀C⦀$. Therefore, because $⦀A⦀\leq {∥A∥}_{2}$,

$$\begin{array}{rcl} {∥\frac{W^{m}}{l^{m}}\cdot J\cdot \frac{W^{k-1-m}}{l^{k-1-m}}\cdot C^{k,q}\cdot \frac{{W^{\prime }}^{q}}{l^{q}}∥}_{2} & \leq & \frac{⦀W{⦀}^{m}}{l^{m}}{∥\frac{W^{k-1-m}}{l^{k-1-m}}\cdot C^{k,q}\cdot \frac{{W^{\prime }}^{q}}{l^{q}}∥}_{2}\\ & \leq & u_{m}^{2}\frac{⦀W{⦀}^{k-1}}{l^{k-1}}\frac{⦀W{⦀}^{q}}{l^{q}}. \end{array}$$

Therefore,

$$\begin{array}{rcl} {∥dF_{W}(J)∥}_{2} & \leq & \frac{u_{m}^{2}}{l^{3}}\sum_{k,q=0}^{+\infty }\left(k\frac{⦀W{⦀}^{k-1}}{l^{k-1}}\frac{⦀W{⦀}^{q}}{l^{q}}+q\frac{⦀W{⦀}^{k}}{l^{k}}\frac{⦀W{⦀}^{q-1}}{l^{q-1}}\right)\\ & & +\frac{\sigma^{2}}{2}⦀{\left(L-W\right)}^{-1}{⦀}^{2}\\ & \leq & \frac{2u_{m}^{2}}{l^{3}}\left(\sum_{k=0}^{+\infty }kp^{k-1}\right)\left(\sum_{q=0}^{+\infty }p^{q}\right)+\frac{\sigma^{2}}{2}⦀{\left(L-W\right)}^{-1}{⦀}^{2}\\ & \leq & \frac{2u_{m}^{2}}{l^{3}{\left(1-p\right)}^{3}}+\frac{\sigma^{2}}{2l^{2}{\left(1-p\right)}^{2}}. \end{array}$$

This inequality is true for all **J** with ${∥J∥}_{2}=1$; therefore, it is also true for the operator norm

$$⦀dF_{W}⦀\leq \frac{2u_{m}^{2}}{l^{3}{\left(1-p\right)}^{3}}+\frac{\sigma^{2}}{2l^{2}{\left(1-p\right)}^{2}}.$$

Therefore, *F* is a *k*-Lipschitz operator where $k=\frac{2u_{m}^{2}}{l^{3}{\left(1-p\right)}^{3}}+\frac{\sigma^{2}}{2l^{2}{\left(1-p\right)}^{2}}$. This means ${∥F(W)-F(J)∥}_{2}\leq k{∥W-J∥}_{2}$.

3. The equilibrium points of system (15) necessarily verify the equation $W=\frac{1}{\kappa }F(W)$. If

(32) $$\frac{2u_{m}^{2}}{{\left(1-p\right)}^{3}}+\frac{l\sigma^{2}}{2{\left(1-p\right)}^{2}}<\kappa l^{3},$$

then $\frac{1}{\kappa }F$ is a contraction map from $E_{p}$ to itself. Therefore, the Banach fixed point theorem says that there is a unique fixed point which we write $W^{*}$.

4. We now show that, under assumption (32), $W\mapsto {∥W-W^{*}∥}_{2}^{2}$ is an energy function for the system $\frac{dW}{dt^{\prime }}=-W+\frac{1}{\kappa }F(W)$ (which is a rescaled version of system (15)).

Indeed, compute the derivative of this energy along the trajectories of the system

$$\begin{array}{rcl} 2\frac{d}{dt}{∥W(t)-W^{*}∥}_{2}^{2} & = & \langle W-W^{*},-W+\frac{1}{\kappa }F(W)\rangle \\ & = & -\langle W-W^{*},W-W^{*}\rangle +\langle W-W^{*},\frac{1}{\kappa }F(W)-W^{*}\rangle \\ & = & -{∥W-W^{*}∥}_{2}^{2}+\langle W-W^{*},\frac{1}{\kappa }F(W)-\frac{1}{\kappa }F\left(W^{*}\right)\rangle \\ & \leq & -{∥W-W^{*}∥}_{2}^{2}+{∥W-W^{*}∥}_{2}{∥\frac{1}{\kappa }F(W)-\frac{1}{\kappa }F\left(W^{*}\right)∥}_{2}\\ & \leq & \frac{1}{\kappa l^{3}}\left(\frac{2u_{m}^{2}}{{\left(1-p\right)}^{3}}+\frac{l\sigma^{2}}{2{\left(1-p\right)}^{2}}-\kappa l^{3}\right){∥W-W^{*}∥}_{2}^{2}\leq 0. \end{array}$$

The energy is lower-bounded, takes its minimum for $W=W^{*}$ and the decreases along the trajectories of the system. Therefore, $W^{*}$ is globally asymptotically stable if assumption (32) is verified.

5. Observe that if Assumption 3.1 is verified for $p\leq \frac{1}{3}$, then $\frac{1}{1-p}<\frac{2}{1-p}\leq \frac{1}{p}$. Therefore, Assumption 3.1 implies that (32) is also true. This concludes the proof. □

#### B.2.4 Explicit expansion of the equilibrium point

Recall the notations $\tilde{p}=\frac{u_{m}^{2}}{\kappa l^{3}}+\frac{\sigma^{2}}{2\kappa l^{2}}$ and $\lambda =\frac{\sigma^{2}l}{2u_{m}^{2}}$.

**Theorem B.5**

$$\begin{array}{rcl} W & = & \frac{\tilde{p}l}{1+\lambda }\left(\lambda +C^{0,0}\right)+\frac{{\tilde{p}}^{2}l}{{\left(1+\lambda \right)}^{2}}(\lambda^{2}+\lambda \left(C^{0,0}+C^{1,0}+C^{0,1}\right)\\ & & +C^{0,0}\cdot C^{1,0}+C^{0,1}\cdot C^{0,0})+O\left({\tilde{p}}^{3}\right). \end{array}$$

Actually, it is possible to compute recursively the *n*th term of the expansion above, although their complexity explodes.

*Proof* Define $p^{*}$ the smallest value in ]0,1[ such that Assumption 3.1 is valid. This implies

$$p^{*}\left({\left(1-p^{*}\right)}^{2}+\frac{\sigma^{2}}{2\kappa l^{2}}\right)=\frac{u_{m}^{2}}{\kappa l^{3}}+\frac{\sigma^{2}}{2\kappa l^{2}}.$$

The weak connectivity index $\tilde{p}$ controls the ratio of the connection over the strength of intrinsic dynamics. Indeed, these two variables are of the same order because

$$\frac{p^{*}}{\tilde{p}}=\frac{1}{{\left(1-p^{*}\right)}^{2}+\frac{\sigma^{2}}{2\kappa l^{2}}}=O_{\tilde{p}\to 0}(1).$$

We want to approximate the equilibrium $W^{*}$, *i.e.*, the solution of $\bar{G}(W^{*})=0$, in the regime $\tilde{p}\ll 1$. Define $\Omega =\frac{W}{\tilde{p}l}$ such that ⦀Ω⦀=O(1). We abusively write $\bar{G}(\Omega )=\bar{G}(\tilde{p}l\Omega )$ such that

$$\bar{G}(\Omega )=-\tilde{p}l\kappa \Omega +\frac{u_{m}^{2}}{l^{2}}\sum_{k,q=0}^{+\infty }{\left(\tilde{p}\Omega \right)}^{k}\cdot C^{k,q}\cdot {\left(\tilde{p}\Omega \right)}^{q}+\frac{\sigma^{2}}{2l}\sum_{k=0}^{+\infty }{\left(\tilde{p}\Omega \right)}^{k}.$$

Recalling $\lambda =\frac{\sigma^{2}l}{2u_{m}^{2}}$ leads to

$$\bar{G}(\Omega )=\left(\frac{u_{m}^{2}}{l^{2}}+\frac{\sigma^{2}}{2l}\right)\left(-\Omega +\frac{1}{1+\lambda }\sum_{k,q=0}^{+\infty }{\left(\tilde{p}\Omega \right)}^{k}\cdot C^{k,q}\cdot {\left(\tilde{p}\Omega \right)}^{q}+\frac{\lambda }{1+\lambda }\sum_{k=0}^{+\infty }{\left(\tilde{p}\Omega \right)}^{k}\right).$$

Now, we write a candidate $\Omega^{\left(m\right)}=\sum_{a=0}^{m}{\tilde{p}}^{a}\Omega_{a}$, then we chose the terms $\Omega_{a}=O(1)$ so that the first *m*th orders in $\bar{G}(\Omega^{\left(m\right)})$ vanish. This implies that $∥\bar{G}(\Omega^{*})-\bar{G}(\Omega^{\left(m\right)})∥=O({\tilde{p}}^{m+1})$, where $\Omega^{*}=\frac{W^{*}}{\tilde{p}l}$. Then, we use the fact that the minimal absolute value of the eigenvalues of $\bar{G}$ is larger than $\kappa -(\frac{2u_{m}^{2}}{l^{3}{\left(1-p\right)}^{3}}+\frac{\sigma^{2}}{2l^{2}{\left(1-p\right)}^{2}})>0$. Indeed, it means

$$∥W^{*}-W^{\left(m\right)}∥<\frac{1}{\kappa -(\frac{2u_{m}^{2}}{l^{3}{\left(1-p\right)}^{3}}+\frac{\sigma^{2}}{2l^{2}{\left(1-p\right)}^{2}})}O\left(p^{m+1}\right)<\frac{1}{\kappa -(\frac{2u_{m}^{2}}{l^{3}}+\frac{\sigma^{2}}{2l^{2}})}O\left(p^{m+1}\right),$$

*i.e.*, $\Omega^{\left(m\right)}=\Omega^{*}+O({\tilde{p}}^{m+1})$.

Thus, we need to find the $\Omega_{a}$ such that the first *m*th orders in $\bar{G}(\Omega^{\left(m\right)})$ vanish. Therefore, we need to expand all the terms in $\bar{G}(\Omega )$. The first term is obvious. In the following, we write the second term F(Ω) associated to the correlations and look for an explicit expression of the $F_{a}$ such that $F(\Omega )=\sum_{a=0}^{+\infty }{\tilde{p}}^{a}F_{a}$. Second, we write the third term Q(Ω) associated to the noise and look for an explicit expression of the $Q_{a}$ such that $Q(\Omega )=\sum_{a=0}^{+\infty }{\tilde{p}}^{a}Q_{a}$. 

• Finding the $F_{a}$:

First, observe that

(33) $$\Omega^{q}=\sum_{i=0}^{+\infty }{\tilde{p}}^{i}\sum_{\eta \in N^{q},\sum_{k}\eta_{k}=i}\Omega_{\eta_{1}}\cdot \Omega_{\eta_{2}}\cdot \ldots \cdot \Omega_{\eta_{q}}.$$

This leads to

$$\begin{array}{rcl} F(\Omega ) & = & \frac{1}{1+\lambda }\sum_{k,q=0}^{+\infty }\overset{+\infty }{\underset{j\leq i}{\sum_{i,j=0}}}{\tilde{p}}^{i+k+q}\underset{\theta \in N^{i-j},\sum_{n}\theta_{n}=q}{\sum_{\eta \in N^{j},\sum_{n}\eta_{n}=k}}\Omega_{\eta_{1}}\cdot \ldots \cdot \Omega_{\eta_{j}}\\ & & \times C^{j,i-j}\cdot \Omega_{\theta_{1}}^{\prime }\cdot \ldots \cdot \Omega_{\theta_{i-j}}^{\prime }. \end{array}$$

The *a*th term in the power expansion in $\tilde{p}$ verifies a=i+k+q. More precisely, this reads

$$F_{a}=\frac{1}{1+\lambda }\overset{+\infty }{\underset{a=i+k+q}{\sum_{k,q,i=0}}}\sum_{j=0}^{i}\underset{\theta \in N^{i-j},\sum_{n}\theta_{n}=q}{\sum_{\eta \in N^{j},\sum_{n}\eta_{n}=k}}\Omega_{\eta_{1}}\cdot \ldots \cdot \Omega_{\eta_{j}}\cdot C^{j,i-j}\cdot \Omega_{\theta_{1}}^{\prime }\cdot \ldots \cdot \Omega_{\theta_{i-j}}^{\prime }.$$

This equation is scary but it reduces to simple expressions for small a∈N.

• Finding the $Q_{a}$:

Using equation (33) leads to

$$Q(\Omega )=\frac{\lambda }{1+\lambda }\sum_{i,q=0}^{+\infty }{\tilde{p}}^{i+q}\sum_{\eta \in N^{q},\sum_{k}\eta_{k}=i}\Omega_{\eta_{1}}\cdot \Omega_{\eta_{2}}\cdot \ldots \cdot \Omega_{\eta_{q}}.$$

The *a*th term in the power expansion in $\tilde{p}$ verifies a=i+q. More precisely, this reads

$$Q_{a}=\frac{\lambda }{1+\lambda }\overset{+\infty }{\underset{a=i+q}{\sum_{q,i=0}}}{\tilde{p}}^{i+q}\sum_{\eta \in N^{q},\sum_{k}\eta_{k}=i}\Omega_{\eta_{1}}\cdot \Omega_{\eta_{2}}\cdot \ldots \cdot \Omega_{\eta_{q}}.$$

Therefore,

$$\begin{array}{ccc} a & (1+\frac{1}{\lambda })Q_{a} & (1+\lambda )F_{a}\\ 0 & Id & C^{0,0}\\ 1 & \Omega_{0} & \Omega_{0}\cdot C^{1,0}+C^{0,1}\cdot \Omega_{0}\\ 2 & \Omega_{0}^{2}+\Omega_{1} & \Omega_{0}^{2}\cdot C^{2,0}+C^{0,2}\cdot \Omega_{0}^{2}+\Omega_{0}\cdot C^{1,1}\cdot \Omega_{0}+\Omega_{1}\cdot C^{1,0}+C^{0,1}\cdot \Omega_{1} \end{array}$$

Therefore, it is easy to compute $\Omega_{a}=F_{a}+Q_{a}$ for a∈N. By definition $W=\tilde{p}l\Omega =\tilde{p}l(F+Q)$, which leads to the result. □

### B.3 Trace learning with damped oscillators and dynamic synapses

**Theorem B.6***If Assumption *3.1 *is verified for*p∈]0,1[, *then system* (16) *is asymptotically well posed in probability and the connectivity matrix*$W^{\epsilon }$, *solution of system* (16), *converges to***W***in the sense that for all*δ,T>0,

$$\lim_{\epsilon \to 0}^{\mu }P\left[\underset{t\in [0,T]}{\sup}{\left|W_{t}^{\epsilon }-W_{t}\right|}^{2}>\delta \right]=0,$$

*where***W***is the deterministic solution of*

$$\frac{dW_{ij}}{dt}=\bar{G}{\left(W\right)}_{ij}=\underset{\mathrm{decay}}{\underset{︸}{-\kappa W_{ij}}}+\frac{\mu }{\tau }\underset{\mathrm{correlation}}{\underset{︸}{\int_{0}^{\frac{\tau }{\mu }}({\bar{v}}_{i}*g_{\beta })(s)({\bar{v}}_{j}*g_{\beta })(s)\;ds}}+\underset{\mathrm{noise}}{\underset{︸}{Q_{22}}},$$

*where*$\bar{v}(t)$*is the*$\frac{\tau }{\mu }$-*periodic attractor of*$\frac{d\bar{v}}{dt}=(W-L)\cdot \bar{v}*g_{\beta }+u(\mu t)$, *where*$W\in R^{n\times n}$*is supposed to be fixed*. *And*$Q_{22}$*is a noise matrix described below*.

*Proof* First, let us recall the instantaneous reformulation of (16)

$$\left\{ \begin{array}{c} d\left(\begin{array}{c} v^{\epsilon }\\ z^{\epsilon } \end{array}\right)=\frac{1}{\epsilon_{1}}\left[\left(\begin{array}{cc} 0 & W-L\\ \beta & -\beta \end{array}\right)\left(\begin{array}{c} v^{\epsilon }\\ z^{\epsilon } \end{array}\right)+\left(\begin{array}{c} u(\frac{t}{\epsilon_{2}})\\ 0 \end{array}\right)\right]\;dt+\left(\begin{array}{c} \frac{\sigma }{\sqrt{\epsilon_{1}}}\;dB(t)\\ \frac{\sigma_{z}}{\sqrt{\epsilon_{1}}}\;dB(t) \end{array}\right),\\ \frac{dW^{\epsilon }}{dt}=-\kappa W^{\epsilon }+z^{\epsilon }⊗z^{\epsilon }. \end{array}\right.$$

Starting from this system, the structure of the proof of Theorem 3.1 remains unchanged. The correlation term is to be replaced by $\frac{\mu }{\tau }\bar{v}\cdot G_{\beta }\cdot G_{\beta }^{\prime }\cdot {\bar{v}}^{\prime }$. The noise term we are looking for is $Q_{22}$ in the Lyapunov equation (see (9)) below

$$\left(\begin{array}{cc} 0 & W-L\\ \beta & -\beta \end{array}\right)\cdot \left(\begin{array}{cc} Q_{11} & Q_{12}^{\prime }\\ Q_{12} & Q_{22} \end{array}\right)+\left(\begin{array}{cc} Q_{11} & Q_{12}^{\prime }\\ Q_{12} & Q_{22} \end{array}\right)\cdot \left(\begin{array}{cc} 0 & \beta \\ W^{\prime }-L & -\beta \end{array}\right)+\left(\begin{array}{cc} \sigma^{2} & 0\\ 0 & \sigma_{z}^{2} \end{array}\right)=0.$$

Because the learning rule is symmetric, then the space of symmetric matrices is invariant and we can restrict this section to the symmetric case. It is easy to show that this Lyapunov equation has a unique solution, because the sum of two eigenvalues of the drift matrix is never null (provided **W** stays in $E_{p}$). This leads to the system

$$\left\{ \begin{array}{cc} (W-L)\cdot Q_{12}+Q_{12}^{\prime }\cdot (W-L)+\sigma^{2}=0, & \left(a\right)\\ \beta (Q_{11}-Q_{12})+Q_{22}\cdot (W-L)=0, & \left(b\right)\\ Q_{22}=\frac{Q_{12}+Q_{12}^{\prime }}{2}+\frac{\sigma_{z}^{2}}{2\beta }I_{d}. & \left(c\right) \end{array}\right.$$

One solution of equation (a) is $Q_{12}=\frac{\sigma^{2}}{2}{\left(L-W\right)}^{-1}$. Equation (c) defines $Q_{22}$ properly. Indeed, because **W** is symmetric, so is $Q_{12}$ and $Q_{22}=\frac{\sigma^{2}}{2}{\left(L-W\right)}^{-1}+\frac{\sigma_{z}^{2}}{2\beta }I_{d}$. Similarly, equation (b) defines $Q_{11}$ but it remains to be checked that this definition is that of a symmetric matrix. In fact, it works because **W** is assumed symmetric and the noise has no off-diagonal terms. Indeed, in this case, $Q_{11}=\frac{\sigma^{2}}{2}{\left(L-W\right)}^{-1}+\frac{\sigma^{2}}{2\beta }+\frac{\sigma_{z}^{2}}{2\beta^{2}}(L-W)$. This solution is thus the unique solution of the Lyapunov equation.

Therefore,

$$\bar{G}(W)=-\kappa W+\frac{\mu }{\tau }\bar{v}\cdot G_{\beta }\cdot G_{\beta }^{\prime }\cdot {\bar{v}}^{\prime }+\frac{\sigma^{2}}{2}{\left(L-W\right)}^{-1}+\frac{\sigma_{z}^{2}}{2\beta }I_{d}.$$

Thus, this application of Theorem 2.2 to the instantaneous system with $\sigma_{z}\neq 0$, leads to the previous averaged equation. To recover the initial case (16), we can let $\sigma_{z}\to 0$. We see that the function $\bar{G}$ tends to

$${\bar{G}}^{*}(W)=-\kappa W+\frac{\mu }{\tau }\bar{v}\cdot G_{\beta }\cdot G_{\beta }^{\prime }\cdot {\bar{v}}^{\prime }+\frac{\sigma^{2}}{2}{\left(L-W\right)}^{-1},$$

which we will rewrite $\bar{G}$ for simplicity in the following. Thus, this definition of $\bar{G}$ defines the averaged system for the original equation (16).

In the derivation of the condition under which ⦀W⦀ remain smaller than *lp*, the upper bound of the term *A* changes as follows. Define $M\in R_{+}$ so that $∥\bar{v}(t)∥\leq M$ for all t>0. Because we assume $\bar{v}(R_{-})=0$, the variation of parameters formula for linear retarded differential equations with constant coefficients (see Chapter 6 of [34]) reads $\bar{v}(t)=\int_{0}^{t}U(t-s)\cdot u(\mu s)\;ds$ where the resolvent **U** is the solution of $\dot{U}=(W-L)\cdot (U*g)$. We use Corollary 1.1 of Chapter 6 of [34], which is based on Grönwall’s lemma, to claim that $∥U(t-s)∥\leq e^{\left(t-s)(\alpha^{*}-l\right)}$. Therefore,

$$∥\bar{v}(t)∥\leq \int_{-\infty }^{t}∥U(t-s)∥∥u(\mu s)∥\;ds\leq u_{m}{\left[\frac{e^{\left(t-s)(\alpha^{*}-l\right)}}{l-\alpha^{*}}\right]}_{-\infty }^{t}\leq \frac{u_{m}}{l-\alpha^{*}}=M.$$

Then, we used Young’s inequality for convolution to get ${∥(\bar{v}*g)(t)∥}_{2}\leq {∥\bar{v}∥}_{2}{∥g∥}_{1}={∥\bar{v}∥}_{2}$.

Therefore, the upper bound of *A* remains unchanged.

Therefore, the polynomial *P* remains the same and Assumption 3.1 is still relevant to this problem. □

**Lemma B.7***If*$\bar{v}$*is the solution*, *with zero as initial condition*, *of*$\frac{d\bar{v}}{dt}=(W-L)\cdot \bar{v}*g_{\beta }+u(t)$, *it can be written by the sum below which converges if***W***is in*$E_{p}$*for*p∈]0,1[.

$$\bar{v}=\sum_{k=0}^{+\infty }\frac{W^{k}}{l^{k+1}}\cdot u\cdot \tilde{W}\cdot {\tilde{V}}^{k},$$

*where*$\tilde{W}$*and*$\tilde{V}$*are convolution operators respectively generated by the functions*$\tilde{w}$*and*$\tilde{v}$*detailed below*

$$\begin{array}{rcl} \tilde{w}:t & \mapsto & \frac{l}{2\Delta }\left((1+\Delta )e^{-\frac{\beta }{2}(1-\Delta )t}-(1-\Delta )e^{-\frac{\beta }{2}(1+\Delta )t}\right)H(t),\\ \tilde{v}:t & \mapsto & \frac{l}{\Delta }\left(e^{-\frac{\beta }{2}(1-\Delta )t}-e^{-\frac{\beta }{2}(1+\Delta )t}\right)H(t), \end{array}$$

*whereHis the Heaviside function*, $\Delta =\sqrt{1-\frac{4l}{\beta }}$. *If* Δ *is a pure imaginary number*, *the expression above still holds with the hyperbolic functionsshandchbeing turned into classical trigonometric functions* sin *and* cos *and* Δ *being replaced by its modulus*.

*If***W***is in*$E_{p}$*for*p∈]0,1[, *then this expansion converges*.

*Proof* See the second example of [25]. □ 

Using Lemma C.3, on can redefine

$${\tilde{C}}^{k,q}=\frac{1}{u_{m}^{2}\tau {∥v∥}_{1}^{k+q+2}}u\cdot V^{k+1}\cdot {\left(u\cdot V^{q+1}\right)}^{\prime },$$

where V is the convolution operator generated by $v(t)=\frac{l}{\mu \Delta }(e^{-\frac{\beta }{2\mu }(1-\Delta )t}-e^{-\frac{\beta }{2\mu }(1+\Delta )t})H(t)$ (see Appendix C for details). Observe that applying Young’s inequality for convolutions leads to ${∥{\tilde{C}}^{k,q}∥}_{2}\leq 1$.

Therefore, we can rewrite Theorem 3.3 into

**Theorem B.8**

$$\frac{\mu }{\tau }\bar{v}\cdot G_{\beta }\cdot G_{\beta }^{\prime }\cdot {\bar{v}}^{\prime }=\frac{u_{m}^{2}{∥v∥}_{1}^{2}}{l^{2}}\sum_{k,q=0}^{+\infty }\frac{W^{k}}{{\left(l/{∥v∥}_{1}\right)}^{k}}\cdot {\tilde{C}}^{k,q}\cdot \frac{{W^{\prime }}^{q}}{{\left(l/{∥v∥}_{1}\right)}^{q}}.$$

*Proof* Similar to that of Theorem 3.3. □

**Theorem B.9***If Assumption *3.1 *is verified for*$p\leq \frac{1}{2{∥v∥}_{1}^{3}+1}$, *there is a unique equilibrium point which is globally*, *asymptotically stable*.

*Proof* Similar to the proof of Theorem B.4. □

With the same definitions for $\tilde{p}=\frac{u_{m}^{2}}{\kappa l^{3}}+\frac{\sigma^{2}}{2\kappa l^{2}}$ and $\lambda =\frac{\sigma^{2}l}{2u_{m}^{2}}$, we can show

**Theorem B.10***The weakly connected expansion of the equilibrium point is*

$$\begin{array}{rcl} W^{*} & = & \frac{\tilde{p}l}{1+\lambda }\left(\lambda +{∥v∥}_{1}^{2}{\tilde{C}}^{0,0}\right)\\ & & +\frac{{\tilde{p}}^{2}{∥v∥}_{1}l}{{\left(1+\lambda \right)}^{2}}(\frac{\lambda^{2}}{{∥v∥}_{1}}+\lambda \left({∥v∥}_{1}{\tilde{C}}^{0,0}+{∥v∥}_{1}^{2}{\tilde{C}}^{1,0}+{∥v∥}_{1}^{2}{\tilde{C}}^{0,1}\right)\\ & & +{∥v∥}_{1}^{4}{\tilde{C}}^{0,0}\cdot {\tilde{C}}^{1,0}+{∥v∥}_{1}^{4}{\tilde{C}}^{0,1}\cdot {\tilde{C}}^{0,0})+O\left({\tilde{p}}^{3}{∥v∥}_{1}^{2}\right). \end{array}$$

*Proof* Define $\Omega =\frac{W}{\tilde{p}l}$ so that

$$\begin{array}{rcl} \bar{G}(\Omega ) & = & \left(\frac{u_{m}^{2}}{l^{2}}+\frac{\sigma^{2}}{2l}\right)(-\Omega +\frac{{∥v∥}_{1}^{2}}{1+\lambda }\sum_{k,q=0}^{+\infty }{\left(\tilde{p}{∥v∥}_{1}\Omega \right)}^{k}\cdot {\tilde{C}}^{k,q}\cdot {\left(\tilde{p}{∥v∥}_{1}\Omega \right)}^{q}\\ & & +\frac{\lambda }{1+\lambda }\sum_{k=0}^{+\infty }{\left(\tilde{p}\Omega \right)}^{k}). \end{array}$$

So, the expansion will be in orders of $\tilde{p}{∥v∥}_{1}$ with ${∥v∥}_{1}\geq 1$.

Therefore,

$$\begin{array}{ccc} a & (1+\frac{1}{\lambda })Q_{a} & \frac{1+\lambda }{{∥v∥}_{1}^{2}}F_{a}\\ 0 & Id & {\tilde{C}}^{0,0}\\ 1 & \frac{\Omega_{0}}{{∥v∥}_{1}} & \Omega_{0}\cdot {\tilde{C}}^{1,0}+{\tilde{C}}^{0,1}\cdot \Omega_{0}\\ 2 & \frac{\Omega_{0}^{2}+\Omega_{1}}{{∥v∥}_{1}^{2}} & \Omega_{0}^{2}\cdot {\tilde{C}}^{2,0}+{\tilde{C}}^{0,2}\cdot \Omega_{0}^{2}+\Omega_{0}\cdot {\tilde{C}}^{1,1}\cdot \Omega_{0}+\Omega_{1}\cdot {\tilde{C}}^{1,0}+{\tilde{C}}^{0,1}\cdot \Omega_{1} \end{array}$$

Actually, it is possible to compute recursively the *n*th terms, although their complexity explodes. Therefore, it is easy to compute $\Omega_{a}=F_{a}+Q_{a}$ for a∈N. By definition $W=\tilde{p}l\Omega =\tilde{p}l(F+Q)$, which leads to the result. □

### B.4 STDP learning with linear neurons and correlated noise

Consider the following *n*-dimensional stochastic differential system:

$$\left\{ \begin{array}{c} dv^{\epsilon }=\frac{1}{\epsilon_{1}}(-L\cdot v^{\epsilon }+W\cdot v^{\epsilon }+u(\frac{t}{\epsilon_{2}}))\;dt+\frac{1}{\sqrt{\epsilon_{1}}}\Sigma \cdot dB(t),\\ \frac{dW^{\epsilon }}{dt}=G(v^{\epsilon },W^{\epsilon })=-\kappa W^{\epsilon }+a_{+}v^{\epsilon }⊗(v^{\epsilon }*g_{\gamma })-a_{-}(v^{\epsilon }*g_{\gamma })⊗v^{\epsilon }, \end{array}\right.$$

where **u** is a continuous input in $R^{n}$, $l,\epsilon_{1},\epsilon_{2},\kappa \in R_{+}$, $a_{+},a_{-}\in R$, $\Sigma \in R^{n\times n}$ and B(t) is *n*-dimensional Brownian noise, and for all γ>0, $g_{\gamma }:t\mapsto \gamma e^{-\gamma t}H(t)$ where *H* is the Heaviside function. Recall the well-posedness Assumption 3.2

**Assumption B.1** There exists p∈]0,1[ such that

$$\frac{\left|a_{+}|+|a_{-}\right|}{p(1-p)}\left(\frac{s^{2}\gamma }{2(1+\gamma /l-p)}+\frac{u_{m}^{2}}{\left(1-p\right)}\right)<\kappa l^{3}.$$

**Theorem B.11***If Assumption *3.2 *is verified for*p∈]0,1[, *then system* (17) *is asymptotically well posed in probability and the connectivity matrix*$W^{\epsilon }$, *the solution of system* (17), *converges to***W***in the sense that for all*δ,T>0,

$$\lim_{\epsilon \to 0}^{\mu }P\left[\underset{t\in [0,T]}{\sup}{\left|W_{t}^{\epsilon }-W_{t}\right|}^{2}>\delta \right]=0,$$

*where***W***is the deterministic solution of*

$$\begin{array}{rcl} \frac{dW_{ij}}{dt} & = & \bar{G}{\left(W\right)}_{ij}\\ & = & \underset{\mathrm{decay}}{\underset{︸}{-\kappa W_{ij}}}+\frac{\mu }{\tau }\underset{\mathrm{correlation}}{\underset{︸}{\int_{0}^{\frac{\tau }{\mu }}a_{+}{\bar{v}}_{i}(s)({\bar{v}}_{j}*g_{\gamma })(s)-a_{-}({\bar{v}}_{i}*g_{\gamma })(s){\bar{v}}_{j}(s)\;ds}}+\underset{\mathrm{noise}}{\underset{︸}{Q_{12}}}, \end{array}$$

*where*$\bar{v}(t)$*is the*$\frac{\tau }{\mu }$-*periodic attractor of*$\frac{d\bar{v}}{dt}=(W-L)\cdot \bar{v}+u(\mu t)$, *where*$W\in R^{n\times n}$*is supposed to be fixed*. *And*$Q_{12}$*is described below*.

*Proof* We recall the instantaneous reformulation of the original system (17)

$$\left\{ \begin{array}{c} d\left(\begin{array}{c} v^{\epsilon }\\ z^{\epsilon } \end{array}\right)=\frac{1}{\epsilon_{1}}\left[\left(\begin{array}{cc} W-L & 0\\ \gamma & -\gamma \end{array}\right)\left(\begin{array}{c} v^{\epsilon }\\ z^{\epsilon } \end{array}\right)+\left(\begin{array}{c} u(\frac{t}{\epsilon_{2}})\\ 0 \end{array}\right)\right]\;dt+\left(\begin{array}{c} \frac{\sigma }{\sqrt{\epsilon_{1}}}\;dB(t)\\ \frac{\sigma_{z}}{\sqrt{\epsilon_{1}}}\;dB(t) \end{array}\right),\\ \frac{dW^{\epsilon }}{dt}=-\kappa W^{\epsilon }+a_{+}v^{\epsilon }⊗z^{\epsilon }-a_{-}z^{\epsilon }⊗v^{\epsilon }. \end{array}\right.$$

With this linear expression, the structure of the proof of Theorem 3.1 remains unchanged. The correlation term is to be replaced by $\frac{\mu }{\tau }(a_{+}\bar{v}\cdot G_{\gamma }^{\prime }\cdot {\bar{v}}^{\prime }+a_{-}\bar{v}\cdot G_{\gamma }\cdot {\bar{v}}^{\prime })$. The noise term we are looking for is $Q_{12}$ in the Lyapunov equation (see (9)) below

$$\begin{array}{rcl} & & \left(\begin{array}{cc} W-L & 0\\ \gamma & -\gamma \end{array}\right)\cdot \left(\begin{array}{cc} Q_{11} & Q_{12}^{\prime }\\ Q_{12} & Q_{22} \end{array}\right)\\ & & \;+\left(\begin{array}{cc} Q_{11} & Q_{12}^{\prime }\\ Q_{12} & Q_{22} \end{array}\right)\cdot \left(\begin{array}{cc} W^{\prime }-L & \gamma \\ 0 & -\gamma \end{array}\right)+\left(\begin{array}{cc} \Sigma \cdot \Sigma^{\prime } & 0\\ 0 & \frac{\sigma_{z}^{2}}{2}I_{d} \end{array}\right)=0. \end{array}$$

This leads to the system

(34) $$\left\{ \begin{array}{cc} (W-L)\cdot Q_{11}+Q_{11}\cdot (W^{\prime }-L)+\Sigma \cdot \Sigma^{\prime }=0, & \left(a\right)\\ \gamma (Q_{11}-Q_{12})+Q_{12}\cdot (W^{\prime }-L)=0, & \left(b\right)\\ Q_{22}=\frac{Q_{12}+Q_{12}^{\prime }}{2}+\frac{\sigma_{z}^{2}}{2}I_{d}. & \left(c\right) \end{array}\right.$$

The matrix $Q_{11}$ is the solution of a Lyapunov equation (see equation (a)). Lemma D.1 gives an explicit solution: $Q_{11}=\sum_{k=0}^{+\infty }W^{k}\cdot \Sigma \cdot \Sigma^{\prime }\cdot {\left(2L-W^{\prime }\right)}^{-(k+1)}$. Equation (b) leads to

$$Q_{12}=\gamma Q_{11}\cdot {\left(L+\gamma -W^{\prime }\right)}^{-1}=\gamma \sum_{k=0}^{+\infty }W^{k}\cdot \Sigma \cdot \Sigma^{\prime }\cdot {\left(2L-W^{\prime }\right)}^{-(k+1)}\cdot {\left(L+\gamma -W^{\prime }\right)}^{-1}.$$

We see that it does not depend on $\sigma_{z}$, which, once Theorem 2.2 is applied, can be considered null so that the average system $\bar{G}$ corresponds to the original system (17).

Therefore,

(35) $$\bar{G}(W)=-\kappa W+\frac{\mu }{\tau }\left(a_{+}\bar{v}\cdot G_{\gamma }^{\prime }\cdot {\bar{v}}^{\prime }-a_{-}\bar{v}\cdot G_{\gamma }\cdot {\bar{v}}^{\prime }\right)+a_{+}Q_{12}^{\prime }-a_{-}Q_{12}.$$

We show that for **W** already in $E_{p}$, it will stay forever in $E_{p}$: 

1. Inequality W≥0:

Decomposing the connectivity as W=S+iA leads to 〈X,W⋅X〉=〈X,S⋅X〉+i〈X,A⋅X〉. By hermiticity of **S** and **A**, 〈X,S⋅X〉 and 〈X,A⋅X〉 are real numbers. This means we only have to show that the eigenvalues of **S** remain positive along the dynamics. Taking the symmetric part of equation (35) leads to

$$\frac{dS}{dt}=-\kappa S+\frac{\mu (a_{+}-a_{-})}{2\tau }\bar{v}\cdot \left(G_{\gamma }+G_{\gamma }^{\prime }\right)\cdot {\bar{v}}^{\prime }+(a_{+}-a_{-})Q_{22}.$$

Suppose we take an initial condition $S_{0}>0$. It is clear that if $\bar{v}\cdot (G+G^{\prime })\cdot {\bar{v}}^{\prime }$ and $Q_{22}$ are always positive, then **S** will remain positive. This would prove the result. Therefore, focus on 

• Proving $\bar{v}\cdot (G_{\gamma }+G_{\gamma }^{\prime })\cdot {\bar{v}}^{\prime }\geq 0$:

According to the first point of Lemma C.1, $G_{\gamma }+G_{\gamma }^{\prime }=2G_{\gamma }\cdot G_{\gamma }^{\prime }$. Therefore, $\bar{v}\cdot (G_{\gamma }+G_{\gamma }^{\prime })\cdot {\bar{v}}^{\prime }=2\bar{v}\cdot G_{\gamma }\cdot {\left(\bar{v}\cdot G_{\gamma }\right)}^{\prime }$ is a Gramian matrix and therefore it is positive.

• Proving $Q_{22}\geq 0$:

$Q_{22}$ is the covariance matrix of the random value **z**, therefore, it is positive-semi-definite.

2. Inequality ⦀W⦀<lp:

For all $x\in C^{n}$ such that ∥x∥=1, define a family of positive numbers $\left(\alpha_{x}\right)$ whose supremum is written $\alpha^{*}$ and a family of functions $\left(g^{x}\right)$ such that

$$g^{x}:J\to \langle x,J\cdot x\rangle -\alpha_{x}.$$

Because *g* is linear, $dg_{W}^{x}(J)=\langle x,J\cdot x\rangle$. For $W\in {g^{x}}^{-1}(0)$, *i.e.*, $\langle x,W\cdot x\rangle =\alpha_{x}$, compute

$$\begin{array}{rcl} dg_{W}^{x}\left(G^{\mu }(W)\right) & = & -\kappa \underset{=\alpha_{x}}{\underset{︸}{\langle x,W\cdot x\rangle }}+\frac{\mu }{\tau }\underset{=A}{\underset{︸}{\langle x,\bar{v}\cdot \left(a_{+}G_{\gamma }-a_{-}G_{\gamma }^{\prime }\right)\cdot {\bar{v}}^{\prime }\cdot x\rangle }}\\ & & +\left(|a_{+}|+|a_{-}|\right)\underset{=B}{\underset{︸}{\langle x,Q_{12}\cdot x\rangle }}. \end{array}$$

• Upper bound of *A*:

Cauchy-Schwarz leads to

$$|A|\leq |a_{+}|∥\bar{v}\cdot G_{\gamma }\cdot {\bar{v}}^{\prime }\cdot x∥+|a_{-}|∥\bar{v}\cdot G_{\gamma }^{\prime }\cdot {\bar{v}}^{\prime }\cdot x∥.$$

As before, we can use Young’s inequality for convolutions to find an upper bound of *A* which reads

$$A\leq \frac{\tau u_{m}^{2}(|a_{+}|+|a_{-}|)}{{\left(l-\alpha^{*}\right)}^{2}}.$$

• Upper bound of *B*:

 According to Proposition 11.9.3 of [35] the solution of the Lyapunov equation (a) in system (34) can be rewritten

$$Q_{11}=\int_{0}^{+\infty }e^{-t(L-W)}\cdot \Sigma \cdot \Sigma^{t}\cdot e^{-t(L-W^{\prime })}\;dt,$$

because (W−L)⊕(W−L) is not singular due to the fact $W\in E_{p}$.

Observe that for **A** a positive matrix whose eigenvalues are the $\lambda_{i}$, then the spectrum of $e^{-A}$ is $\left\{e^{-\lambda_{i}}:i=1,\ldots ,n\right\}$. Therefore, $⦀e^{-A}⦀=e^{-\min(|\lambda_{i}|)}$. Therefore, if A=L−W, then $⦀e^{-A}⦀\leq e^{\alpha^{*}-l}$. This leads to

$$⦀Q_{11}⦀\leq s^{2}\int_{0}^{+\infty }e^{2(\alpha^{*}-l)t}\;dt=s^{2}{\left[\frac{e^{2(\alpha^{*}-l)t}}{2(\alpha^{*}-l)}\right]}_{0}^{+\infty }=\frac{s^{2}}{2(l-\alpha^{*})}.$$

Then we apply the same arguments to say that

$$\begin{array}{rcl} \left|B\right| & = & ⦀Q_{12}⦀\leq ⦀Q_{11}⦀⦀\gamma {\left(L+\gamma -W\right)}^{-1}⦀\\ & \leq & \frac{s^{2}\gamma }{2(l-\alpha^{*})(l+\gamma -\alpha^{*})}. \end{array}$$

The rest of the proof is identical to the Hebbian case. Assumption 3.1 is changed to Assumption 3.2 for $E_{p}$ to be invariant by the flow $\bar{G}$. □

Define

$$D^{k,q}=\frac{1}{u_{m}^{2}\tau (|a_{+}|+|a_{-}|)}u\cdot G_{l/\mu }^{k+1}\cdot \left(a_{+}G_{\gamma }^{\prime }-a_{-}G_{\gamma }\right)\cdot {G_{l/\mu }^{\prime }}^{k+1}\cdot u^{\prime },$$

such that ${∥D^{k,q}∥}_{2}\leq 1$.

In this framework, one can prove

**Theorem B.12***The correlation term can be written*

$$\begin{array}{rcl} & & \frac{\mu }{\tau }\left(a_{+}\bar{v}\cdot G_{\gamma }^{\prime }\cdot {\bar{v}}^{\prime }-a_{-}\bar{v}\cdot G_{\gamma }\cdot {\bar{v}}^{\prime }\right)\\ & & \;=\frac{u_{m}^{2}(|a_{+}|+|a_{-}|)}{l^{2}}\sum_{k,q=0}^{+\infty }\frac{W^{k}}{l^{k}}\cdot D^{k,q}\cdot \frac{{W^{\prime }}^{q}}{l^{q}}. \end{array}$$

*Proof* Similar to that of Theorem 3.3. □

**Theorem B.13***If Assumption *3.2 *is verified for*$p\leq \frac{1}{3}$, *there is a unique equilibrium point which is globally*, *asymptotically stable*.

*Proof* Similar to the previous case. □

Now, we proceed as before by defining

$$\tilde{p}=\frac{\left|a_{+}|+|a_{-}\right|}{\kappa l^{3}}\left(\frac{s^{2}}{2(\frac{1}{l}+\frac{1}{\gamma })}+u_{m}^{2}\right)\;\text{and}\;\lambda =\frac{s^{2}}{2u_{m}^{2}(\frac{1}{l}+\frac{1}{\gamma })}.$$

**Theorem B.14**

$$\begin{array}{rcl} W & = & \frac{\tilde{p}l}{1+\lambda }\left(\lambda (\alpha_{+}-\alpha_{-})\frac{\Sigma \cdot \Sigma^{\prime }}{d}+D^{0,0}\right)\\ & & +\frac{{\tilde{p}}^{2}l}{{\left(1+\lambda \right)}^{2}}(\lambda^{2}{\left(a_{+}-a_{-}\right)}^{2}\left(1+\frac{1}{1+\gamma /l}\right)\frac{\Sigma \cdot {\Sigma^{\prime }}^{2}}{d^{2}}\\ & & +\lambda \left(\frac{a_{+}-a_{-}}{2}\right)\left(\left(D^{0,0}+2D^{0,1}\right)\cdot \frac{\Sigma \cdot \Sigma^{\prime }}{d}+\frac{\Sigma \cdot \Sigma^{\prime }}{d}\cdot \left(D^{0,0}+2D^{1,0}\right)\right)\\ & & +\frac{\lambda }{1+\gamma /l}\left(a_{+}D^{0,0}\cdot \frac{\Sigma \cdot \Sigma^{\prime }}{d}-a_{-}\frac{\Sigma \cdot \Sigma^{\prime }}{d}\cdot D^{0,0}\right)\\ & & +D^{0,0}\cdot D^{1,0}+D^{1,0}\cdot D^{0,0})+O\left({\tilde{p}}^{3}\right). \end{array}$$

*Proof* First, we need to work on the noise term $Q=a_{+}Q_{12}^{\prime }+a_{-}Q_{12}$. Recall $Q_{11}$ is the solution of the Lyapunov equation $(L-W)\cdot Q_{11}+Q_{11}\cdot {\left(L-W\right)}^{\prime }+\Sigma \cdot \Sigma^{\prime }=0$. Lemma D.1 says that

$$Q_{11}=\sum_{k=0}^{+\infty }W^{k}\cdot \Sigma \cdot \Sigma^{\prime }\cdot {\left(2L-W^{\prime }\right)}^{-(k+1)}$$

is a well-defined solution. We now use the fact that ${\left(2L-W^{\prime }\right)}^{-(k+1)}=\frac{1}{{\left(2l\right)}^{k+1}}\sum_{n=0}^{+\infty }\left(\begin{array}{c} n+k\\ n \end{array}\right)\frac{{W^{\prime }}^{n}}{{\left(2l\right)}^{n}}$ to show that

$$Q_{11}=\sum_{k,n=0}^{+\infty }\frac{1}{{\left(2l\right)}^{k+n+1}}\left(\begin{array}{c} n+k\\ n \end{array}\right)W^{k}\cdot \Sigma \cdot \Sigma^{\prime }\cdot {W^{\prime }}^{n}$$

and therefore

$$Q_{12}=\frac{\gamma }{2l(l+\gamma )}\sum_{k,n,q=0}^{+\infty }\frac{1}{2^{k+n}{\left(1+\gamma /l\right)}^{q}}\left(\begin{array}{c} n+k\\ n \end{array}\right)\frac{W^{k}}{l^{k}}\cdot \Sigma \cdot \Sigma^{\prime }\cdot \frac{{W^{\prime }}^{n+q}}{l^{n+q}}.$$

Thus, writing $\alpha_{\pm }=\frac{a_{\pm }}{\left|a_{+}|+|a_{-}\right|}$ and $c_{k,n,q}=\frac{1}{2^{k+n}{\left(1+\gamma /l\right)}^{q}}\left(\begin{array}{c} n+k\\ n \end{array}\right)$, the noise term is

$$\begin{array}{rcl} Q & = & \frac{d(|a_{+}|+|a_{-}|)}{2l^{2}(\frac{1}{l}+\frac{1}{\gamma })}\sum_{k,n,q=0}^{+\infty }c_{k,n,q}(\alpha_{+}\frac{W^{n+q}}{l^{n+q}}\cdot \frac{\Sigma \cdot \Sigma^{\prime }}{d}\cdot \frac{{W^{\prime }}^{k}}{l^{k}}\\ & & -\alpha_{-}\frac{W^{k}}{l^{k}}\cdot \frac{\Sigma \cdot \Sigma^{\prime }}{d}\cdot \frac{{W^{\prime }}^{n+q}}{l^{n+q}}). \end{array}$$

Define $\Omega =\frac{W}{\tilde{p}l}$ such that ⦀Ω⦀=O(1). We improperly write $\bar{G}(\Omega )=\bar{G}(\tilde{p}l\Omega )$ such that

$$\begin{array}{rcl} \bar{G}(\Omega ) & = & -\tilde{p}l\kappa \Omega +\frac{u_{m}^{2}(|a_{+}|+|a_{-}|)}{l^{2}}\sum_{k,q=0}^{+\infty }{\left(\tilde{p}\Omega \right)}^{k}\cdot D^{k,q}\cdot {\left(\tilde{p}\Omega \right)}^{q}\\ & & +\frac{d(|a_{+}|+|a_{-}|)}{2l^{2}(\frac{1}{l}+\frac{1}{\gamma })}\sum_{k,n,q=0}^{+\infty }c_{k,n,q}(\alpha_{+}{\left(\tilde{p}\Omega \right)}^{n+q}\cdot \frac{\Sigma \cdot \Sigma^{\prime }}{d}\cdot {\left(\tilde{p}\Omega^{\prime }\right)}^{k}\\ & & -\alpha_{-}{\left(\tilde{p}\Omega \right)}^{k}\cdot \frac{\Sigma \cdot \Sigma^{\prime }}{d}\cdot {\left(\tilde{p}\Omega^{\prime }\right)}^{n+q}). \end{array}$$

This leads to

$$\begin{array}{rcl} \bar{G}(\Omega ) & = & \left(\frac{u_{m}^{2}(|a_{+}|+|a_{-}|)}{l^{2}}+\frac{d(|a_{+}|+|a_{-}|)}{2l^{2}(\frac{1}{l}+\frac{1}{\gamma })}\right)\\ & & \times [-\Omega +\frac{1}{1+\lambda }\overset{\tilde{F}}{\overset{︷}{\sum_{k,q=0}^{+\infty }{\left(\tilde{p}\Omega \right)}^{k}\cdot D^{k,q}\cdot {\left(\tilde{p}\Omega \right)}^{q}}}+\frac{\lambda }{1+\lambda }\\ & & \times \underset{\tilde{Q}}{\underset{︸}{\sum_{k,n,q=0}^{+\infty }c_{k,n,q}\left(\alpha_{+}{\left(\tilde{p}\Omega \right)}^{n+q}\cdot \frac{\Sigma \cdot \Sigma^{\prime }}{d}\cdot {\left(\tilde{p}\Omega^{\prime }\right)}^{k}-\alpha_{-}{\left(\tilde{p}\Omega \right)}^{k}\cdot \frac{\Sigma \cdot \Sigma^{\prime }}{d}\cdot {\left(\tilde{p}\Omega^{\prime }\right)}^{n+q}\right)}}]. \end{array}$$

We are looking for $F_{a}$ and $Q_{a}$ in the expansions $\tilde{F}=\sum_{a=0}^{+\infty }F_{a}{\tilde{p}}^{a}$ and $\tilde{Q}=\sum_{a=0}^{+\infty }Q_{a}{\tilde{p}}^{a}$. Recall

$$\Omega^{p}=\sum_{i=0}^{+\infty }{\tilde{p}}^{i}\sum_{\eta \in N^{p},\sum_{k}\eta_{k}=i}\Omega_{\eta_{1}}\cdot \Omega_{\eta_{2}}\cdot \ldots \cdot \Omega_{\eta_{p}}.$$

Therefore,

$$\begin{array}{rcl} \tilde{Q} & = & \sum_{k,n,q,i,j=0}^{+\infty }c_{k,n,q}{\tilde{p}}^{k+n+q+i+j}\underset{\theta \in N^{n+q},\sum_{m}\theta_{m}=j}{\sum_{\eta \in N^{k},\sum_{m}\eta_{m}=i}}\alpha_{+}\Omega_{\eta_{1}}\cdot \ldots \cdot \Omega_{\eta_{n+q}}\\ & & \times \frac{\Sigma \cdot \Sigma^{\prime }}{d}\cdot \Omega_{\theta_{1}}^{\prime }\cdot \ldots \cdot \Omega_{\theta_{k}}^{\prime }-\alpha_{-}\Omega_{\eta_{1}}\cdot \ldots \cdot \Omega_{\eta_{k}}\cdot \frac{\Sigma \cdot \Sigma^{\prime }}{d}\cdot \Omega_{\theta_{1}}^{\prime }\cdot \ldots \cdot \Omega_{\theta_{n+q}}^{\prime }. \end{array}$$

Leading to

$$\begin{array}{rcl} Q_{a} & = & \overset{+\infty }{\underset{a=k+n+q+i+j}{\sum_{k,n,i,j=0}}}c_{k,n,q}{\tilde{p}}^{k+n+q+i+j}\underset{\theta \in N^{n+q},\sum_{m}\theta_{m}=j}{\sum_{\eta \in N^{k},\sum_{m}\eta_{m}=i}}\alpha_{+}\Omega_{\eta_{1}}\cdot \ldots \cdot \Omega_{\eta_{n+q}}\\ & & \times \frac{\Sigma \cdot \Sigma^{\prime }}{d}\cdot \Omega_{\theta_{1}}^{\prime }\cdot \ldots \cdot \Omega_{\theta_{k}}^{\prime }-\alpha_{-}\Omega_{\eta_{1}}\cdot \ldots \cdot \Omega_{\eta_{k}}\cdot \frac{\Sigma \cdot \Sigma^{\prime }}{d}\cdot \Omega_{\theta_{1}}^{\prime }\cdot \ldots \cdot \Omega_{\theta_{n+q}}^{\prime }. \end{array}$$

This equation is scary but it reduces to simple expressions for small a∈N.

$$\begin{array}{ccc} a & Q_{a} & F_{a}\\ 0 & (\alpha_{+}-\alpha_{-})\frac{\Sigma \cdot \Sigma^{\prime }}{d} & D^{0,0}\\ 1 & \frac{\alpha_{+}-\alpha_{-}}{2}(\Omega_{0}\cdot \frac{\Sigma \cdot \Sigma^{\prime }}{d}+\frac{\Sigma \cdot \Sigma^{\prime }}{d}\cdot \Omega_{0}^{\prime })+\frac{1}{1+\gamma /l}(\alpha_{+}\Omega_{0}\cdot \frac{\Sigma \cdot \Sigma^{\prime }}{d}-\alpha_{-}\frac{\Sigma \cdot \Sigma^{\prime }}{d}\cdot \Omega_{0}^{\prime }) & \Omega_{0}\cdot D^{1,0}+D^{0,1}\cdot \Omega_{0}^{\prime } \end{array}$$

Recall that $W=\tilde{p}l\Omega =\tilde{p}l(\frac{1}{1+\lambda }\tilde{F}+\frac{\lambda }{1+\lambda }\tilde{Q})$ to get the result. □

## Appendix C: Properties of the convolution operators $G_{\gamma }$, W, and V

Recall $G_{\gamma }$, W, and V are convolution operators respectively generated by $g_{\gamma }$, *v*, and *w* defined in (30). Their Fourier transforms are respectively

$$\begin{array}{rcl} {\hat{g}}_{\gamma }:\xi & \mapsto & \frac{\gamma }{\gamma +2i\pi \xi },\\ \hat{v}:\xi & \mapsto & \frac{4\beta }{\left(\beta (1+\Delta )+4i\pi \mu \xi )(\beta (1-\Delta )+4i\pi \mu \xi \right)},\\ \hat{w}:\xi & \mapsto & \frac{4\beta +8i\pi \mu \xi }{\left(\beta (1+\Delta )+4i\pi \mu \xi )(\beta (1-\Delta )+4i\pi \mu \xi \right)}. \end{array}$$

### C.1 Algebraic properties

**Lemma C.1**

$$\frac{G_{\gamma }+G_{\gamma }^{\prime }}{2}=G_{\gamma }\cdot G_{\gamma }^{\prime }.$$

*Proof* Compute

$$\begin{array}{rcl} {\left(G_{\gamma }\cdot G_{\gamma }^{\prime }\right)}_{xy} & = & \gamma^{2}\int_{-\infty }^{+\infty }e^{-\gamma (x-z)}H(x-z)e^{-\gamma (y-z)}H(y-z)\;dz\\ & = & \gamma^{2}e^{-\gamma (x+y)}\int_{-\infty }^{\min(x,y)}e^{2\gamma z}\;dz=\gamma^{2}e^{-\gamma (y+x)}{\left[\frac{e^{2\gamma z}}{2\gamma }\right]}_{-\infty }^{\min(x,y)}\\ & = & \frac{\gamma }{2}e^{-\gamma (y+x-2\min(x,y))}. \end{array}$$

Therefore, if y≥x, then ${\left(G_{\gamma }\cdot G_{\gamma }^{\prime }\right)}_{xy}=\frac{\gamma }{2}e^{-\gamma (y-x)}$, and if x≥y, then ${\left(G_{\gamma }\cdot G_{\gamma }^{\prime }\right)}_{xy}=\frac{\gamma }{2}e^{-\gamma (x-y)}$. The result follows. □

**Lemma C.2**

$$G_{\gamma }^{\prime }-G_{\gamma }=\frac{1}{\gamma }D\cdot \left(G_{\gamma }^{\prime }+G_{\gamma }\right),$$

*where*D*is the time*-*differentiation operator*, *i*.*e*., $\left(X\cdot D)(t)=\frac{dX}{dt}(t\right)$.

*Proof*$G_{\gamma }$ and $G_{\gamma }^{\prime }$ are two convolution operators respectively generated by $g_{\gamma }:t\mapsto \gamma e^{-\gamma t}H(t)$ and $g_{\gamma }^{\prime }:t\mapsto \gamma e^{\gamma t}H(-t)$. The Fourier transform of $g_{\gamma }$ is $\hat{h}(\xi )=\frac{\gamma }{\gamma +2i\pi \xi }$. Therefore, the Fourier transform of $g_{\gamma }^{\prime }-g_{\gamma }$ is

$$\begin{array}{rcl} \hat{g_{\gamma }^{\prime }-g_{\gamma }}(\xi ) & = & \frac{\gamma }{\gamma -2i\pi \xi }-\frac{\gamma }{\gamma +2i\pi \xi }=\frac{2i\pi \xi }{\gamma }\frac{2\gamma^{2}}{\gamma^{2}+4\pi^{2}\xi^{2}}\\ & = & \frac{2i\pi \xi }{\gamma }\left(\frac{\gamma }{\gamma +2i\pi \xi }+\frac{\gamma }{\gamma -2i\pi \xi }\right)=\frac{2i\pi \xi }{\gamma }\left(\hat{g_{\gamma }^{\prime }+g_{\gamma }}(\xi )\right). \end{array}$$

Because $\hat{\frac{df}{dt}}(\xi )=2i\pi \xi \hat{f}$, taking the inverse Fourier transform of $\hat{g_{\gamma }^{\prime }-g_{\gamma }}(\xi )$ gives the result. □

**Lemma C.3**

$$W\cdot V^{k}\cdot G_{\beta /\mu }=V^{k+1}.$$

*Besides*, *if*Δ∈iR, $V^{k}$*is a convolution operator generated by*

$$v_{k}:t\mapsto \frac{\sqrt{\pi \beta }}{k!}e^{-\frac{\beta }{2}t}{\left(\frac{t}{\left|\Delta \right|}\right)}^{k+\frac{1}{2}}J_{k+\frac{1}{2}}\left(\frac{\beta |\Delta |}{2}t\right)H(t),$$

*where*$J_{n}(z)$*is the Bessel function of the first kind*. *If*Δ∈R, *the formula above holds if one replaces*$J_{n}(z)$*by*$I_{n}(z)$, *the modified Bessel function of the first kind*.

*Proof* We want to compute $W\cdot V^{k}\cdot G_{\beta /\mu }$. Compute the Fourier transform of $w*v_{k}*g_{\beta /\mu }$, where $v_{k}$ is the result of k convolutions of *v* with itself

$$\begin{array}{rcl} \hat{w*v_{k}*g_{\frac{\beta }{\mu }}}(\xi ) & = & \hat{w}(\xi ){\hat{g}}_{\frac{\beta }{\mu }}(\xi )\hat{v_{k}}(\xi )\\ & = & {\left(\frac{\beta }{\left(\frac{\beta (1+\Delta )}{2}+2i\pi \mu \xi )(\frac{\beta (1-\Delta )}{2}+2i\pi \mu \xi \right)}\right)}^{k+1}={\hat{v}}^{k+1}(\xi ). \end{array}$$

This proves the first result.

Then observe that

$$\begin{array}{rcl} v_{k+1}(t) & = & \beta^{k+1}F^{-1}\left(\xi \mapsto \frac{1}{{\left(\frac{\beta (1+\Delta )}{2}+2i\pi \mu \xi \right)}^{k+1}}\right)\\ & & *F^{-1}\left(\xi \mapsto \frac{1}{{\left(\frac{\beta (1-\Delta )}{2}+2i\pi \mu \xi \right)}^{k+1}}\right)(t)\\ & = & \beta^{k+1}\left(s\mapsto \frac{s^{k}}{k!}e^{-\frac{\beta (1+\Delta )}{2}s}H(s)\right)*\left(s\mapsto \frac{s^{k}}{k!}e^{-\frac{\beta (1-\Delta )}{2}s}H(s)\right)(t)\\ & = & \frac{\beta^{k+1}}{k{!}^{2}}e^{-\frac{\beta (1-\Delta )}{2}t}\int_{0}^{t}s^{k}{\left(t-s\right)}^{k}e^{-\beta \Delta s}\;dsH(t). \end{array}$$

The last integral can be analytically computed with the help of Bessel functions. In fact, it gives different results depending on the nature of Δ (whether it is real or imaginary). 

• If Δ∈R, then defining $I_{n}(z)$, the modified Bessel function of the first kind, leads to

$$\int_{0}^{t}e^{-\beta \Delta s}s^{k}{\left(t-s\right)}^{k}\;ds=\sqrt{\pi }e^{-\frac{\beta \Delta }{2}t}k!{\left(\frac{t}{\beta \Delta }\right)}^{k+\frac{1}{2}}I_{k+\frac{1}{2}}\left(\frac{\beta \Delta }{2}t\right).$$

• If Δ∈iR, then defining $J_{n}(z)$, the Bessel function of the first kind, leads to

$$\int_{0}^{t}e^{-\beta \Delta s}s^{k}{\left(t-s\right)}^{k}\;ds=\sqrt{\pi }e^{-\frac{\beta \Delta }{2}t}k!{\left(\frac{t}{\beta |\Delta |}\right)}_{k+\frac{1}{2}}^{k+\frac{1}{2}}\left(\frac{\beta |\Delta |}{2}t\right).$$

This concludes the proof. □

### C.2 Signed integral

1. $\int_{-\infty }^{+\infty }g_{\gamma }(t)\;dt=\gamma \frac{0-1}{-\gamma }=1$.

2. For $\Delta =\sqrt{1-\frac{4l}{\beta }}\in C$, compute

$$\begin{array}{rcl} \int_{-\infty }^{+\infty }v(t)\;dt & = & \frac{l}{\Delta \mu }\left(\int_{0}^{+\infty }e^{-\frac{\beta }{2\mu }(1-\Delta )t}\;dt-\int_{0}^{+\infty }e^{-\frac{\beta }{2\mu }(1-\Delta )t}\;dt\right)\\ & = & \frac{l}{\Delta \mu }\left(\frac{0-1}{-\frac{\beta }{2\mu }(1-\Delta )}-\frac{0-1}{-\frac{\beta }{2\mu }(1+\Delta )}\right)\\ & = & \frac{2l}{\Delta \beta }\frac{1+\Delta -(1-\Delta )}{1-\Delta^{2}}=\frac{4l}{\beta }\frac{\beta }{4l}=1. \end{array}$$

3. Similarly,

$$\begin{array}{rcl} \int_{-\infty }^{+\infty }w(t)\;dt & = & \frac{l}{2\Delta \mu }((1+\Delta )\int_{0}^{+\infty }e^{-\frac{\beta }{2\mu }(1-\Delta )t}\;dt\\ & & -(1-\Delta )\int_{0}^{+\infty }e^{-\frac{\beta }{2\mu }(1-\Delta )t}\;dt)\\ & = & \frac{l}{\Delta \beta }\frac{{\left(1+\Delta \right)}^{2}-{\left(1-\Delta \right)}^{2}}{1-\Delta^{2}}\\ & = & \frac{l}{\Delta \beta }\frac{4\Delta \beta }{4l}=1. \end{array}$$

### C.3 L1 norm

• For 4l≤β, *i.e.*, $\Delta =\sqrt{1-\frac{4l}{\beta }}\in R_{+}$, then 

1. $g_{\gamma }(t)>0$ and ${∥g_{\gamma }∥}_{1}=\int_{R}g_{\gamma }(t)\;dt=1$.

2. $v(t)=\frac{2l}{\Delta \mu }e^{-\frac{\beta }{2\mu }t}\mathrm{sh}(\frac{\beta \Delta }{2\mu }t)H(t)\geq 0$ and ${∥v∥}_{1}=\int_{R}v(t)\;dt=1$.

3. $w(t)=\frac{l}{\Delta \mu }e^{-\frac{\beta }{2\mu }t}(\mathrm{sh}(\frac{\beta \Delta }{2\mu }t)+\Delta \mathrm{ch}(\frac{\beta \Delta }{2\mu }t))H(t)\geq 0$ and ${∥u∥}_{1}=\int_{R}u(t)\;dt=1$.

• For 4l>β, *i.e.*, Δ is a pure imaginary, we rewrite $\Delta =\sqrt{\frac{4l}{\beta }-1}$ and observe that 

1. $g_{\gamma }(t)>0$ and ${∥g_{\gamma }∥}_{1}=\int_{R}g_{\gamma }(t)\;dt=1$.

2. $v(t)=\frac{2l}{\Delta \mu }e^{-\frac{\beta }{2\mu }t}\sin(\frac{\beta \Delta }{2\mu }t)H(t)$ which changes sign on $R_{+}$. Therefore,

$$\begin{array}{rcl} {∥v∥}_{1} & = & \frac{2l}{\Delta \mu }\int_{0}^{+\infty }e^{-\frac{\beta }{2\mu }t}\left|\sin\left(\frac{\beta \Delta }{2\mu }t\right)\right|\;dt=\frac{4l}{\Delta^{2}\beta }\int_{0}^{+\infty }e^{-\frac{s}{\Delta }}\left|\sin(s)\right|\;ds\\ & = & \frac{4l}{\Delta^{2}\beta }\sum_{k=0}^{+\infty }{\left(-1\right)}^{k}\int_{k\pi }^{(k+1)\pi }e^{-\frac{s}{\Delta }}\sin(s)\;ds\\ & = & \frac{4l}{\Delta^{2}\beta }\sum_{k=0}^{+\infty }{\left(-1\right)}^{k}\frac{{\left(-1\right)}^{k}e^{-\frac{k\pi }{\Delta }}-{\left(-1\right)}^{k+1}e^{-\frac{(k+1)\pi }{\Delta }}}{1+\frac{1}{\Delta^{2}}}\\ & = & \left(1+e^{-\frac{\pi }{\Delta }}\right)\sum_{k=0}^{+\infty }e^{-\frac{k\pi }{\Delta }}=\frac{1+e^{-\frac{\pi }{\Delta }}}{1-e^{-\frac{\pi }{\Delta }}}=\coth\left(\frac{\pi }{2\Delta }\right). \end{array}$$

3. $w(t)=\frac{l}{\Delta \mu }e^{-\frac{\beta }{2\mu }t}(\sin(\frac{\beta \Delta }{2\mu }t)+\Delta \cos(\frac{\beta \Delta }{2\mu }t))H(t)$ which also changes sign on $R_{+}$. We have not found a way to compute ${∥w∥}_{1}$ and write the result elegantly.

## Appendix D: Solution of a Lyapunov equation

**Lemma D.1***The solution of the following Lyapunov equation*

$$(L-W)\cdot X+X\cdot {\left(L-W\right)}^{\prime }+D=0,$$

*where*L=lId*is*

(36) $$X=-\sum_{k=0}^{+\infty }W^{k}\cdot D\cdot {\left(2L-W^{\prime }\right)}^{-(k+1)}.$$

*Proof* First, observe that if {|λ|:λ eigenvalue of W}∈]0,l[ and W>0, then $⦀W⦀⦀{\left(2L-W\right)}^{-1}⦀<1$. Therefore, **X** is well defined by equation (36).

Observe that ${\left(2L-W^{\prime }\right)}^{-1}\cdot {\left(L-W\right)}^{\prime }=I_{d}-L\cdot {\left(2L-W^{\prime }\right)}^{-1}$. Assuming **X** is defined by equation (36), then based on the fact **L** commutes with any matrix (because it is a scalar matrix),

$$\begin{array}{rcl} & & (L-W)\cdot X+X\cdot {\left(L-W\right)}^{\prime }\\ & & \;=-(L\cdot \sum_{k=0}^{+\infty }W^{k}\cdot D\cdot {\left(2L-W^{\prime }\right)}^{-(k+1)}-W\cdot \sum_{k=0}^{+\infty }W^{k}\cdot D\cdot {\left(2L-W^{\prime }\right)}^{-(k+1)}\\ & & \;+\sum_{k=0}^{+\infty }W^{k}\cdot D\cdot {\left(2L-W^{\prime }\right)}^{-k}-L\cdot \sum_{k=0}^{+\infty }W^{k}\cdot D\cdot {\left(2L-W^{\prime }\right)}^{-(k+1)})\\ & & \;=-\left(\sum_{k=0}^{+\infty }W^{k}\cdot D\cdot {\left(2L-W^{\prime }\right)}^{-k}-\sum_{k=0}^{+\infty }W^{k+1}\cdot D\cdot {\left(2L-W^{\prime }\right)}^{-(k+1)}\right)=-D. \end{array}$$

 □

## Competing interests

The authors declare that they have no competing interests.

## Authors’ contributions

GW developed the theory of temporal averaging presented in this paper. MG applied this theory to learning neural networks and did the numerical simulations. Both authors read and approved the final manuscript.
